# Supplementary material for: Phase I trial of WEE1 inhibition with chemotherapy and radiotherapy as adjuvant treatment, and a window of opportunity trial with cisplatin in patients with head and neck cancer: the WISTERIA trial protocol
Source: BMJ Open. 2020 Mar 16;10(3):e033009. doi: 10.1136/bmjopen-2019-033009 (PMC7076237; doi:10.1136/bmjopen-2019-033009)
Supplement: Supplementary data [file bmjopen-2019-033009supp001.pdf]

Appendix 1: Informed Consent Form – Group A

To be printed on hospital headed paper

Informed Consent Form

To be used for Group A only

WISTERIA

A Phase I trial of WEE1 inhibition with Chemotherapy and Radiotherapy as adjuvant treatment, and a Window of Opportunity trial with Cisplatin in Patients with Head and Neck Cancer

EudraCT Reference: 2015-003583-37

Site:

Patient Trial Number: ☐☐☐

Principal Investigator: ____________________

Screening Number: SCR ☐☐ / ☐☐
(If applicable)

Please initial each box

1. I confirm that I have read and understand the Patient Information Sheet Group A (version .... dated ....) for the above trial. I have had the opportunity to consider the information, ask questions and have had these answered satisfactorily. ☐

2. I understand that my participation is voluntary and that I am free to withdraw at any time without giving any reason, without my medical care or legal rights being affected. I understand that if I withdraw from treatment my doctor may continue to provide the Trial Office with information that would routinely be collected about me and recorded in my medical notes. I am aware that I can also withdraw consent for this data transfer. ☐

3. I give permission for my initials, date of birth, and NHS number to be given to the WISTERIA Trial Office when I am registered to the trial as well as a copy of this consent form. ☐

4. I understand that relevant sections of my medical notes and data collected during the trial may be looked at by individuals from the WISTERIA Trial Office, regulatory authorities, Sponsor and/or NHS bodies, where it is relevant to my taking part in this research. I understand that this information will be held in a confidential manner. I give permission for these individuals to have access to my records. ☐

5. I understand that anonymised data from the trial may be provided to other third parties (e.g. pharmaceutical companies or other academic institutions) for research, safety monitoring or licensing purposes. I understand that this may involve sending data outside of the United Kingdom to a European country or the United States of America and that my name will not be given to these third parties.

6. I agree to my GP being informed of my participation in this trial.

7. I understand that the WISTERIA Trial Office may access information held by national cancer registries and within national databases to keep in touch with me and to follow up on my health status.

8. I give permission for collection of samples of my blood and tissue to be used in the WISTERIA trial. I understand that samples will be sent to the Institute of Head and Neck Studies and Education (InHANSE), University of Birmingham and other laboratories in the United Kingdom or overseas (including Covance Laboratories Inc., based in the United States of America).

9. I understand that DNA analysis may be performed on the samples taken for the trial.

10. I consent to the storage of samples remaining at the end of the trial and their use in future ethically approved research which may involve genetic analysis, animal or *in vitro* models, commercial or private institutions, and which may take place in the UK or overseas.

11. I understand that information which may identify me will be transferred outside of the hospital and to the <insert name> Clinical Trials Unit at <insert location>. I give permission for this information to be transferred and for the information, including a copy of this consent form to be held by the Clinical Trials Unit so long as strict confidentiality is maintained.
*To be deleted as appropriate

12. I agree to take part in the above trial.

**Name of patient**	**Date**	**Signature**
**Name of person taking consent**	**Date**	**Signature**

You must have signed the
Site Signature & Delegation Log

This document was written using CRCTU-ICF-QCD-001, Version 2.0

CANCER RESEARCH UK

Appendix 2: Informed Consent Form – Group B

To be printed on hospital headed paper

Informed Consent Form

To be used for Group B only

WISTERIA

A Phase I trial of WEE1 inhibition with Chemotherapy and Radiotherapy as adjuvant treatment, and a Window of Opportunity trial with Cisplatin in Patients with Head and Neck Cancer

EudraCT Reference: 2015-003583-37

Site: ____________________ **Patient Trial Number:** ☐☐☐

Principal Investigator: ____________________

Please initial each box

13. I confirm that I have read and understand the Patient Information Sheet Group B (version .... dated ....) for the above trial. I have had the opportunity to consider the information, ask questions and have had these answered satisfactorily. ☐
14. I understand that my participation is voluntary and that I am free to withdraw at any time without giving any reason, without my medical care or legal rights being affected. I understand that if I withdraw from treatment my doctor may continue to provide the Trial Office with information that would routinely be collected about me and recorded in my medical notes. I am aware that I can withdraw consent for this data transfer. ☐
15. I give permission for my initials, date of birth, and NHS number to be given to the WISTERIA Trial Office when I am registered to the trial as well as a copy of this consent form. ☐
16. I understand that relevant sections of my medical notes and data collected during the trial may be looked at by individuals from the WISTERIA Trial Office, regulatory authorities, Sponsor and/or NHS bodies, where it is relevant to my taking part in this research. I understand that this information will be held in a confidential manner. I give permission for these individuals to have access to my records. ☐

17. I understand that anonymised data from the trial may be provided to other third parties (e.g. pharmaceutical companies or other academic institutions) for research, safety monitoring or licensing purposes. I understand that this may involve sending data outside of the United Kingdom to a European country or the United States of America and that my will not be given to these third parties. ☐
18. I agree to my GP being informed of my participation in this trial. ☐
19. I understand that the WISTERIA Trial Office may access information held by national cancer registries and within national databases to keep in touch with me and to follow up on my health status. ☐
20. I give permission for the collection of samples of my blood and tissue to be used in the WISTERIA trial. I understand that samples will be sent to the Institute of Head and Neck Studies and Education (InHANSE), University of Birmingham and other laboratories in the United Kingdom or overseas (including Covance Laboratories Inc., based in the United States of America). ☐
21. I understand that DNA analysis may be performed on the samples taken for the trial. ☐
22. I consent to the storage of samples remaining at the end of the trial and their use in future ethically approved research which may involve genetic analysis, animal or *in vitro* models, commercial or private institutions, and which may take place in the UK or overseas. ☐
23. I understand that information which may identify me will be transferred outside of the hospital and to the <insert name> Clinical Trials Unit at <insert location>. I give permission for this information to be transferred and for the information, including a copy of this consent form to be held by the Clinical Trials Unit so long as strict confidentiality is maintained. *To be deleted as appropriate ☐
24. I agree to take part in the above trial. ☐

**Name of patient**	**Date**	**Signature**
**Name of person taking consent**<br>You must have signed the<br>Site Signature & Delegation Log	**Date**	**Signature**

This document was written using CRCTU-ICF-QCD-001, Version 2.0

CANCER RESEARCH UK

Appendix 3: Wisteria Radiotherapy Guidelines – Larynx

Volume	Definition and description	
**CTV_6500**	Include the operative bed if a positive margin is present, and the nodal levels with extracapsular spread (ECS), if present. The extent of the operative bed is defined with reference to pre-operative imaging of the primary tumour and to the operative pathology.<br><br>Primary tumour: The superior border will lie at the level corresponding to the hyoid on the pre-operative imaging for glottic and subglottic tumours, or 1 cm above the most cranial aspect of the tumour, whichever is most cranial. For supraglottic tumours, the superior border will lie at the level of the tip of the epiglottis on pre-operative imaging, or 1 cm superior to the most cranial aspect of the tumour, whichever is most cranial. The inferior border will lie at the level corresponding with the caudal aspect of the cricoid cartilage on pre-operative imaging, or 1 cm inferior to the most caudal aspect of the tumour, whichever is most caudal. The stoma is included in CTV_6500 if there is subglottic extension of the primary tumour and a positive margin, or ECS in level IV.<br><br>Nodes: For areas of nodal ECS, the whole level should be included. If a node with ECS is present at a border between two lymph node levels, the level above/below should also be included as appropriate. For nodal levels included in the CTV_6500, the overlying sternocleidomastoid muscle (SCM) should be included.	
**CTV_6000**	Include:<br>1. The operative bed if there is no positive margin, as defined above.<br>2. Dissected lymph node levels in which there is no ECS if that side of the neck is pN+. The whole level should be included. The SCM should not be included.<br>3. The stoma if there are lymph nodes without ECS in level IV, subglottic extension without a positive margin, or an emergency tracheostomy was performed.	
**CTV_5400**	IA	Not included
	IB	Included if ipsilateral level II is node positive
	II	Always included bilaterally if not already included in CTV_6500 or CTV_6000. If ipsilateral neck is node negative, superior border lies at the inferior border of the transverse process of C1. If ipsilateral neck is node positive, level II is extended superiorly to skull base to include retrostyloid space.
	III	Always included bilaterally
	IVa	Always included bilaterally
	V	Included if ipsilateral neck is node positive
	VI	Included if there is subglottic extension of the primary tumour, soft tissue extension from the primary into the neck, or hypopharyngeal involvement
	RP*	Included if bulky ipsilateral nodes present
	Stoma	If not included in CTV_6500/6000 as defined above

* In the WISTERIA trial, we recommend that the cranial border of the retropharyngeal nodal level is defined as the upper edge of the body of C1 or the upper extent of the hard palate, whichever is more cranial.

Appendix 4: Wisteria Radiotherapy Guidelines – Hypopharynx

Volume	Definition and description	
**CTV_6500**	Include the operative bed if a positive margin is present, and the nodal levels with ECS, if present. The extent of the operative bed is defined with reference to pre-operative imaging of the primary tumour and to the operative pathology.<br><br>Primary tumour: The superior border will lie at corresponding to the level of the hyoid on pre-operative or 1 cm above the most cranial aspect of the tumour on pre-operative imaging, whichever is most cranial. The inferior border will lie at the level corresponding with the caudal aspect of the cricoid cartilage on pre-operative imaging, or 1 cm inferior to the most caudal aspect of the tumour, whichever is most caudal. The stoma (if present) is included in CTV_6500 if there is subglottic extension of the primary tumour and a positive margin, or ECS in level IV.<br><br>Nodes: For areas of nodal ECS, the whole level should be included. If a node with ECS is present at a border between two lymph node levels, the level above/below should also be included as appropriate. For nodal levels included in the CTV_6500, the overlying sternocleidomastoid muscle (SCM) should be included.	
**CTV_6000**	Include:<br>1. The operative bed if there is no positive margin, as defined above.<br>2. Dissected lymph node levels in which there is no ECS if that side of the neck is pN+. The whole level should be included. The SCM should not be included.<br>3. The stoma (if present) if there are lymph nodes without ECS in level IV, subglottic extension without a positive margin, or an emergency tracheostomy was performed.	
**CTV_5400**	IA	Not included
	IB	Included if ipsilateral level II is node positive
	II	Always included bilaterally if not already included in CTV_6500 or CTV_6000. If ipsilateral neck is node negative, superior border lies at the inferior border of the transverse process of C1. If ipsilateral neck is node positive, level II is extended superiorly to skull base to include retrostyloid space.
	III	Always included bilaterally
	IV	Always included bilaterally
	V	Included if ipsilateral neck is node positive
	VI	Included if there is subglottic/oesophageal extension of the primary tumour, or soft tissue extension from the primary into the neck
		Included
	RP*	If not included in CTV_6500/6000 as defined above
	Stoma	Retropharyngeal (RP)

* In the WISTERIA trial, we recommend that the cranial border of the retropharyngeal nodal level is defined as the upper edge of the body of C1 or the upper extent of the hard palate, whichever is more cranial.

Appendix 5: Wisteria Radiotherapy Guidelines – Lateralised Oral Cavity

<table>
<tr><th>Volume</th><th colspan="2">Definition and description</th></tr>
<tr><td>CTV_6500</td><td colspan="2">Include the operative bed if a positive margin is present, and the nodal levels with ECS, if present. The extent of the operative bed is defined with reference to pre-operative imaging of the primary tumour and to the operative pathology.

Primary tumour: The volume will depend on the primary site. For oral tongue tumours, include the hemi-oral cavity including base of tongue. For buccal mucosal tumours, include the entire mucosa including the retromolar trigone. For retromolar trigone tumours, include the preoperative tumour volume and postoperative tumour bed. Exclude uninvolved bone where possible. Generally, the superior border will be at the level of the hard palate/inferior orbital rim and the inferior border at the level of the hyoid.

Nodes: For areas of nodal ECS, the whole level should be included. If a node with ECS is present at a border between two lymph node levels, the level above/below should also be included as appropriate. For nodal levels included in the CTV_6500, the overlying sternocleidomastoid muscle (SCM) should be included.</td></tr>
<tr><td>CTV_6000</td><td colspan="2">Include:
1. The operative bed if there is no positive margin, as defined above.
2. Dissected lymph node levels in which there is no ECS if that side of the neck is pN+. The whole level should be included. The SCM should not be included.</td></tr>
<tr><td>CTV_5400</td><td>IA
IB

II

III
IV

V
VI
RP*</td><td>Included if tumour involves floor of mouth or anterior mandible
Always included ipsilaterally if not already included in CTV_6500 or CTV_6000
Always included ipsilaterally if not already included in CTV_6500 or CTV_6000. If ipsilateral neck is node negative, superior border lies at the inferior border of the transverse process of C1. If ipsilateral neck is node positive, level II is extended superiorly to skull base. Include contralateral nodes if pN2b/pN3 or primary depth of invasion > 4mm.
Always included ipsilaterally
Included ipsilaterally if the oropharynx or anterior tongue are involved
Included if ipsilateral neck is node positive
Not included
Included if retromolar trigone primary
Not included
Included if retromolar trigone primary</td></tr>
</table>

* In the WISTERIA trial, we recommend that the cranial border of the retropharyngeal nodal level is defined as the upper edge of the body of C1 or the upper extent of the hard palate, whichever is more cranial.

Please note: Laterality or otherwise the tumour should be discussed with the surgeon, radiologist and pathologist and confirmed at MDT.

Appendix 6: Wisteria Radiotherapy Guidelines – Non-Lateralised Oral Cavity

Volume	Definition and description	
**CTV_6500**	Include the operative bed if a positive margin is present, and the nodal levels with ECS, if present. The extent of the operative bed is defined with reference to pre-operative imaging of the primary tumour and to the operative pathology.<br><br><u>Primary tumour</u>: The volume will depend on the primary site. For oral tongue tumours, include the whole oral cavity including base of tongue. Exclude uninvolved bone where possible, but for floor of mouth tumours, consider including the alveolar ridge. Generally, the superior border will be at the level of the hard palate/inferior orbital rim and the inferior border at the level of the hyoid. If a mouth bite is used for floor of mouth tumours, the superior border may be located at the midpoint of the mouthbite.<br><br><u>Nodes:</u> For areas of nodal ECS, the whole level should be included. If a node with ECS is present at a border between two lymph node levels, the level above/below should also be included as appropriate. For nodal levels included in the CTV_6500, the overlying sternocleidomastoid muscle (SCM) should be included.	
**CTV_6000**	Include:<br>1. The operative bed if there is no positive margin, as defined above.<br>2. Dissected lymph node levels in which there is no ECS if that side of the neck is pN+. The whole level should be included. The SCM should not be included.	
**CTV_5400**	IA	Included if tumour involves floor of mouth or anterior mandible
	IB	Always included bilaterally if not already included in CTV_6500 or CTV_6000
	II	Always included bilaterally if not already included in CTV_6500 or CTV_6000. If ipsilateral neck is node negative, superior border lies at the inferior border of the transverse process of C1. If ipsilateral neck is node positive, level II is extended superiorly to skull base.
		Always included bilaterally.
	III	Included bilaterally if the oropharynx or anterior tongue are involved
	IV	Included if ipsilateral level IV is node positive
		Not included
	V	Consider including if retromolar trigone primary
	VI	
	RP*	Retropharyngeal (RP)

* In the WISTERIA trial, we recommend that the cranial border of the retropharyngeal nodal level is defined as the upper edge of the body of C1 or the upper extent of the hard palate, whichever is more cranial.

Please note: Laterality or otherwise the tumour should be discussed with the surgeon, radiologist and pathologist and confirmed at MDT.

Appendix 7: Group A data collection schedule

	Pre-diagnosis	Baseline within 42 days of trial entry	Baseline within 21 days of trial entry	Baseline within 14 days of trial entry	Trial Entry	Week 1			Week 2			End of Treatment visit (within 7 days of D10)	4 Week Follow Up visit +/- 3 days	12 Week Follow Up visit (from start of treatment) +/- 3 days
						D1[#*]	D2	D3	D8[#]	D9	D10			
Informed consent – optional tumour sample	x													
Optional tumour sample during diagnostic biopsy	x													
Diagnostic CT scan[a]		x												
Informed consent[b]		x												
ECHO/MUGA[c]			x											
Audiogram[d]			x			As clinically indicated								
Medical history[e]				x										
Physical exam[f]				x		x			x					
Weight (kg) and BSA[g]				x		x			x					
Height (cm)				x										
Vital signs[h]				x		x			x					
ECOG status				x		x			x					
ECG[i]				x		x			x			x		
Pregnancy test[j]				x										
Biochemistry[k]				x		x			x			x	x	x
Haematology[l]				x		x			x			x	x	x
Isotopic GFR[m]				x					x					
Adverse events				x		x			x			x	x	x
Concomitant medications[n]				x		x			x			x	x	x
Patient diary						x			x					
Cisplatin administration									x					
AZD1775 administration[o]						AM & PM doses			AM & PM doses					
Surgery[p]						Within 42 days of first date of treatment								
Blood samples[q]						x						x		x
Pharmacokinetic samples[r]								x			x			
Mandatory tumour biopsy[s]								x				At time of Resection		
Collection of FFPE tumour samples														x
Review of surgical complications														x
Disease free survival[t]						On-going								

Group A data collection schedule notes

**#**	Day 1 and Day 8 should not occur on a Monday.
*****	Day 1 should be within 6 weeks of informed consent.
**a**	Diagnostic Computerised Tomography (CT) with contrast – neck and chest. To be performed within 42 days of trial entry.
**b**	Informed consent must be obtained before any trial procedures occur.
**c**	If significant cardiac history patient required to have ECHO. MUGA can be performed if ECHO is equivocal. To be performed within 21 days of start of trial entry.
**d**	Air and bone conduction audiogram. To be performed within 21 days of trial entry.
**e**	Medical history – comprehensive medical history, demographics, prior treatment.
**f**	Physical examination includes complete review of systems and physical examination of pertinent organ systems and neurological assessment.
**g**	During trial treatment, weight can be performed within 72 hours pre-dose of AZD1775.
**h**	Blood pressure, pulse measurement, temperature and respiratory rate to be performed with the patient sitting for 5 minutes prior to the evaluation.
**i**	Three ECGs, 2-5 minutes apart, to be performed at screening only. At all other time points a single ECG recording should be performed at least 48h pre-dose of AZD1775.
**j**	Women of childbearing potential will require a negative pregnancy test (serum or urine) prior to trial entry.
**k**	Blood samples for sodium, potassium, magnesium, urea, creatinine, calcium, phosphate, albumin, total protein, bilirubin, alkaline phosphatase, AST and/or ALT.<br>Results of blood samples collected at screening visit can be used for Day 1 eligibility if within 72 hours of first dose of AZD1775. During trial treatment, blood samples can be collected within 72 hours **pre-dose** of AZD1775.
**l**	Haematology – Full Blood Count. Results of haematology samples collected at screening visit can be used for Day 1 eligibility if within 72 hours of first dose of AZD1775. During trial treatment, haematology samples can be collected within 72 hours **pre-dose** of AZD1775.
**m**	If creatinine clearance at screening is ≤ 60 ml/min then an isotopic GFR may be carried out and must be > 60 ml/min. If GFR, calculated immediately before any administration of concomitant chemotherapy, is ≤ 60 ml/min then an isotopic GFR may be carried out and must be > 60 ml/min.
**n**	Concomitant medication to be recorded starting on the date of signing of informed consent, throughout the trial.
**o**	Morning dose to be administered prior to cisplatin on Day 8.
**p**	Surgery must be performed within 42 days of first date of trial treatment but at least 6 days after the last cisplatin dose.
**q**	Blood samples will be collected **pre-dose** on Day 1, at the End of Treatment Visit and at the 12 Week Follow Up Visit. Refer to WISTERIA Laboratory Manual.
**r**	Pharmacokinetic samples to be collected before and 2-5 hours after fifth dose of AZD1775 (before the sixth dose) on Day 3 and Day 10. Refer to WISTERIA Laboratory Manual.
**s**	Tumour biopsy to be collected 2-5 hours after fifth dose of AZD1775 (before the sixth dose) on Day 3 and a biopsy to be taken at time of Resection.. To be taken under local anaesthetic (includes biopsy of lymph nodes taken using ultrasound guided biopsy). Time from biopsy taken to fixing in formalin of the biopsy is limited to 30 minutes. Refer to WISTERIA Laboratory Manual.
**t**	Patients to be followed up for 12 months. Notification required if a patient relapses or dies.

Appendix 8: Group B data collection schedule

	Baseline within 21 days of trial entry	Baseline within 14 days of trial entry		Week 1 – Week 2				Week 3		Week 4 – Week 5				Week 6	Week 7 End of Treatment	Weeks 8-11 Weekly Toxicity Assessments #	Week 12 Follow Up visit	FUP 6 and 12 months from start of treatment
				D1* ~	D2	D3	D4	D1	D2	D1	D2	D3	D4	D1				
Informed consent[a]	x		Trial Entry															
ECHO/MUGA[b]	x																	
Planning CT scan[c]	x																	
Audiogram[d]	x			As clinically indicated														
Medical history[e]		x																
Physical exam[f]		x		x				x		x				x				
Weight (kg) and BSA[g]		x		x				x		x				x				
Height (cm)		x																
Vital signs[h]		x		x				x		x				x				
ECOG status		x		x				x		x				x				
ECG[i]		x		x				x		x				x	x			
Pregnancy test[j]		x																
Biochemistry[k]		x		x				x		x				x	x	x	x	x
Haematology[l]		x		x				x		x				x	x	x	x	x
Isotopic GFR[m]		x		x				x		x								
Adverse events		x		x				x		x				x	x	x	x	x
Concomitant medications[n]		x		x				x		x				x	x	x	x	
Radiotherapy[o]				To start within 3 months of surgery														
AZD1775 administration[p]				N/A	AM & PM Doses			N/A			AM & PM Doses			N/A				
Cisplatin administration[q]					x				x		x			N/A				
Patient diary					x						x							
Quality of Life Questionnaires[r]				x											x		x	x
Swallowing Assessment Questionnaire[r]				x											x		x	x
Blood samples[s]				x											x		x	x
Pharmacokinetic sample[t]							x											
Collection of FFPE tumour samples																	x	
Disease free survival[u]				On-going														

Group B data collection schedule notes:

**#**	Patients will undergo weekly toxicity assessment during treatment and weekly thereafter until all acute toxicities have resolved to Grade 1 or less; typically for 42-56 days (6-8 weeks), and for no less than 42 days after completion of treatment, giving a total Dose Limiting Toxicity reporting period of 84 days (12 weeks) from start of treatment.
*****	Day 1 of week 1 should be within 4 weeks of consent.
**~**	Week 1 – Day 1 starts on the first day of radiotherapy.
**a**	Informed consent must be obtained before any trial procedures occur.
**b**	If significant cardiac history patient required to have ECHO. MUGA can be performed if ECHO is equivocal. To be performed within 21 days of start of trial entry.
**c**	CT with contrast – neck as part of radiotherapy preparation and planning at Baseline only. To be performed within 21 days of start of trial entry.
**d**	Air and bone conduction audiogram. To be performed within 21 days of trial entry.
**e**	Medical history – comprehensive medical history, demographics, prior treatment.
**f**	Physical examination includes complete review of systems and physical examination of pertinent organ systems and neurological assessment.
**g**	During trial treatment, weight can be performed within 72 hours pre-dose of AZD1775.
**h**	Blood pressure, pulse measurement, temperature and respiratory rate to be performed with the patient sitting for 5 minutes prior to the evaluation.
**i**	Three ECGs, 2-5 minutes apart, to be performed at baseline only. At all other time points a single ECG recording should be performed at least 48 hours **pre-dose** of AZD1775.
**j**	Women of childbearing potential will require a negative pregnancy test (serum or urine) prior to trial entry.
**k**	Blood samples for sodium, potassium, magnesium, urea, creatinine, calcium, phosphate, albumin, total protein, bilirubin, alkaline phosphatase, AST and/or ALT.<br>Results of blood samples collected at screening visit can be used for Day 1 eligibility if within 72 hours of first dose of AZD1775. During trial treatment, blood samples can be collected within 72 hours **pre-dose** of AZD1775.
**l**	Haematology – Full Blood Count. Results of haematology samples collected at screening visit can be used for Day 1 eligibility if within 72 hours of first dose of AZD1775. During trial treatment, haematology samples can be collected within 72 hours **pre-dose** of AZD1775.
**m**	If creatinine clearance at screening is ≤ 60 ml/min then an isotopic GFR may be carried out and must be > 60 ml/min. If GFR, calculated immediately before any administration of concomitant chemotherapy, is ≤ 60 ml/min then an isotopic GFR may be carried out and must be > 60 ml/min.
**n**	Concomitant medication to be recorded starting on the date of signing of informed consent, throughout the trial.
**o**	Radiotherapy to start on Week 1 - Day 1. Refer to the WISTERIA Radiotherapy Guidelines.
**p**	AZD1775 morning dose to be administered prior to cisplatin and at least 1 hour prior to radiotherapy.
**q**	Cisplatin to be administered after morning dose of AZD1775 and at least 1 hour prior to radiotherapy.
**r**	Questionnaires to be completed by patient in clinic at defined visits: **pre-dose** on Week 1 - Day 1, End of Treatment Visit, Week 12 Follow Up Visit, 6 Month Follow Up Visit and 12 Month Follow Up Visit.
**s**	Blood samples will be collected **pre-dose** on Week 1 - Day 1, at the End of Treatment Visit, the Week 12 Follow Up Visit, the 6 Month Follow Up Visit and the 12 Month Follow Up Visit. Refer to WISTERIA Laboratory Manual.
**t**	Pharmacokinetic sample to be collected before and 2-5 hours after fifth dose of AZD1775 (before the sixth dose) on Week 1 - Day 4 **only**. Refer to WISTERIA Laboratory Manual.
**u**	Patients to be followed up for 12 months. Notification required if a patient relapses or dies.

Appendix 9: Toxicity Profiles

Treatment	Very common side effects (1 patient in 10)	Common side effects (1 patient in 100)	Other side effects
AZD1775	• Blood & lymphatic disorders including anaemia & thrombocytopenia<br>• Decreased appetite<br>• Gastrointestinal disorders including diarrhoea, nausea, vomiting & serum electrolyte decreases<br>• Dyspepsia	• Neutropenia	• QTc prolongation (rare, affecting 1 patient in 10,000)
AZD1775 in combination with chemotherapy	• Asthensia<br>• Leukopenia<br>• Loss of strength & weakness	• Fatigue<br>• Febrile neutropenia<br>• Mucosal inflammation<br>• Myalgia<br>• Stomatitis	• Constipation<br>• Gastrointestinal haemorrhage<br>• Lymphopenia<br>• Lymphocyte count decrease<br>• Pancytopenia<br>• Sepsis<br>• Tachycardia
Cisplatin	• Blood & lymphatic disorders including bone marrow failure, thrombocytopenia, leukopenia & anaemia<br>• Metabolism & nutrition disorders including dehydration<br>• Renal & urinary disorders including hyperuricaemia	• Sepsis<br>• Ear disorders including ototoxicity<br>• Cardia disorders including arrhythmia, bradycardia & tachycardia<br>• Inflammation at injection site<br>• Respiratory disorders including dyspnoea & pneumonia	
Radiotherapy	• Tiredness<br>• Pain & difficulty swallowing due to mouth ulceration<br>• Thickened saliva & secretions<br>• Dry mouth<br>• Altered sense of taste<br>• Nausea<br>• Skin soreness & ulceration<br>• Hair loss to are near to radiotherapy site		• Due to swallowing difficulties there is an increased risk of chest infection (pneumonia) during treatment

Appendix 10: Haematological Toxicity Dose Management – ANC, Platelets

Symptoms: ANC		Symptoms: Platelets	Action
≥1500/10^9/L	And	≥75000/10^9/L	No cisplatin or AZD1775 dose modification or interruption.
<1500/10^9/L	Or	<75000/10^9/L	Delay cisplatin and AZD1775 by 1 week intervals until recovery. If DLT is reached, discontinue AZD1775 treatment but cisplatin may be resumed if deemed appropriate by the investigators.

Appendix 11: Haematological Toxicity Dose Management – Neutropenia, Infection, Febrile Neutropenia

Symptoms	Action
Grade 3 febrile neutropenia (ANC <1000/10^9/L + Temperature ≥38°C) or neutropenic sepsis<br>Grade 4 neutropenia (ANC <500/10^9/L >7 days)<br>Grade 4 thrombocytopenia (platelet count <25,000/10^9/L >7 days)	Hold cisplatin and AZD1775 dose until recovery. If DLT is reached, discontinue AZD1775 treatment but cisplatin may be resumed if deemed appropriate by the investigators.
Grade 4 febrile neutropenia or Grade 4 infection with neutropenia (both defined as septic shock)<br>Thrombocytopenic haemorrhage (gross occult bleeding) associated with a platelet count <50,000/10^9/L	Discontinue cisplatin and AZD1775 treatment.
Febrile neutropenia (≥ 38° C) with or without significant symptoms	Patient to be managed in a hospital setting according to standard procedures, with the urgent initiation of IV antibiotic therapy according to local guidelines.

Appendix 12: Non-Haematological Toxicity Dose Management – General Disorders

CTCAE v4.03	Cisplatin	AZD1775
**Grade 0-2**	No dose modification	No dose modification
**Grade 3**	Hold until toxicity resolves to ≤ Grade 1, and then resume at the same dose with no modification if no DLT is reached and if deemed appropriate by the investigators.	Hold until toxicity resolves to ≤ Grade 1, and then resume at the same dose with no modification. Discontinue treatment if DLT is reached
**Grade 4 toxicity** (except anorexia)	Discontinue treatment	Discontinue treatment

Appendix 13: Non-Haematological Toxicity Dose Management – Hepatic

CTCAE v4.03	Cisplatin	AZD1775
**Grade 1-2**	No dose modification	No dose modification
**Grade 3** (manifested as elevations in ALT, AST, ALP or bilirubin)	Hold until resolves to Grade ≤1 or baseline, then resume cisplatin with no dose modification if no DLT is reached and if deemed appropriate by the investigators.	Hold until resolves to Grade ≤1 or baseline, then resume study drug with no dose reduction. If not resolved within 28 days discontinue study drug. Discontinue treatment if DLT is reached.
**Grade 4** (Life threatening)	Discontinue treatment	Discontinue treatment

Appendix 14: Non-Haematological Toxicity Dose Management – Diarrhoea or Mucositis

CTCAE v4.03	Cisplatin	AZD1775
**Grade 3** (requiring hospitalisation)	Hold until toxicity resolves to ≤ Grade 1, and then resume at the same dose with no modification if no DLT is reached and if deemed appropriate by the investigators.	Hold until toxicity resolves to ≤ Grade 1, and then resume at the same dose with no modification. Discontinue treatment if DLT reached.
**Grade 4**	Discontinue treatment	Discontinue treatment

Appendix 15: Non-Haematological Toxicity Dose Management – Renal Toxicity

CTCAE v4.03	Cisplatin	AZD1775
**Grade ≥ 2**	Hold until resolves to Grade ≤1 or baseline. If GFR falls below 60 mL/min, cisplatin should be discontinued and consideration given to substitution with carboplatin AUC =1.5, according to routine local practice.	Hold until toxicity resolves to ≤ Grade 1, and then resume at the same dose with no modification. Discontinue treatment if DLT reached

Appendix 16: Non-Haematological Toxicity Dose Management – Neurotoxocity

CTCAE v4.03	Cisplatin	AZD1775
**Grade 1**	No dose modification	No dose modification
**Grade 2**	Hold until toxicity resolves to Grade ≤1. Resume with 1 dose level reduction.	No dose modification
**Grade 3 or 4**	Discontinue treatment	Discontinue treatment

Appendix 17: Disallowed medications and medication to be administered with caution

A list of the main CYP3A4 substrates, inhibitors (strong and moderate) and inducers, CYP2C19 substrates, P-gp substrates and inhibitors and BCRP substrates are shown below:
Please note: Live vaccines are not permitted

This is not an exhaustive list and further details can be found at:
www.fda.gov/drugs/developmentapprovalprocess/developmentresources/druginteractionslabeling/ucm093664.htm

CYP3A4 Inhibitors (Strong)

Boceprevir
Clarithromycin
Cobicistat (GS-9350)
Conivaptan
Danoprevir
Elvitegravir
Fosamprenavir
Grapefruit juice
Idelalisib
Indinavir
Itraconazole
Ketoconazole
LCL161
Lopinavir
Mibefradil
Nefazodone
Nelfinavir
Posaconazole
Ritonavir
Saquinavir
Telaprevir
Telithromycin
Tipranavir
Troleandomycin
Voriconazole

CYP3A4 Inhibitors (Moderate)

ACT-178882	Darunavir	Imatinib
Amprenavir	Dronedarone	Ledipasvir
Aprepitant	Diltiazem	Lomitapide
Atazanavir	Erythromycin	Netupitant
Casopitant	FK1706	Schisandra sphenanthera
Ciprofloxacin	Fluconazole	Tofisopam
Crizotinib	Fosamprenavir	Verapamil

CYP3A4 Inhibitors (Weak)

Almorexant	Everolimus	Propiverine
Alprazolam	Faldaprevir	Ranitidine
AMD070	Fluvoxamine	Ranolazine
Amiodarone	Fosaprepitant (IV)	Resveratrol
Amlodipine	Ginkgo	Roxithromycin
Atorvastatin	Goldenseal	Seville orange juice
Azithromycin	GSK1292263	Simeprevir
Berberine	GSK2248761	Sitaxentan
Bicalutamide	Isoniazid	Suvorexant
Blueberry juice	Ivacaftor	Tabimorelin
Chlorzoxazone	Lacidipine	Tacrolimus
Cilostazol	I Linagliptin	Teriflunomide
Cimetidine	Lomitapide	Ticagrelor
Clotrimazole	M100240	Tipranavir/ritonavir
Cranberry juice	Nilotinib	Tolvaptan
Cyclosporine	Oral contraceptives	Zileuton
Daclatasvir	Pazopanib	
Delavirdine	Peppermint oil	

CYP3A4 Inducers (Strong and Moderate)

Avasimibe	Lersivirine	Rifabutin
Bosentan	Lopinavir	Rifampin
Carbamazepine	Mitotane	Ritonavir
Efavirenz	Modafinil	Semagacestat
Enzalutamide	Nafcillin	St John's Wort
Etravirine	Phenobarbital	Thioridazine
Genistein	Phenytoin	Tipranavir

CYP3A4 Inducers (Weak)

Amprenavir	Echinacea	Oxcarbazepine
Aprepitant	Eslicarbazepine	PA-824
Armodafinil	Garlic	Pleconaril
AZD 7325	Gingko	Prednisone
Bexarotene	Ginseng	Quercetin
Boceprevir	Glycyrrhizin	Raltegravir
Brivaracetam	LCL161	Ritonavir
Clobazam	Methylprednisolone	Rufinamide
Danshen	Nevirapine	Sorafenib
Dexamethasone	Oritavancin	Stribild

Telaprevir
Terbinafine
Ticagrelor
Ticlopidine
Topiramate
Troglitazone
Vemurafenib
Vicriviroc and ritonavir
Vinblastine

CYP3A and CYP3A4 Sensitive Substrates or Substrates with a Narrow Therapeutic Range

ABT-384
Alfentanil
Aprepitant
Alfuzosin
Almorexant
Alpha-Dihydroergocryptine
Amiodarone
Aplaviroc
Aprepitant
Astemizole
Atazanavir
Atorvastatin
Avanafil
Bexarotine
BIRL 355
Bortezomib
Bosutinib
Brecanavir
Brotizolam
Budesonide
Buspirone
Capravirine
Carbamazepine
Casopitant
Cisapride,
Conivaptan
Cyclophosphamide
Cyclosporine
Danoprevir
Darifenacin
Darunavir
Dasatinib
Dihydroergotamine
Disopyramide
Dronedarone
Docetaxol
Dofetilide
Doxorubicin
Ebastine
Eletriptan
Elvitegravir
Eplerenone
Ergotamine
Erlotinib
Etoposide
Everolimus
Felodipine
Fentanyl
Fluticasone
Gefitinib
Halofantrine
Ibrutinib
Ifosfamide
Imatinib
Indinavir
Ironotecan
Ivacaftor
Ixabepilone
L-771,688
Lapatinib
Levomethadyl (LAAm)
Lomitapide
Lopinavir
Lovastatin
Lurasidone
Maraviroc,
Midazolam
Midostaurin
Mosapride
Neratinib
Nilotinib
Nisoldipine
Paclitaxel
Pazopanib
Perospirone
Pimozide
Propafenone
Propofol
Quetiapine
Quinidine
Ranolazine
Ridaforolimus
Romidepsin
Saquinavir
Sildenafil
Simeprevir
Simvastatin
Sirolimus
Tacrolimus
Temsirolimus
Terfenadine
Ticagrelor
Theoophylline
Thioridazine
Thiotepa
Tilidine
Tipranavir
Tolvaptan
Triazolam
Tretinoin
Ulipristal
Vardenafil
Vicriviroc
Voclosporin

CYP2C19 Sensitive Substrates or Substrates with a Narrow Therapeutic Range

Diazepam
Gliclazide
Lansoprazole
(R)-Lansoprazole
(S)-Lansoprazole
(S)-Mephenytoin
(R)-Mephobarbital
Omeprazole
(R)-Omeprazole
Pantoprazole
(+)-Pantoprazole
Rabeprazole
Tilidine

CYP1A2 Sensitive Substrates or Substrates with a Narrow Therapeutic Range

Alosetron	Melatonin	Theophylline
Caffeine	Ramelteon	Tizanidine
Duloxetine	Tacrine	

P-gp Substrates

Colchicine	Indinavir	Vincristine
Digoxin	Paclitaxel	
Fexofenadine	Toptecan	

If a patient requires initiation of digoxin during the study, or is already receiving treatment with digoxin, monitoring of digoxin levels is recommended according to local practice (as the levels of digoxin may increase). Monitoring of digoxin levels is also recommended when the patient has completed dosing with study treatment (as the levels of digoxin may then decrease).

P-gp Inhibitors (Strong)

Cyclosporine
Elacridar
Erythromycin
Itraconazole
Ketocoanzole
LY335979Quinidine
Ritonavir
Valspodar
Verapamil

BCRP Substrates

Daunorubicin
Doxorubicin
Rosuvastatin
Sulfasalazine
Topotecan
