# Supplementary material for: Phase I trial of WEE1 inhibition with chemotherapy and radiotherapy as adjuvant treatment, and a window of opportunity trial with cisplatin in patients with head and neck cancer: the WISTERIA trial protocol
Source: BMJ Open. 2020 Mar 16;10(3):e033009. doi: 10.1136/bmjopen-2019-033009 (PMC7076237; doi:10.1136/bmjopen-2019-033009)
Supplement: Author's manuscript [file bmjopen-2019-033009.draft_revisions.pdf]

BMJ Open

BMJ Open is committed to open peer review. As part of this commitment we make the peer review history of every article we publish publicly available.

When an article is published we post the peer reviewers' comments and the authors' responses online. We also post the versions of the paper that were used during peer review. These are the versions that the peer review comments apply to.

The versions of the paper that follow are the versions that were submitted during the peer review process. They are not the versions of record or the final published versions. They should not be cited or distributed as the published version of this manuscript.

BMJ Open is an open access journal and the full, final, typeset and author-corrected version of record of the manuscript is available on our site with no access controls, subscription charges or pay-per-view fees (http://bmjopen.bmj.com).

If you have any questions on BMJ Open's open peer review process please email info.bmjopen@bmj.com

BMJ Open

A Phase I trial of WEE1 inhibition with Chemotherapy and Radiotherapy as adjuvant treatment, and a Window of Opportunity trial with Cisplatin in Patients with Head and Neck Cancer: The WISTERIA Trial

Journal:	*BMJ Open*
Manuscript ID	bmjopen-2019-033009
Article Type:	Protocol
Date Submitted by the Author:	16-Jul-2019

Complete List of Authors:	Kong, Anthony; University of Birmingham, Institute of Cancer and Genomic Sciences<br>Good, James; University Hospitals Birmingham NHS Foundation Trust<br>Kirkham, Amanda; University of Birmingham, Cancer Research UK Clinical Trials Unit, Institute of Cancer and Genomic Sciences<br>Savage, Joshua; University of Birmingham, Cancer Research UK Clinical Trials Unit, Institute of Cancer and Genomic Sciences<br>Mant, Rhys; University of Birmingham, Cancer Research UK Clinical Trials Unit, Institute of Cancer and Genomic Sciences<br>Llewellyn, Laura; SIMBEC Research Limited<br>Parish, Joanna; University of Birmingham, Institute of Cancer and Genomic Sciences<br>Spruce, Rachel; University of Birmingham, InHANSE, Institute of Cancer and Genomic Sciences<br>Forster, Martin; University College London<br>Schipani, Stefano; University of Glasgow, Beatson West of Scotland Cancer Centre<br>Harrington, Kevin; Institute of Cancer Research<br>Sacco, Joseph; Clatterbridge Cancer Centre NHS Foundation Trust<br>Murray, Patrick; Leeds Teaching Hospitals NHS Trust<br>Middleton, Gary; University of Birmingham, Institute of Immunology and Immunotherapy<br>Yap, Christina; University of Birmingham, Cancer Research UK Clinical Trials Unit, Institute of Cancer and Genomic Sciences<br>Mehanna, H; University of Birmingham, InHANSE, Institute of Cancer and Genomic Sciences

Keywords:	head and neck squamous cell carcinoma, TITE-CRM, wee1 inhibitor, chemoradiation, window of opportunity study, wisteria

SCHOLARONE™
Manuscripts

I, the Submitting Author has the right to grant and does grant on behalf of all authors of the Work (as defined in the below author licence), an exclusive licence and/or a non-exclusive licence for contributions from authors who are: i) UK Crown employees; ii) where BMJ has agreed a CC-BY licence shall apply, and/or iii) in accordance with the terms applicable for US Federal Government officers or employees acting as part of their official duties; on a worldwide, perpetual, irrevocable, royalty-free basis to BMJ Publishing Group Ltd ("BMJ") its licensees and where the relevant Journal is co-owned by BMJ to the co-owners of the Journal, to publish the Work in this journal and any other BMJ products and to exploit all rights, as set out in our licence.

The Submitting Author accepts and understands that any supply made under these terms is made by BMJ to the Submitting Author unless you are acting as an employee on behalf of your employer or a postgraduate student of an affiliated institution which is paying any applicable article publishing charge ("APC") for Open Access articles. Where the Submitting Author wishes to make the Work available on an Open Access basis (and intends to pay the relevant APC), the terms of reuse of such Open Access shall be governed by a Creative Commons licence – details of these licences and which Creative Commons licence will apply to this Work are set out in our licence referred to above.

Other than as permitted in any relevant BMJ Author's Self Archiving Policies, I confirm this Work has not been accepted for publication elsewhere, is not being considered for publication elsewhere and does not duplicate material already published. I confirm all authors consent to publication of this Work and authorise the granting of this licence.

For peer review only

A Phase I trial of WEE1 inhibition with Chemotherapy and Radiotherapy as adjuvant treatment, and a Window of Opportunity trial with Cisplatin in Patients with Head and Neck Cancer: The **WISTERIA** Trial

AUTHORS

Kong A*[1], Good J*[2], Kirkham AJ[3] , Savage J[3], Mant R[3], Llewellyn L[8], Parish J[1], Spruce R[1], Forster M[4], Schipani S[5], Harrington K[6], Sacco JJ[7], Murray P[10], Middleton G [9], Yap C[‡3], Mehanna H[‡1†]

*Joint first authors
‡Joint senior authors
† Corresponding author

Corresponding author:
Professor Hisham Mehanna
InHANSE, Institute of Cancer and Genomic Sciences
University of Birmingham
Edgbaston
Birmingham
B15 2TT
H.Mehanna@bham.ac.uk
0121 414 6547

Author Affiliations

[1] Institute of Cancer and Genomic Sciences, University of Birmingham, Edgbaston, Birmingham, UK
[2] University Hospitals Birmingham NHS Foundation Trust, Queen Elizabeth Hospital Birmingham, Mindelsohn Way, Edgbaston, Birmingham, UK
[3] Cancer Research UK Clinical Trials Unit, Institute of Cancer and Genomics Sciences, University of Birmingham, Edgbaston, Birmingham, UK
[4] University College Hospital London, Fitzrovia, London, UK
[5] Beatson West of Scotland Cancer Centre, Institute of Cancer Sciences, University of Glasgow, UK
[6] Royal Marsden/The Institute of Cancer Research,NIHR Biomedical Research Centre, London, UK
[7] The Clatterbridge Cancer Centre, Wirral/University of Liverpool, Liverpool, UK
[8] Simbec Research Ltd, Simbec Research Campus, Pentrebach, Merthyr Tydfil, South Wales, UK
[9] Institute of Immunology and Immunotherapy, University of Birmingham, Edgbaston, Birmingham, UK
[10] St James' University Hospital, Leeds Teaching Hospitals NHS Trust

Keywords (5 max): TITE-CRM, head and neck squamous cell carcinoma, wee1 inhibitor, chemoradiation, window of opportunity study

Word count excluding abstract, figures, tables, boxes and appendices: 4405

For peer review only

ABSTRACT

Introduction

Head and neck squamous cell carcinoma (HNSCC) patients with locally advanced disease often require multimodality treatment with surgery, radiotherapy and/or chemotherapy. Adjuvant radiotherapy with concurrent chemotherapy is offered to patients with high-risk pathological features post-surgery. Whilst cure rates are improved, overall survival remains sub-optimal and treatment has a significant negative impact on quality of life.

Cell cycle checkpoint kinase inhibition is a promising method to selectively potentiate the therapeutic effects of chemo-radiation. Our hypothesis is that combining chemo-radiation with a WEE1 inhibitor will affect the biological response to DNA damage caused by cisplatin and radiation, thereby enhancing clinical outcomes, without increased toxicity. This trial explores the associated effect of WEE1 kinase inhibitor Adavosertib (AZD1775).

Methods and analysis

This Phase I dose-finding, open-label, multicentre trial aims to determine the highest safe dose of AZD1775 in combination with cisplatin chemotherapy pre-operatively (Group A) as a window of opportunity trial; and in combination with post-operative cisplatin-based chemo-radiation (Group B). Modified time-to-event continual reassessment method (TITE-CRM) will determine the recommended dose, recruiting upto 21 patients per group. Primary outcomes are recommended doses with predefined target dose-limiting toxicity probabilities of 25% monitored up to 42 days (Group A), and 30% monitored up to 12 weeks (Group B). Secondary outcomes are disease-free survival times (Groups A and B). Exploratory objectives are evaluation of pharmacodynamic (PD) effects, identification and correlation of potential biomarkers with PD markers of DNA damage, determine rate of resection status and surgical complications for Group A; and quality of life in Group B.

Ethics and dissemination

Research Ethics Committee, Edgbaston, West Midlands (REC, reference 16/WM/0501) initial approval received on 18/01/2017. Results will be disseminated via peer reviewed publication and presentation at international conferences.

Trial Registration

European Medicines Agency (EudraCT: 2015-003583-37); ISRCTN is 76291951 (http://www.isrctn.com/ISRCTN76291951); Clinicaltrials.gov identifier is NCT03028766 (http://clinicaltrials.gov/ct2/show/NCT03028766).

For peer review only

BOX 1: Strengths and Limitations

Strengths of the study:

- The study is one of the first to explore a WEE1 inhibitor in combination with cisplatin and radiotherapy, and the first to do so in head and neck cancer
- Allows collection of tissues for translational research to examine the pharmacodynamic effects of WEE1 and WEE1 in combination with cisplatin
- Uses an efficient TITE-CRM design which allows dose assignments to be performed with the flexibility of continual patient accrual or temporary accrual suspension to permit sufficient accumulation of safety information as necessary. It uses both partial and complete DLT information of all accumulated patients and dose information to inform dose recommendation

Limitations of the study:

- The window of opportunity study utilises a single dose of WEE1 followed by a separate single dose of cisplatin due to the short timescale between diagnosis and standard treatment
- Additional biopsies in the pre-operative setting may affect the patient enrolment
- The oral administration of AZD1775 may affect recruitment in post-operative patients

For peer review only

INTRODUCTION

Head and neck cancers are the sixth most common cancer worldwide[1], with 12,000 new cases per year in the UK (CRUK head and neck cancer statistics). The most common types are squamous cell carcinoma (HNSCC) arising from laryngeal, oral cavity , oropharynx and hypopharyngeal subsites[2].
Primary surgery is the standard of care for cancers of the oral cavity, and for more advanced cancers of the larynx and hypopharynx, whilst (chemo)-radiation is the standard approach to oropharyngeal disease. Post-operative radiotherapy (PORT), or post-operative chemoradiation (POCRT), is often recommended in the setting of high-risk features such as a positive margin, multiple involved nodes, extra-capsular spread, and peri-neural infiltration.

The role of concomitant chemotherapy was established by the Meta-Analysis of Chemotherapy on Head and Neck Cancer (MACH-NC) in 2009[2]. This study pooled individual patient data from 93 trials involving 16,485 patients receiving either definitive local therapy alone (surgery or radiotherapy) or local therapy plus chemotherapy (induction, concomitant or adjuvant). Concomitant therapy was assessed in 50 trials involving 9605 patients; chemotherapy decreased the risk of death with a hazard ratio of 0.81, translating into a 6.5% overall survival advantage at 5 years as a result of improved disease-free survival. No benefit was seen in patients aged over 70 years. This provides robust evidence that the ability of radiotherapy to secure local control can be enhanced by systemic therapy.

Acute toxicity and long-term sequelae of POCRT have a considerable negative health impact, and despite intensive treatment, five year overall survival is sub-optimal at 53%[3]. Loco-regional relapse is particularly difficult to salvage, and local control is therefore closely correlated with overall survival. Morbidity imposed by local recurrence in the absence of distant metastatic disease is considerable and can be extremely challenging to palliate adequately. There is an urgent need to develop novel approaches that achieve improved loco-regional disease control and reduce treatment-related morbidity for this patient group. Achieving this may translate into improved overall survival and an enhancement in patient-related outcome measures.

Chemotherapy, radiotherapy and DNA damage response

Cellular DNA damage response (DDR) is central to the preservation of genomic integrity. Cancer development is a multistep process where deregulation of the DDR contributes to phenotypes such as sustained cell proliferation and resistance to cell death. This increase in endogenous DNA damage necessitates the acquisition of compensatory mechanisms if the cell is to avoid death through uncontrolled genomic instability. Importantly, these compensatory changes may constitute a molecular 'Achilles heel' that is vulnerable to therapeutic exploitation with a new generation of targeted agents. POCRT exploits the differential DDR in malignant and normal tissues to eradicate microscopic residual disease. Ionising radiation (IR) generates a variety of biological changes within irradiated tissue, with double-stranded DNA (dsDNA) breaks being therapeutically significant (figure 1). IR-induced cell death is also immunogenic, with recruitment of immune effectors to the irradiated tumour micro-environment contributing to a successful outcome[4]. Platinum-based chemotherapy accentuates IR-induced cell death, in part via the suppression of homologous recombination (HR), the primary repair mechanism for dsDNA damage in irradiated cells[4]. Manipulation of the DDR pathway therefore represents a rational means by which the therapeutic index of POCRT might be improved. Cell cycle checkpoints are integral components of the DDR, allowing the cell to pause progression through the cell cycle to repair DNA damage. Checkpoints are regulated by a network of phosphorylation cascades. *p53*, encoded by *TP53*, is a key regulator of the

For peer review only - http://bmjopen.bmj.com/site/about/guidelines.xhtml

G_1/S checkpoint. *TP53* mutations, which are seen in 60-70% of HNSCC cases[5], are sufficient to impair the function of this checkpoint and thereby create a critical reliance on the later G_2/M checkpoint. In addition, p53 function can be inactivated by various mechanisms, including somatic and germline mutations as well as polymorphisms[6,7].

Pharmacological abrogation of the G_2/M checkpoint has been shown to differentially sensitise normal and tumour cells to the effect of DNA damaging agents such as cisplatin and IR[8].

WEE1 kinase and AZD1775

WEE1 kinase is a key regulator of the G_2/M checkpoint and a promising therapeutic target. It is a serine-threonine kinase involved in phosphorylation and inactivation of cyclin-dependent kinase 1 (CDK1), the only one of 14 similar proteins to be indispensable for mitotic entry. Along with a number of other proteins, WEE1 triggers G_2/M arrest in response to DNA damage. However, inhibition of WEE1 leads to high CDK1 activity, allowing cells to progress through the G_2/M checkpoint without opportunity to repair damaged DNA, leading to potentially catastrophic levels of unrepaired DNA damage induction of cell death[9,10]. WEE1 also has an effect on CDK2 as its inhibition leads to high CDK2 activity and aberrant DNA replication, resulting in stalled replication forks and DNA double-stranded breaks. WEE1 upregulation is seen in a variety of human cancers and is inversely associated with prognosis in some models[11, 12]. Two separate kinomic screens in HNSCC identified WEE1 expression as a particularly strong determinant of cell survival[13, 14], indicating that HNSCC may be a fruitful setting in which to investigate the clinical effects of WEE1 inhibition.

Adavosertib (AZD1775) is a potent, selective small molecule inhibitor of WEE1. It has been shown to potentiate the activity of various chemotherapeutic agents *in vitro* and *in vivo*. Some studies suggest the sensitising effect is only seen in p53-deficient tumours[14-16] although not exclusively[17]. It has also been show to enhance IR-induced cell death in TP53-mutant cell lines.

Co-exposure of AZD1775 and cisplatin were found to reduce clonogenic survival[18], demonstrating this combination therapy has the ability to overcome cisplatin resistance in HNSCC. Similar effects of this compound on radiation-induced cell death have been seen in models of typically radio-resistant cancer such as pontine glioma[19], glioblastoma[20], and pancreatic adenocarcinoma[21]. Importantly, one study has shown that WEE1 inhibition by AZD1775 sensitises acute myelogenous leukaemia and lung cancer cell lines to cytarabine chemotherapy independently of p53 status[22], suggesting that p53 mutation as a predictive biomarker for response to WEE1 inhibition may be cancer and/or chemotherapy specific. WEE1 has also been implicated in maintaining genomic stability through stabilisation of replication forks - down-regulation reduces replication fork speed during S-phase, generating potentially lethal dsDNA breaks[23]. By impacting both cell cycle progression and DNA damage repair, WEE1 inhibition may potentiate cell death in response to chemotherapy and IR. This suggests that there may be an additive effect on clinical outcome in combination with POCRT, as well as potential synergy.

AZD1775 is being tested in many clinical settings including in combination with radiotherapy in childhood pontine glioma (NCT01922076), with temozolomide and radiotherapy in glioblastoma (NCT01849146), and with cisplatin and radiotherapy in cervical cancer (NCT01958658).

In summary, the available mechanistic data lend strong support to combining AZD1775 with cisplatin and with POCRT in the clinic. Given that the predictive effect of TP53 mutation on such combinations has yet to be clinically validated, this study does not prospectively stratify patients based on any

For peer review only - http://bmjopen.bmj.com/site/about/guidelines.xhtml

such clinical or biological characteristic. Biomarker data generated, however, will be important in understanding the mechanism of action and informing future combination studies.

Incorporating AZD1775 into clinical management

A recent randomised phase III study of neo-adjuvant chemotherapy in a Chinese population with locally advanced resectable oral cavity cancer showed that docetaxel, cisplatin and 5-fluorouracil (TPF) treatment generated a response rate of 80%, with an exploratory analysis showing that clinical responders had improved overall survival and local control[24]. There was no improvement in overall survival for the study group as a whole compared to the control group without upfront TPF chemotherapy, possibly because patients who would ordinarily have received POCRT received PORT alone in this study. In an earlier study, TP53 mutation conferred increased resistance to pre-operative cisplatin-based chemotherapy in oral cancer[25]. Whilst neo-adjuvant chemotherapy has yet to find a definitive role prior to surgery, these findings raise the possibility that chemo-sensitisation may further improve outcomes.

The pre-operative Group A sets out to test this hypothesis by combining one week of AZD1775 alone, with a second week of cisplatin (40 mg/m^2) with AZD1775. This short course of treatment will minimise any delay between diagnosis and definitive surgery and prevent unacceptable delay for the non-responders from getting standard treatment. If this is a promising combination, the long-term aim is to test AZD1775 in the neoadjuvant setting to downstage the disease through treatment with cisplatin and AZD1775 in the pre-operative setting, which may decrease the extent of surgery, and may also reduce the need for PORT/POCRT by reducing the involved margin rate. Many patients with tumours that have high-risk histopathological features develop relapsed disease within the anatomical area treated to a high radiation dose. Chemo- and radio-sensitisation via inhibition of the G_2/M checkpoint by AZD1775 has the potential to improve outcomes with acceptable toxicity. This possibility is the basis of the treatment delivered in the Group B cohort.

METHODS

Wisteria trial objectives

Primary objectives are to determine the recommended dose and safety profile of AZD1775 with cisplatin in the Group A pre-operative (window-of-opportunity) setting, and with cisplatin/radiotherapy in the Group B post-operative setting. Other objectives: obtain information regarding Group A and B disease-free survivals; evaluate AZD1775 pharmacodynamic (PD) effects when administered with cisplatin (Group A); identify and correlate potential predictive biomarkers with PD markers of DNA damage (Group A); determine the rate of R0, R1 or R2 resection status and surgical complication (Group A); and Group B quality of life (QoL).

Trial design

Wisteria is an open-label, dose-finding, multicentre Phase I trial to determine the highest safest dose of AZD1775 in combination with a single dose of cisplatin in the pre-operative setting as a window of opportunity trial (Group A); and to determine the maximum tolerated dose (MTD) of AZD1775 in combination with cisplatin/radiotherapy in the post-operative setting (Group B): both groups recruit and run in parallel (Figure 2). The dose-escalation design is based on the modified time to event

For peer review only - http://bmjopen.bmj.com/site/about/guidelines.xhtml

continual reassessment method (TITE-CRM)[26-29], requiring a maximum of 21 patients per group, and encompassing up to four dose levels of AZD1775.

The MTDs of AZD1775 for both groups are expected to differ given the additional toxicities of radiotherapy in Group B. The DLT monitoring periods are defined in table 1. Conservative target dose limiting toxicity (DLT) rates are selected to minimise the likelihood of compromising individual patient's radical surgery and/or post-operative radiotherapy. To maximise recruitment, reduce trial suspension time between cohorts, whilst balancing safety and optimal patient allocation, screening cohorts of up to five patients will be allowed if the dose has previously been tested. Recruited patients will be allocated to the current recommended dose up to a maximum of five. Patients who become unevaluable may be replaced.

The model will be updated after every two to three evaluable patients, with any subsequent eligible patients (not already receiving treatment) allocated to the latest recommended dose cohort. Subsequent cohorts will be assigned a dose level using all the data observed until either - the MTD is determined, the maximum sample size is reached, or the trial is stopped early due to unacceptable DLT levels at the lowest dose.

Patient population, screening and consent

Group A patients are those with biopsy proven squamous cell carcinoma of the oral cavity, larynx or hypopharynx (having accessible tumours for re-biopsy under local anaesthetic or via ultrasound-guided biopsy), and due to undergo surgery. Patients in Group B consist of those already diagnosed with oral cavity, larynx or hypopharynx squamous cell carcinoma, who have undergone surgery and are considered at risk of relapse after surgery (i.e. with positive margins and/or nodal extracapsular spread). Patients with cancer of the oropharynx will not be included. Patients who meet the criteria will be supplied with the Patient Information Sheet. If informed consent is given the patient undergoes a full screening evaluation to ensure they satisfy the eligibility criteria prior to registration (Boxes 2 and 3).

Dose selection

Based on AstraZeneca studies[30, 31], the Wisteria trial uses 3-day dosing, giving AZD1775 weekly to coincide with cisplatin administration thereby potentiating the DNA damage effects. The maximum total dose of AZD1775 1800 mg in combination with 1 dose of 40mg/m^2 cisplatin for Group A is substantially lower than AZD1775 monotherapy dose 2250mg every 21 days (225mg twice per day orally over 2.5 days per week for 2 weeks per 21-day cycle) used in Do *et al* (2015); and although in combination with cisplatin, the highest dose level (Dose Level 2) is likely to be safe.

A similar dose escalation algorithm will be carried out in Group B with a higher acceptable target DLT level of 30% allowing for multiple dosing of cisplatin with radiotherapy in combination with AZD1775[32-34]. Trial doses and schedule are set out in table 1. AZD1775 is administered orally. The feasibility of the oral administration will be evaluated in post-operative patients.

For peer review only - http://bmjopen.bmj.com/site/about/guidelines.xhtml

Table 1: Details of the Wisteria TITE-CRM trial design for Groups A and B

	Group A		Group B		
**Maximum Number of Patients**	Up to 21 patients in each Group				
**MTD Definition**	Dose closest to where 25% patients experience a DLT		Dose closest to where 30% patients experience a DLT		
**Minimum Treatment To Be DLT Evaluable**	Patients to have received the minimum of AZD1775 and Cisplatin doses scheduled up to and including day 8		Patients to have received a minimum of the first two weeks of treatment as scheduled		
**DLT Reporting Period**	Minimum of 30 days from start of treatment up to 42 days for delays in surgery due to treatment related toxicity (if this exceeds 42 days then this delay will be classified as a DLT).		Minimum of 56 days (8 weeks) from start of radiotherapy, and up to 84 days (12 weeks), i.e. up to 6 weeks from end of post-operative chemo-radiation therapy.		
**Dose Levels :**	**Cisplatin (iv) over 1 hr** (Day 8)	**AZD1775 PO BID 3 days** (Days 1-3, 8-10)	**Cisplatin (iv) over 1 hr** (Day 2 weeks 1-5)	**Radiotherapy** (5 days per week over 6 weeks)	**AZD1775 PO BID 3 days** (Days 2-4 weeks 1, 2, 4 & 5)
**-1**	40 mg/m^2	75 mg	40 mg/m^2	54-65 Gy in 30#	50 mg
**0 (starting dose level)**		100 mg			75 mg
**1**		125 mg			100 mg
**2**		150 mg			125 mg

(iv) Intravenous; (PO) By Mouth; (BID) Twice A Day; (Gy) Gray - unit of radiation dose; (#) Daily Fractions

For peer review only

For peer review only - http://bmjopen.bmj.com/site/about/guidelines.xhtml

Box 2: Inclusion criteria – all patients

- Histologically confirmed diagnosis of oral, laryngeal or hypopharyngeal squamous cell carcinoma
- Multi-Disciplinary Team (MDT) recommendation for surgical resection with curative intent
- Eastern Cooperative Oncology Group (ECOG) performance status 0/131
- Age ≥18 to ≤70 years
- Creatinine clearance, measured by Glomerular Filtration Rate (GFR), ≥ 60 ml/min at baseline calculated using local practice calculation. If this is ≤ 60 ml/min then an isotopic GFR may be carried out and must be > 60 ml/min
- Acceptable cardiac function. If significant cardiac history, then required for patient to have Left Ventricular Ejection Fraction (LVEF) ≥55% by echocardiogram (ECHO) or Multiple Gated Acquisition Scan (MUGA, if ECHO is equivocal)
- Normal liver and bone marrow function:
  - Haemoglobin (Hb) ≥10.0 g/dL or ≥100 g/L
  - Absolute neutrophil count (ANC) ≥1.5 x 109/L
  - Absolute platelet count ≥100 x 109/L
  - Aspartate transaminase (AST) or alanine aminotransferase (ALT) ≤2.5 upper limit of normal (ULN)
  - Total bilirubin ≤1.5 ULN (except for patients with known Gilbert's syndrome)
- Male and female participants must agree to take appropriate measures to prevent pregnancy. Contraceptive measures should be used for 2 weeks prior to trial entry, during the trial and for at least 6 months after last receiving treatment. Acceptable methods of contraception include total abstinence (if this is the patient's usual and preferred lifestyle choice), tubal ligation, combined oral, transdermal or intra-vaginal hormonal contraceptives, medroxyprogesterone injections (e.g. Depo-Provera), copper-banded intra-uterine devices; hormone impregnated intra-uterine systems and vasectomised partners. All methods of contraception (with the exception of total abstinence) should be used in combination with the use of a condom by their male sexual partner for intercourse.

In addition to general criteria

- **Group A** – Accessible tumours for re-biopsy to be taken under local anaesthetic or via ultrasound-guided biopsy
- **Group B** – High-risk histopathological features after surgical resection, i.e. nodal extra-capsular spread and/or tissue resection margin <1 mm as agreed at MDT
- **Group B** – Patients who have previously registered to Group A can be considered for inclusion in Group B

For peer review only - http://bmjopen.bmj.com/site/about/guidelines.xhtml

Box 3: Exclusion criteria – all patients

- Any previous treatment for the same cancer, or previous head and neck malignancy, apart from laser excision of carcinoma in situ, with minimal residual functional deficit, or registration and treatment in Group A prior to surgery
- Patients with cancer of the oropharynx or non-primary cancer will not be included
- Any metastatic disease from any primary site
- Use of an Investigational Medicinal Product (IMP) concurrently or within 4 weeks of starting this trial
- Uncontrolled inter-current illness, which will interfere with patient's trial participation, e.g.:
    - myocardial infarction within 6 months
    - congestive cardiac failure
    - unstable angina
    - symptomatic cardiomyopathy
    - chronic infections
    - active peptic ulcer or liver disease
    - serious psychiatric condition limiting ability to comply with trial protocol
- Clinical evidence of current heart failure (≥New York Heart Association (NYHA) Class II32)
- Clinical evidence of atrial fibrillation (heart rate >100 bpm, within 6 months prior to trial entry)
- Unstable ischaemic heart disease (Myocardial Infarction within 6 months prior to trial entry or angina requiring the use of nitrates greater than once weekly)
- Patients who have a history of Torsades de Pointes (unless all risk factors that contributed to Torsades have been corrected)
- Active gastro-intestinal disease that might limit absorption of study drug, e.g. coeliac disease, Crohn's disease, ulcerative colitis, pancreatic insufficiency
- Evidence of any psychological, familial, sociological or geographical condition potentially hampering protocol compliance
- Participation in another interventional clinical trial whilst taking part in this trial
- Patients who are unable to discontinue any prohibited drug, including live vaccines, and unable to tolerate a washout period for at least 14 days prior to trial entry (including: CYP3A4 inhibitors; CYP3A4 inducers; CYP3A, CYP3A4, CYP2C19, CYP1A2 sensitive substrates or substrates with narrow therapeutic range; P-gp substrates, strong P-gp inhibitors; and BCRP substrates33)
- Patients with any contraindications to cisplatin use
- Clinical judgement by the Investigator that the patient should not participate in the study
- Known hypersensitivity to the study drugs or active substances or excipients of the preparations
- Pregnant or breastfeeding patients
- Significant pre-existing neuropathy which currently interferes with the patient's daily life
- Mean resting corrected QTc interval using the Fridericia formula34 >450 msec (male) and >470 msec (female) (as calculated per institutional standards) obtained from 3 electrocardiograms (ECGs) 2-5 minutes apart at study entry, or congenital long QT syndrome
- Inability to swallow oral medications

For peer review only

For peer review only - http://bmjopen.bmj.com/site/about/guidelines.xhtml

Trial treatments

Group A (pre-operative): patients will receive specified dose of AZD1775 by mouth (PO) twice per day (BID) at 12 hour intervals (2 hours before or after food) for 3 days, commencing on days 1 and 8, with 40 mg/m^2 IV cisplatin delivered over 1 hour on day 8. A missed dose of more than 6 hours should be skipped. If vomiting occurs, patient should not retake the dose but wait until next scheduled dose. Surgery is not to exceed 42 days from start of neo-adjuvant chemotherapy.

Group B (post-operative): patients will receive specified dose of AZD1775 PO BID for 3 days, commencing on days 2, 9, 23 and 30, and 40 mg/m^2 IV cisplatin delivered over 1 hour on days 2, 9, 16, 23 and 30 (days are timed from the start of radiotherapy delivery). Radiotherapy (65Gy and 54Gy in 30 fractions given for 5 days per week over 6 weeks: D1-5, D8-12, D15-19, D22-26, D29-33, D36-40, to high risk and low risk volumes respectively) will commence within 3 months of surgery and given concurrently with chemotherapy.

For both Groups A and B, cisplatin dose banding is not permitted. BSA to be calculated according to local practice; if weight changes >10% then cisplatin dose should be re-calculated.
AZD1775 (morning dose) should be administered prior to cisplatin, and Group B to receive cisplatin one hour before radiotherapy; standard supportive treatment, including premedication with anti-emetics (excluding Aprepitant and Fosaprepitant), is allowed according to standard practice guidelines.

For peer review only

Trial outcome measures

Primary outcome measures: the recommended doses of AZD1775 (determined by the modified TITE-CRM) for Group A and B.

- Group A: the highest safe dose in combination with cisplatin with a predefined target DLT probability of 25% for up to 42 days from start of treatment
- Group B: the MTD in combination with cisplatin/radiotherapy with a target DLT of 30% for up to 12 weeks from the start of treatment.
- Safety profile of AZD1775 in both Group A and B.

Secondary outcome measure: disease-free survival time for both Groups and recorded from commencement of treatment to the date of disease recurrence, patient death or end of trial follow-up period.

Tertiary research outcome measures: pharmacodynamic (PD) effects of AZD1775 and correlation with TP53 mutation status; pharmacokinetic (PK) effects of AZD1775 will be determined; optimise, validate and test feasibility of assays to investigate serum, ctDNA and RNA biomarkers; investigate the feasibility of immune function testing in a multicentre setting; complete pathological response rate for Group A; positive resection margin status in Group A; surgical complication in Group A; and quality of life (QoL) in Group B using European Organization for Research and Treatment of Cancer (EORTC) C30 (version 3.0) [35], EORTC QLQ-H&N35[36] and M.D. Anderson Dysphagia Inventory (MDADI)[37], as summarised in Box 4.
Assessment data will be collected as listed in appendix tables 1 and 2.

Dose limiting toxicity and dose management

Pre-defined DLTs have been specified by the Clinical Investigators (Box 5), and recorded toxicity profiles for the Wisteria treatments are given in appendix table 3. Toxicity is assessed using the National Cancer Institute Common Terminology Criteria for Adverse Events (CTCAE) v4.0[38] unless otherwise specified. Any patient requiring a toxicity-related dose delay of more than 21 days must be discontinued from the study unless there is approval from the Chief Investigator for the patient to continue. For Group A, if any treatment-related Grade ≥3 toxicity develops in week one, cisplatin and AZD1775 are discontinued in week two. For Group B, if calculated GFR falls below 60 mL/min, immediately before any cycle of concomitant chemotherapy, weekly cisplatin should be discontinued and consideration given to substitute with weekly carboplatin (AUC =1.5), according to routine local practice.
In both groups a full blood count is obtained at the beginning of each week. If haematological toxicity occurs, treatment should be modified as per appendix tables 4 and 5. For non-haematological toxicities, treatment should also be modified as per appendix tables 6 to 10.

For peer review only - http://bmjopen.bmj.com/site/about/guidelines.xhtml

Box 4: Trial summaries of Group A and Group B

Summary of Group A

**Setting**	Patients undergoing surgical resection
**Design**	Modified TITE-CRM
**Chemotherapy**	Cisplatin 40 mg/m² IV over 1 hour on Day 8
**AZD1775**	AZD1775 PO BID for 3 days on Days 1-3 and 8-10<br>Dose-recommendation according to the modified TITE-CRM model
**Surgery**	Resection within 42 days of start of neo-adjuvant treatment
**DLT reporting period**	The minimal reporting period is 30 days from start of treatment, but patients will be monitored up to 42 days for delay in surgery due to treatment-related toxicity
**PK samples**	Pharmacokinetic samples will be collected pre- and post- the fifth dose of AZD1775 on Days 3 and 10 (4 samples per patient)
**PD markers**	Assess CDC2, pCDC2, γH2AX, p53, p16, HH3, pHH3, Ki67, C3, CC3, p21, WEE1, pWEE1, PDL-1 and TILS, and other markers of particular interest
**Follow-up**	Clinically for at least 12 weeks from start of treatment

Summary of Group B

**Setting**	Post-operative patients with high risk disease (involved resection margins +/or extracapsular nodal spread) receiving chemo-radiation
**Design**	Modified TITE-CRM
**Chemotherapy**	Cisplatin 40 mg/m² IV over 1 hour on Day 2 of Weeks 1-5 of radiotherapy
**Radiotherapy**	External beam radiation therapy (30 fractions over six weeks) to start within 3 months of surgery. Dose levels are 65 Gy for positive margin, 60 Gy to lymph node levels that have been dissected, and 54 Gy to elective lymph node areas. A dose of 65 Gy may also be applied to areas of gross extra-capsular spread at the discretion of the treating clinician.
**AZD1775**	AZD1775 PO BID for 3 days on Days 2-4 of Weeks 1, 2, 4 and 5 (No treatment with AZD1775 during Weeks 3 and 6).<br>Dose-recommendation will be according to modified TITE-CRM model.
**DLT reporting period**	The minimal reporting period is 56 days (8 weeks) from start of radiotherapy, but patients will be monitored for DLTs up to 84 days (12 weeks), i.e. up to 6 weeks from the end of POCRT
**PK samples**	Pharmacokinetic samples will be collected pre- and post- the fifth dose of AZD1775 on Week 1 – Day 4 (2 samples per patient)
**PD markers**	Assess CDC2, pCDC2, p53, p16, WEE1, pWEE1, PDL-1 and TILS and other markers of particular interest in resection tumour samples
**Follow-up**	Clinically for at least one year from start of treatment

The selection of PD markers to test are different for Group A and Group B as Group A enables examination of samples pre-and post-treatment with AZD1775.

For peer review only

Box 5: Dose limiting toxicities

Patients experiencing any of the following Adverse Reactions (ARs) during the reporting period will be considered to have experienced a DLT:

- Grade 3 or 4 neutropenia lasting for >7 days despite adequate Granulocyte-Colony Stimulating Factor (G-CSF) support
- Grade 4 febrile neutropenia, which includes grade 3 febrile neutropenia accompanied by systemic inflammatory response syndrome / sepsis or other life-threatening consequences
- A third occurrence of Grade 2 or worse neutropenia despite G-CSF support
- Grade 3 or 4 thrombocytopenia lasting ≥7 days
- Any occurrence of Grade 4 anaemia
- Any occurrence of Grade 4 mucositis
- Any occurrence of Grade 3 nausea despite preventative and supportive care according to local practice. Tube feeding is not considered a DLT
- Any occurrence of Grade 4 vomiting despite preventative and supportive care according to local practice. Tube feeding is not considered a DLT
- Any occurrence of Grade 4 diarrhoea despite preventative and supportive care according to local practice
- Any AR which results in an omission of chemotherapy administration and/or study treatment for > 14 days
- A start-of-treatment-to-surgery time of > 42 days in Group A as a result of a treatment-related toxicity
- Grade 3 mucositis lasting > 42 days after the end of treatment in Group B
- An overall treatment time of > 49 days from the first day of radiotherapy in Group B as a result of a treatment-related toxicity
- Any Grade 3 or 4 non-haematological AR (except for fatigue, nausea, vomiting and diarrhoea) for which medical intervention that lasts >7 days is required and the investigators deem that this AR is more severe or prolonged than what would be expected of standard treatment. Tube feeding is not considered a DLT.
- Any clinically significant occurrence which investigators within the Trial Management Group (TMG) agree would place the patient at undue safety risk

If inability to swallow the AZD1775 capsule develops during the course of trial treatment, this event should be noted in the relevant section on the Suspected DLT form and reported immediately. However, this is not considered a DLT in itself.

For peer review only - http://bmjopen.bmj.com/site/about/guidelines.xhtml

Concomitant medications

All concomitant medications received within 14 days before the first dose of study medication and for 12 weeks after the last dose of study medication should be recorded. Medications may be administered for maintenance of existing conditions prior to study enrolment or for a new condition that develops while on study. The treatments and medications listed in appendix 11 are prohibited or to be used with caution while in this study. No other investigational therapy or anticancer agents, other than the study medications, should be given to patients. If such agents are required for a patient, then the patient must first be withdrawn from the study. Live vaccines are not permitted.

Discontinuation of investigational medicinal product

Patients should discontinue trial treatment in the following circumstances: Intolerable toxicity; confirmed disease recurrence; pregnancy; severe non-compliance to protocol; development of any study specific criteria for discontinuation; and investigator decision, for example if the patient requires a prohibited concomitant medication.

Safety monitoring – adverse events and serious adverse events

All medical occurrences which meet the definition of an adverse event (AE) or serious adverse event (SAE) (as defined in Box 6) should be reported. This includes abnormal laboratory findings of grade 3 and above only. Adverse events will be monitored for and reported from date of informed consent until the 12 week follow up visit for Group A and until the 12 month follow up visit for Group B. Investigators should report SAEs within 24 hours of first knowledge of the event, until 12 week follow up visit. After this time, expedited reporting is no longer required and should be reported as an AE. Any post study serious unexpected serious adverse reactions (SUSARs) should be reported within 24 hours. An independent Safety Committee will review all SAEs. Fatal or life threatening SUSARs will be reported to the Medicines and Healthcare products Regulatory Agency (MHRA) and Research Ethics Committee (REC) within 7 days. Detailed follow-up information will be provided within an additional 8 days. All other events categorised as SUSARs will be reported within 15 days. The MHRA and REC will be notified immediately if a significant safety issue is identified during the course of the trial. All SAEs relating to AZD1775 will be reported to AstraZeneca within 24 hours of notification.

Data collection and monitoring

Data will be collected via a set of forms capturing details of eligibility, baseline characteristics, treatment and outcome details. This trial will use an electronic remote data capture (eRDC) system. SAE reporting and Notification of Pregnancy will be paper-based. All missing and ambiguous data will be queried. In all cases it remains the responsibility of the Investigator to ensure that data are accurate. Details regarding data collection for Group A and B are given in appendix tables 1 and 2. The Investigator will permit trial-related monitoring, audits, ethical review, and regulatory inspection(s) at their site, providing direct access to source data/documents.

For peer review only

For peer review only - http://bmjopen.bmj.com/site/about/guidelines.xhtml

Box 6: Adverse event and serious adverse event definitions

Adverse Event

Any untoward medical occurrence in a patient or clinical trial subject administered a medicinal product and which does not necessarily have a causal relationship with this treatment.

Serious Adverse Event

Any untoward medical occurrence or effect that at any dose may:

- Result in death
- Is life-threatening
- Requires hospitalisation or prolongation of existing in-patient hospitalisation
- Results in persistent or significant disability or incapacity
- Is a congenital anomaly/birth defect
- Is considered medically significant by the Clinical Investigator

For peer review only

For peer review only - http://bmjopen.bmj.com/site/about/guidelines.xhtml

Statistical methodology

The AZD1775 dose is allocated to patients according to a modified Bayesian TITE-CRM, using an empiric dose-toxicity model, F(x,β) given as:

$$F(x,\beta) = x^{\exp(\beta)} \; for \; 0 < x < 1$$

where model parameter β is assumed random and follows a normal distribution and will be estimated by its posterior mean[28]. At the point of model-update, patients who have started treatment but have not experienced DLT (and have completed their full DLT assessment) will be included in the probability calculation with a weight equal to the proportion of the full DLT assessment period they have completed. Patients who experience DLT within the assessment period or complete the full assessment period are assigned full weight. The model also enables inclusion of partial patient information that cannot be formally evaluated for DLTs due to withdrawal, treatment discontinuation or death, which are unrelated to treatment, within the DLT assessment period. Table 1 displays the four dose levels of AZD1775 for Group A and Group B. For both groups, dosing starts at level 0 and allows for possible escalation to two higher levels, or de-escalation to a lower dose, as recommended by the TITE-CRM, without skipping untried doses in escalation. The TITE-CRM model aims to recommend the next dose with estimated DLT probability closest to the target DLT level, taking into account the practical considerations as detailed below:

Practical Considerations

- Restriction is applied to avoid skipping of untried doses in escalation
- Stop early due to safety concerns if there is sufficient evidence that the posterior probability of DLT at the lowest dose is greater than the target DLT rate, implying that the lowest dose is too toxic
- Stop early if sufficient patients have been allocated to the current MTD (and remains the recommended dose level for the next cohort if the trial continues) before the full recruitment of 21 patients
- The minimum DLT period is set at 30 days for Group A and 8 weeks for Group B. This means that partial information of no DLT in a patient can only be included in the model if they have been followed up for at least 30 days or 8 weeks in Group A and B respectively. (The full DLT assessment periods are 42 days and 12 weeks in Group A and B respectively.) This feature can easily be accommodated using the TITE-CRM model
- A “look ahead” strategy will be implemented if the next recommended dose level by the modified TITE-CRM model will not be influenced by the outcome of the remaining patient(s) of a particular cohort (DLT or no DLT). By implementing this strategy, the next cohort can be recruited immediately without awaiting the final observations from the current cohort, thus reducing waiting time between cohorts

Analysis of outcome measures

The primary outcome measures are the recommended doses of AZD1775 in Group A and Group B, and the safety profile of the combination therapy. To be DLT evaluable, Group A patients must have received AZD1775 and cisplatin doses scheduled up to and including Day 8; Group B patients must have received at least the first two weeks of treatment. The recommended doses of AZD1775 will be those that have an estimated DLT rate closest to the target DLT rate: 25% for Group A; 30% for Group B. DLT rates and corresponding 90% probability intervals will be reported. Safety will be

For peer review only - http://bmjopen.bmj.com/site/about/guidelines.xhtml

monitored in patients treated with at least one dose of the trial treatment until end of follow-up. All adverse reactions, adverse events, serious adverse events, suspected unexpected serious adverse reactions, deaths, deviations and withdrawals experienced by trial patients, throughout the trial's duration, will be reported separately for Group A and Group B.

Disease-free survival in Group A and Group B patients will be reported separately. Patients will be followed-up for 12 months after which, time to event outcomes will be assessed using the method of Kaplan and Meier. Median diease free survival with corresponding 95% confidence intervals will be reported where appropriate. Further analysis will be performed separately on subsets of Group A and B patients who have completed at least 90% of AZD1775 trial scheduled treatments.

Tertiary outcome measures will be explored and reported descriptively using basic descriptive statistics where appropriate:

- **PD effects of AZD1775 and correlation with TP53 mutation status**: Expression levels of parameters (Box 4) changes over time and correlation with TP53 mutation status will be reported.
- **PK effects of AZD1775**: Blood samples for PK analyses are collected (as scheduled in appendix tables 1 and 2). Circulatory levels of AZD1775 and changes over time will be reported.
- **Complete pathological response rate for Group A**: Pathology data will be reported along with the results of the FFPE tissue samples. Response rate will be reported as the number of evaluable Group A patients showing a response (numerator) divided by the total number of Group A evaluable patients (denominator).
- **Positive resection margin status in Group A**: This will be reported as the number and percentage of Group A patients showing positive resection margins.
- **Surgical complications in Group A**: This will be reported by category of surgical complication with severity and given as numbers and percentages.
- **Quality of Life (QoL) in Group B**: QoL data is gathered over time and will be reported as changes over time and plotted. In addition to the descriptive analyses, the repeated measures over time data may be modelled, if appropriate, with a linear mixed effects model (taking subject correlation into account) using linear or more flexible models.

Interim analyses will be performed once each cohort of patients has been recruited and assessed within the defined assessment timeframe of DLTs. In each meeting, the Safety Committee will be presented with recruitment and safety data, together with a statistical report for next dose-recommendation. Additional meetings might be convened if late onset DLTs were observed. The Safety Committee will decide whether to progress to the recommended dose as indicated by the modified TITE-CRM model. The final analyses will be conducted in two parts: (i) analyses of the primary outcome measures of MTDs for Groups A and B, to be carried out approximately three months after the safety review of the last cohort in each group; (ii) longer term outcomes – including secondary and tertiary outcome measures and updated safety data, to be carried out one year after the end of trial.

For peer review only - http://bmjopen.bmj.com/site/about/guidelines.xhtml

TRIAL ORGANISATION AND STRUCTURE

The Trial is sponsored by the University of Birmingham, UK and conducted under the auspices of the Cancer Research UK Clinical Trials Unit, University of Birmingham, UK according to their local procedures.

The Trial Managemant Group (TMG) will include as a minimum: Chief Investiagtor, Co-Investigators, Lead and Trial Statisticians, Trial Management Team Leader and Trial Co-ordinator (other appropriate key personnel will be invited to attend meetings as required). This group will be responsible for legal obligations, day-to-day running and management of the Trial.

The Safety Committee provides support on decisions surrounding review of DLT information and dose changing decsions. Membership of this group includes the TMG, independent members and selected Principal Investigators.

ETHICS AND DISSEMINATION

The protocol has been approved by the Research Ethics Committee, Edgbaston, West Midlands (REC, reference 16/WM/0501). The first REC approval date was 18/01/2017, with five subsequent approved amendments. This article refers to protocol version 5.0, dated 27/11/2018.

The trial will be performed in accordance with the 18th World Medical Association General Assembly, Helsinki, Finland, June 1964, amended at the 48th World Medical Association General Assembly, Somerset West, Republic of South Africa, October 1996 (website: http://www.wma.net/en/30publications/10policies/b3/index.html); conducted in accordance with the Research Governance Framework for Health and Social Care, the applicable UK Statutory Instruments, (which include the Medicines for Human Use Clinical Trials 2004 and subsequent amendments, The Human Tissue Act 2008) and Good Clinical Practice (GCP) E6(R2); and carried out under a Clinical Trial Authorisation in accordance with the Medicines for Human Use Clinical Trials regulations. The protocol and subsequent amendments will be submitted to and approved by REC prior to circulation. Personal data recorded on all documents will be regarded as strictly confidential and will be handled and stored in accordance with the General Data Protection Regulation 2016/679 and the Data Protection Act (2018).

Trial findings will be published in a peer reviewed journal and disseminated at appropriate conferences, departmental and scientific meetings.

For peer review only

Acknowledgements

Wisteria was supported by Experimental Cancer Medicine Centres (ECMC) funding and by the ECMC Network.

Authors' contributions

The study was conceived by Hisham Mehanna and James Good; designed by Hisham Mehanna, James Good, Anthony Kong, and Christina Yap; and the protocol written by Hisham Mehanna, Anthony Kong, James Good, Christina Yap, Laura Llewellyn, Rhys Mant, Amanda Kirkham, Rachel Spruce, Nikos Batis, Jill Brookes, Joshua Savage, Joe Sacco, Martin Forster and Kevin Harrington.

For peer review only - http://bmjopen.bmj.com/site/about/guidelines.xhtml

Amanda Kirkham, Anthony Kong, James Good and Christina Yap wrote the manuscript with input from all authors.

Funding

Cancer Research UK (code C19677/A20959) and AstraZeneca through the CRUK's Combinations Alliance and Experimental Cancer Medicine Centre (ECMC).

The Combinations Alliance, a Cancer Research UK model, aims to drive academic-industrial partnership, generating novel treatment options that would otherwise unlikely be realised. Novel combination ideas are generated and delivered via the UK's Experimental Cancer Medicine Centre (ECMC) network of clinical and scientific experts working together to accelerate innovation in early-phase oncology research for patient benefit.

Competing interests

HM, AK, RM, JS, CF, JG, RS, Report grants from CRUK, and AZ during the conduct of the study.

JS reports personal fees from Eli Lilly and Company outside the submitted work.

KH reports grants and personal fees from ASTRA ZENECA, during the conduct of the study; personal fees from AMGEN, personal fees from BMS, personal fees from BOEHRINGER INGELHEIM, personal fees from MERCK SERONO, personal fees from MSD, personal fees from PFIZER, outside the submitted work;

HM reports personal fees from BMS, MSD outside the submitted work.

JS reports grants from AstraZeneca, grants from BMS, personal fees from BMS, personal fees from MSD, personal fees from Immunocore, personal fees from Delcath, personal fees from Pierre Fabre, outside the submitted work.

Patient consent

All patients must give written informed consent to the Investigator prior to Trial registration (appendices 16 and 17).

Patient and public involvement

No patient or public involvement contributed to the design of this trial.

Provenance and peer review

Not commissioned; externally peer reviewed.

Ethich approval

Research Ethics Committee (REC, reference 16/WM/0501)

Open access

This is an Open Access article distributed in accordance with the terms in the Creative Commons Attribution (CC BY 4.0) license, which permits others to distribute, remix, adapt and build upon this work, for commercial use, provided the original work is properly cited. See http://creativecommons.org/licenses/by/4.0/

For peer review only

REFERENCES

1. Jemal A, Siegel R, Ward E, *et al*. Cancer Statistics, 2009. *CA Cancer J Clin* 2009;59:225-249.
2. Haddad RI, Shin DM. Recent advances in head and neck cancer. *N Engl J Med* 2008;359:1143-1154.
3. Bernier J, Domenge C, Ozsahin M, *et al*. Postoperative irradiation with or without concomitant chemotherapy for locally advanced head and neck cancer. *N Engl J Med* 2004;350:1945-52.
4. Golden EB, Apetoh L. Radiotherapy and immunogenic cell death. *Sem Rad Onc* 2015;25:11-17.
5. Kang H, Kiess A, Chung CH. Emerging biomarkers in head and neck cancer in the era of genomics. *Nat Rev Clin Oncol* 2015;12:11-26.
6. Olivier M, Hollstein M, Hainaut P. TP53 mutations in human cancers: origins, consequences, and clinical use. *Cold Spring Harb Perspect Biol* 2010;2(1):a001008.
7. Petitjean A, Achatz MI, Borresen-Dale AL, *et al*. TP53 mutations in human cancers: functional selection and impact on cancer prognosis and outcomes. *Oncogene* 2007;26(15):2157-65.
8. Dillon MT, Good JS, Harrington KJ. Selective targeting of the G2/M cell cycle checkpoint to improve the therapeutic index of radiotherapy. *Clin Oncol (R Coll Radiol)* 2014;26:257-85.
9. Hirai H, Iwasawa Y, Okada M, *et al*. Small-molecule inhibition of WEE1 kinase by MK-1775 selectively sensitizes p53-deficient tumour cells to DNA-damaging agents. *Mol Can Ther* 2009;8:2992-3000.
10. Van Linden AA, Baturin D, Ford JB, *et al*. Inhibition of WEE1 sensitizes cancer cells to antimetabolite chemotherapeutics in vitro and in vivo, independent of p53 functionality. *Mol Cancer Ther* 2013;12:2675-84.
11. Magnussen GI, Holm R, Emilsen E, *et al*. High Expression of WEE1 Is Associated with Poor Disease-Free Survival in Malignant Melanoma: Potential for Targeted Therapy. *BMJ Cancer* 2013;13:288.
12. Mir SE, de Witt Hamer PC, Krawxzyk PM, *et al*. In Silico Analysis of Kinase Expression Identifies WEE1 as a Gatekeeper against Mitotic Catastrophe in Glioblastoma. *Cancer Cell* 2010;18:244-57.
13. Wu Z, Doondeea JB, Gholami AM, *et al.* Quantitative chemical proteomics reveals new potential drug targets in head and neck cancer. *Mol Cell Proteomics* 2011;10:M111.011635.
14. Hirai H, Iwasawa Y, Okada M, *et al.* Small-molecule inhibition of WEE1 kinase by MK-1775 selectively sensitizes p53-deficient tumor cells to DNA-damaging agents. *Mol Can Ther* 2009; 8:2992-3000.
15. Bridges KA, Hirai H, Buser CA, *et al.* MK-1775, a novel WEE1 kinase inhibitor, radiosensitizes p53-defective human tumor cells. *Clin Cancer Res* 2011;17: 5638-48.
16. Khanh D, Doroshow JH, Kummar S. WEE1 kinase as a target for cancer therapy. *Cell Cycle* 2013;12:3159-64.
17. Van Linden AA, Baturin D, Ford JB, *et al*. Inhibition of WEE1 sensitizes cancer cells to antimetabolite chemotherapeutics in vitro and in vivo, independent of p53 functionality. *Mol Cancer Ther* 2013;12:2675-84.
18. Osman AA, Monroe MM, Ortega Alves MV, *et al*. Wee-1 Kinase Inhibition Overcomes Cisplatin Resistance Associated with High-Risk TP53 Mutations in Head and Neck Cancer through Mitotic Arrest Followed by Senescence. *Mol Cancer Ther* 2015;14:608-19.

For peer review only - http://bmjopen.bmj.com/site/about/guidelines.xhtml

19. Caretti V, Hiddingh L, Lagerweij T, *et al*. WEE1 kinase inhibition enhances the radiation response of diffuse intrinsic pontine gliomas. *Mol Cancer Ther* 2013;12:141-50.
20. Sarcar B, Kahali S, Prabhu AH, *et al*. Targeting radiation-induced G(2) checkpoint activation with the Wee-1 inhibitor MK-1775 in glioblastoma cell lines. *Mol Cancer Ther* 2011;12:2405-14.
21. Kamak D, Engelke CG, Parsels LA, *et al*. Combined inhibition of WEE1 and PARP1/2 for radiosensitization in pancreatic cancer. *Clin Cancer Res* 2014;20:5085-96.
22. van Linden AA, Baturin D, Ford JB, *et al*. Inhibition of WEE1 sensitizes cancer cells to antimetabolite chemotherapeutics in vitro and in vivo, independent of p53 functionality. *Mol Cancer Ther* 2013;12:2675-84.
23. Guertin AD, Li J, Liu Y, *et al*. Preclinical evaluation of the WEE1 inhibitor MK-1775 as single-agent anticancer therapy. *Mol Cancer Ther* 2013;12:1442-52.
24. Zhong L, Zhang C, Ren G, *et al*. Randomized Phase III Trial of Induction Chemotherapy With Docetaxel, Cisplatin, and Fluorouracil Followed by Surgery Versus Up-Front Surgery in Locally Advanced Resectable Oral Squamous Cell Carcinoma. *J Clin Oncol* 2012;30:1-12.
25. Perrone F, Bossi P, Cortelazzi B, *et al*. TP53 mutations and pathologic complete response to neoadjuvant cisplatin and fluorouracil chemotherapy in resected oral cavity squamous cell carcinoma. *J Clin Oncol* 2010;28:761-6.
26. O'Quigley J, Pepe M, Fisher L. Continual reassessment method. *Biometrics* 1990; 46:33–48.
27. Cheung YK. Dose Finding by the Continual Reassessment Method. *New York: Chapman & Hall/CRC Press* 2011.
28. Cheung YK, Chappell R. Sequential Designs for Phase I Clinical Trials with Late-Onset Toxicities. *Biometrics* 2000;56(4):1177-82.
29. Yap C, Craddock C, Quigley JO, *et al*. Comparing the implementation of a Modified Continual Reassessment Method to a 3+3 Design in a Phase I Acute Myeloid Leukaemia Trial. *Clin Trials* 2013;10(2):75.
30. Do KT, Wilsker D, Balasubramanian P, *et al*. Phase I study of single agent AZD1775 (MK1775), a WEE1 kinase inhibitor, in patients with refractory solid tumors. *J Clin Oncol* 2015;33:3409-3415.
31. Schellens JH, Leijen S, Shapiro G, *et al*. Update on a phase I pharmacologic and pharmacodynamic study of MK1775, a WEE1 tyrosine kinase inhibitor in monotherapy and combination with gemcitabine, cisplatin or carboplatin in patents with advance solid tumour. *ASCO* 2011. Accessed at http://meetinglibrary.asco.org/content/62389
32. Homma A, Inamura N, Oridate N, *et al.* Concomitant Weekly Cisplatin and Radiotherapy for Head and Neck Cancer. *Jpn J Clin Oncol* 2011;41(8)980 – 986.
33. Traynor AM, Richards GM, Hartig GK, *et al*. Comprehensive IMRT Plus Weekly Cisplatin for Advanced Head and Neck Cancer: The University of Wisconsin Experience. *Head Neck* 2010; 32(5):599–606.
34. Jagdis A, Laskin J, Hao D, *et al*. Dose delivery analysis of weekly versus 3-weekly cisplatin concurrent with radiation therapy for locally advanced nasopharyngeal carcinoma (NPC). *Am J Clin Oncol* 2014 Feb;37(1):63-9.
35. Aaronson NK, Ahmedzai S, Bergman B, *et al*. European Organization for Research and Treatment of Cancer QLQ-C30: A Quality-of-Life Instrument for Use in International Clinical Trials in Oncology. *J Natl Cancer Inst* 1993;85:365-376.

For peer review only - http://bmjopen.bmj.com/site/about/guidelines.xhtml

36. Bjordal K, de Graeff A, Fayers PM, *et al*. A 12 Country Field Study of the EORTC QLQ-C30 (version 3.0) and the Head and Neck Cancer Specific Module (EORTC QLQ-H&N35) in Head and Neck Patients. *Eur J Cancer* 2000;36:1796-1807.
37. Chen AY, Frankowski R, Bishop-Leone J, *et al*. The development and validation of a dysphagia-specific quality-of-life questionnaire for patients with head and neck cancer: the M. D. Anderson dysphagia inventory. *Arch Otolaryngol Head Neck Surg* 2001;127:870-6.
38. National Cancer Institute Common Terminology Criteria for Adverse Events (version 4.0). http://ctep.cancer.gov/protocolDevelopment/electronic_applications/ctc.htm

For peer review only

For peer review only - http://bmjopen.bmj.com/site/about/guidelines.xhtml

Appendix Table 1: Group A data collection schedule

	Pre-diagnosis	Baseline within 42 days of trial entry	Baseline within 21 days of trial entry	Baseline within 14 days of trial entry		Week 1			Week 2			End of Treatment visit (within 7 days of D10)	4 Week Follow Up visit +/- 3 days	12 Week Follow Up visit (from start of treatment) +/- 3 days
					Trial Entry	D1[#*]	D2	D3	D8[#]	D9	D10			
Informed consent – optional tumour sample	x													
Optional tumour sample during diagnostic biopsy	x													
Diagnostic CT scan[a]		x												
Informed consent[b]		x												
ECHO/MUGA[c]			x											
Audiogram[d]			x			As clinically indicated								
Medical history[e]				x										
Physical exam[f]				x		x			x					
Weight (kg) and BSA[g]				x		x			x					
Height (cm)				x										
Vital signs[h]				x		x			x					
ECOG status				x		x			x					
ECG[i]				x		x			x			x		
Pregnancy test[j]				x										
Biochemistry[k]				x		x			x			x	x	x
Haematology[l]				x		x			x			x	x	x
Isotopic GFR[m]				x					x					
Adverse events				x		x			x			x	x	x
Concomitant medications[n]				x		x			x			x	x	x
Patient diary						x			x					
Cisplatin administration									x					
AZD1775 administration[o]						AM & PM doses			AM & PM doses					
Surgery[p]						Within 42 days of first date of treatment								
Blood samples[q]						x						x		x
Pharmacokinetic samples[r]								x			x			
Mandatory tumour biopsy[s]								x				At time of Resection		
Collection of FFPE tumour samples														x
Review of surgical complications														x
Disease free survival[t]						On-going								

For peer review only

Group A data collection schedule notes

Day 1 and Day 8 should not occur on a Monday.

***** Day 1 should be within 6 weeks of informed consent.

a Diagnostic Computerised Tomography (CT) with contrast – neck and chest. To be performed within 42 days of trial entry.

b Informed consent must be obtained before any trial procedures occur.

c If significant cardiac history patient required to have ECHO. MUGA can be performed if ECHO is equivocal. To be performed within 21 days of start of trial entry.

d Air and bone conduction audiogram. To be performed within 21 days of trial entry.

e Medical history – comprehensive medical history, demographics, prior treatment.

f Physical examination includes complete review of systems and physical examination of pertinent organ systems and neurological assessment.

g During trial treatment, weight can be performed within 72 hours pre-dose of AZD1775.

h Blood pressure, pulse measurement, temperature and respiratory rate to be performed with the patient sitting for 5 minutes prior to the evaluation.

i Three ECGs, 2-5 minutes apart, to be performed at screening only. At all other time points a single ECG recording should be performed at least 48h pre-dose of AZD1775.

j Women of childbearing potential will require a negative pregnancy test (serum or urine) prior to trial entry.

k Blood samples for sodium, potassium, magnesium, urea, creatinine, calcium, phosphate, albumin, total protein, bilirubin, alkaline phosphatase, AST and/or ALT.

Results of blood samples collected at screening visit can be used for Day 1 eligibility if within 72 hours of first dose of AZD1775. During trial treatment, blood samples can be collected within 72 hours **pre-dose** of AZD1775.

l Haematology – Full Blood Count. Results of haematology samples collected at screening visit can be used for Day 1 eligibility if within 72 hours of first dose of AZD1775. During trial treatment, haematology samples can be collected within 72 hours **pre-dose** of AZD1775.

m If creatinine clearance at screening is ≤ 60 ml/min then an isotopic GFR may be carried out and must be > 60 ml/min. If GFR, calculated immediately before any administration of concomitant chemotherapy, is ≤ 60 ml/min then an isotopic GFR may be carried out and must be > 60 ml/min.

n Concomitant medication to be recorded starting on the date of signing of informed consent, throughout the trial.

o Morning dose to be administered prior to cisplatin on Day 8.

p Surgery must be performed within 42 days of first date of trial treatment but at least 6 days after the last cisplatin dose.

q Blood samples will be collected **pre-dose** on Day 1, at the End of Treatment Visit and at the 12 Week Follow Up Visit. Refer to WISTERIA Laboratory Manual.

r Pharmacokinetic samples to be collected before and 2-5 hours after fifth dose of AZD1775 (before the sixth dose) on Day 3 and Day 10. Refer to WISTERIA Laboratory Manual.

s Tumour biopsy to be collected 2-5 hours after fifth dose of AZD1775 (before the sixth dose) on Day 3 and a biopsy to be taken at time of Resection.. To be taken under local anaesthetic (includes biopsy of lymph nodes taken using ultrasound guided biopsy). Time from biopsy taken to fixing in formalin of the biopsy is limited to 30 minutes. Refer to WISTERIA Laboratory Manual.

t Patients to be followed up for 12 months. Notification required if a patient relapses or dies.

For peer review only

Appendix Table 2: Group B data collection schedule

	Baseline within 21 days of trial entry	Baseline within 14 days of trial entry	Trial Entry	Week 1 – Week 2				Week 3		Week 4 – Week 5				Week 6	Week 7 End of Treatment	Weeks 8-11 Weekly Toxicity Assessments #	Week 12 Follow Up visit	FUP 6 and 12 months from start of treatment
				D1* ~	D2	D3	D4	D1	D2	D1	D2	D3	D4	D1				
Informed consent[a]	x																	
ECHO/MUGA[b]	x																	
Planning CT scan[c]	x																	
Audiogram[d]	x			As clinically indicated														
Medical history[e]		x																
Physical exam[f]		x		x				x		x				x				
Weight (kg) and BSA[g]		x		x				x		x				x				
Height (cm)		x																
Vital signs[h]		x		x				x		x				x				
ECOG status		x		x				x		x				x				
ECG[i]		x		x				x		x				x	x			
Pregnancy test[j]		x																
Biochemistry[k]		x		x				x		x				x	x	x	x	x
Haematology[l]		x		x				x		x				x	x	x	x	x
Isotopic GFR[m]		x		x				x		x								
Adverse events		x		x				x		x				x	x	x	x	x
Concomitant medications[n]		x		x				x		x				x	x	x	x	
Radiotherapy[o]				To start within 3 months of surgery														
AZD1775 administration[p]				N/A	AM & PM Doses			N/A			AM & PM Doses			N/A				
Cisplatin administration[q]					x				x		x			N/A				
Patient diary					x						x							
Quality of Life Questionnaires[r]				x											x		x	x
Swallowing Assessment Questionnaire[r]				x											x		x	x
Blood samples[s]				x											x		x	x
Pharmacokinetic sample[t]							x											
Collection of FFPE tumour samples																	x	
Disease free survival[u]				On-going														

For peer review only

Group B data collection schedule notes:

#	Patients will undergo weekly toxicity assessment during treatment and weekly thereafter until all acute toxicities have resolved to Grade 1 or less; typically for 42-56 days (6-8 weeks), and for no less than 42 days after completion of treatment, giving a total Dose Limiting Toxicity reporting period of 84 days (12 weeks) from start of treatment.
*	Day 1 of week 1 should be within 4 weeks of consent.
~	Week 1 – Day 1 starts on the first day of radiotherapy.
a	Informed consent must be obtained before any trial procedures occur.
b	If significant cardiac history patient required to have ECHO. MUGA can be performed if ECHO is equivocal. To be performed within 21 days of start of trial entry.
c	CT with contrast – neck as part of radiotherapy preparation and planning at Baseline only. To be performed within 21 days of start of trial entry.
d	Air and bone conduction audiogram. To be performed within 21 days of trial entry.
e	Medical history – comprehensive medical history, demographics, prior treatment.
f	Physical examination includes complete review of systems and physical examination of pertinent organ systems and neurological assessment.
g	During trial treatment, weight can be performed within 72 hours pre-dose of AZD1775.
h	Blood pressure, pulse measurement, temperature and respiratory rate to be performed with the patient sitting for 5 minutes prior to the evaluation.
i	Three ECGs, 2-5 minutes apart, to be performed at baseline only. At all other time points a single ECG recording should be performed at least 48 hours **pre-dose** of AZD1775.
j	Women of childbearing potential will require a negative pregnancy test (serum or urine) prior to trial entry.
k	Blood samples for sodium, potassium, magnesium, urea, creatinine, calcium, phosphate, albumin, total protein, bilirubin, alkaline phosphatase, AST and/or ALT. Results of blood samples collected at screening visit can be used for Day 1 eligibility if within 72 hours of first dose of AZD1775. During trial treatment, blood samples can be collected within 72 hours **pre-dose** of AZD1775.
l	Haematology – Full Blood Count. Results of haematology samples collected at screening visit can be used for Day 1 eligibility if within 72 hours of first dose of AZD1775. During trial treatment, haematology samples can be collected within 72 hours **pre-dose** of AZD1775.
m	If creatinine clearance at screening is ≤ 60 ml/min then an isotopic GFR may be carried out and must be > 60 ml/min. If GFR, calculated immediately before any administration of concomitant chemotherapy, is ≤ 60 ml/min then an isotopic GFR may be carried out and must be > 60 ml/min.
n	Concomitant medication to be recorded starting on the date of signing of informed consent, throughout the trial.
o	Radiotherapy to start on Week 1 - Day 1. Refer to the WISTERIA Radiotherapy Guidelines.
p	AZD1775 morning dose to be administered prior to cisplatin and at least 1 hour prior to radiotherapy.
q	Cisplatin to be administered after morning dose of AZD1775 and at least 1 hour prior to radiotherapy.
r	Questionnaires to be completed by patient in clinic at defined visits: **pre-dose** on Week 1 - Day 1, End of Treatment Visit, Week 12 Follow Up Visit, 6 Month Follow Up Visit and 12 Month Follow Up Visit.
s	Blood samples will be collected **pre-dose** on Week 1 - Day 1, at the End of Treatment Visit, the Week 12 Follow Up Visit, the 6 Month Follow Up Visit and the 12 Month Follow Up Visit. Refer to WISTERIA Laboratory Manual.
t	Pharmacokinetic sample to be collected before and 2-5 hours after fifth dose of AZD1775 (before the sixth dose) on Week 1 - Day 4 **only**. Refer to WISTERIA Laboratory Manual.
u	Patients to be followed up for 12 months. Notification required if a patient relapses or dies.

For peer review only - http://bmjopen.bmj.com/site/about/guidelines.xhtml

Appendix Table 3: Toxicity Profiles

Treatment	Very common side effects (1 patient in 10)	Common side effects (1 patient in 100)	Other side effects
AZD1775	• Blood & lymphatic disorders including anaemia & thrombocytopenia<br>• Decreased appetite<br>• Gastrointestinal disorders including diarrhoea, nausea, vomiting & serum electrolyte decreases<br>• Dyspepsia	• Neutropenia	• QTc prolongation (rare, affecting 1 patient in 10,000)
AZD1775 in combination with chemotherapy	• Asthensia<br>• Leukopenia<br>• Loss of strength & weakness	• Fatigue<br>• Febrile neutropenia<br>• Mucosal inflammation<br>• Myalgia<br>• Stomatitis	• Constipation<br>• Gastrointestinal haemorrhage<br>• Lymphopenia<br>• Lymphocyte count decrease<br>• Pancytopenia<br>• Sepsis<br>• Tachycardia
Cisplatin	• Blood & lymphatic disorders including bone marrow failure, thrombocytopenia, leukopenia & anaemia<br>• Metabolism & nutrition disorders including dehydration<br>• Renal & urinary disorders including hyperuricaemia	• Sepsis<br>• Ear disorders including ototoxicity<br>• Cardia disorders including arrhythmia, bradycardia & tachycardia<br>• Inflammation at injection site<br>• Respiratory disorders including dyspnoea & pneumonia	
Radiotherapy	• Tiredness<br>• Pain & difficulty swallowing due to mouth ulceration<br>• Thickened saliva & secretions<br>• Dry mouth<br>• Altered sense of taste<br>• Nausea<br>• Skin soreness & ulceration<br>• Hair loss to are near to radiotherapy site		• Due to swallowing difficulties there is an increased risk of chest infection (pneumonia) during treatment

For peer review only

Appendix Table 4: Haematological Toxicity Dose Management – ANC, Platelets

Symptoms: ANC		Symptoms: Platelets	Action
≥1500/10^9/L	And	≥75000/10^9/L	No cisplatin or AZD1775 dose modification or interruption.
<1500/10^9/L	Or	<75000/10^9/L	Delay cisplatin and AZD1775 by 1 week intervals until recovery. If DLT is reached, discontinue AZD1775 treatment but cisplatin may be resumed if deemed appropriate by the investigators.

Appendix Table 5: Haematological Toxicity Dose Management – Neutropenia, Infection, Febrile Neutropenia

Symptoms	Action
Grade 3 febrile neutropenia (ANC <1000/10^9/L + Temperature ≥38°C) or neutropenic sepsis<br><br>Grade 4 neutropenia (ANC <500/10^9/L >7 days)<br><br>Grade 4 thrombocytopenia (platelet count <25,000/10^9/L >7 days)	Hold cisplatin and AZD1775 dose until recovery. If DLT is reached, discontinue AZD1775 treatment but cisplatin may be resumed if deemed appropriate by the investigators.
Grade 4 febrile neutropenia or Grade 4 infection with neutropenia (both defined as septic shock)<br><br>Thrombocytopenic haemorrhage (gross occult bleeding) associated with a platelet count <50,000/10^9/L	Discontinue cisplatin and AZD1775 treatment.
Febrile neutropenia (≥ 38° C) with or without significant symptoms	Patient to be managed in a hospital setting according to standard procedures, with the urgent initiation of IV antibiotic therapy according to local guidelines.

For peer review only

Appendix Table 6: Non-Haematological Toxicity Dose Management – General Disorders

CTCAE v4.03	Cisplatin	AZD1775
**Grade 0-2**	No dose modification	No dose modification
**Grade 3**	Hold until toxicity resolves to ≤ Grade 1, and then resume at the same dose with no modification if no DLT is reached and if deemed appropriate by the investigators.	Hold until toxicity resolves to ≤ Grade 1, and then resume at the same dose with no modification. Discontinue treatment if DLT is reached
**Grade 4 toxicity** (except anorexia)	Discontinue treatment	Discontinue treatment

Appendix Table 7: Non-Haematological Toxicity Dose Management – Hepatic

CTCAE v4.03	Cisplatin	AZD1775
**Grade 1-2**	No dose modification	No dose modification
**Grade 3** (manifested as elevations in ALT, AST, ALP or bilirubin)	Hold until resolves to Grade ≤1 or baseline, then resume cisplatin with no dose modification if no DLT is reached and if deemed appropriate by the investigators.	Hold until resolves to Grade ≤1 or baseline, then resume study drug with no dose reduction. If not resolved within 28 days discontinue study drug. Discontinue treatment if DLT is reached.
**Grade 4** (Life threatening)	Discontinue treatment	Discontinue treatment

Appendix Table 8: Non-Haematological Toxicity Dose Management – Diarrhoea or Mucositis

CTCAE v4.03	Cisplatin	AZD1775
**Grade 3** (requiring hospitalisation)	Hold until toxicity resolves to ≤ Grade 1, and then resume at the same dose with no modification if no DLT is reached and if deemed appropriate by the investigators.	Hold until toxicity resolves to ≤ Grade 1, and then resume at the same dose with no modification. Discontinue treatment if DLT reached.
**Grade 4**	Discontinue treatment	Discontinue treatment

For peer review only

Appendix Table 9: Non-Haematological Toxicity Dose Management – Renal Toxicity

CTCAE v4.03	Cisplatin	AZD1775
**Grade ≥ 2**	Hold until resolves to Grade ≤1 or baseline. If GFR falls below 60 mL/min, cisplatin should be discontinued and consideration given to substitution with carboplatin AUC =1.5, according to routine local practice.	Hold until toxicity resolves to ≤ Grade 1, and then resume at the same dose with no modification. Discontinue treatment if DLT reached

Appendix Table 10: Non-Haematological Toxicity Dose Management – Neurotoxocity

CTCAE v4.03	Cisplatin	AZD1775
**Grade 1**	No dose modification	No dose modification
**Grade 2**	Hold until toxicity resolves to Grade ≤1. Resume with 1 dose level reduction.	No dose modification
**Grade 3 or 4**	Discontinue treatment	Discontinue treatment

Appendix 11: Disallowed medications and medication to be administered with caution

A list of the main CYP3A4 substrates, inhibitors (strong and moderate) and inducers, CYP2C19 substrates, P-gp substrates and inhibitors and BCRP substrates are shown below:
Please note: Live vaccines are not permitted

This is not an exhaustive list and further details can be found at: www.fda.gov/drugs/developmentapprovalprocess/developmentresources/druginteractionslabeling/ucm093664.htm.

CYP3A4 Inhibitors

Strong

Boceprevir
Clarithromycin
Cobicistat (GS-9350)
Conivaptan
Danoprevir
Elvitegravir
Fosamprenavir
Grapefruit juice
Idelalisib
Indinavir
Itraconazole
Ketoconazole
LCL161
Lopinavir
Mibefradil
Nefazodone
Nelfinavir
Posaconazole
Ritonavir
Saquinavir
Telaprevir
Telithromycin
Tipranavir
Troleandomycin
Voriconazole

For peer review only

Moderate

- ACT-178882
- Amprenavir
- Aprepitant
- Atazanavir
- Casopitant
- Ciprofloxacin
- Crizotinib
- Darunavir
- Dronedarone
- Diltiazem
- Erythromycin
- FK1706
- Fluconazole
- Fosamprenavir
- Imatinib
- Ledipasvir
- Lomitapide
- Netupitant
- Schisandra sphenanthera
- Tofisopam
- Verapamil

Weak

- Almorexant
- Alprazolam
- AMD070
- Amiodarone
- Amlodipine
- Atorvastatin
- Azithromycin
- Berberine
- Bicalutamide
- Blueberry juice
- Chlorzoxazone
- Cilostazol
- Cimetidine
- Clotrimazole
- Cranberry juice
- Cyclosporine
- Daclatasvir
- Delavirdine
- Everolimus
- Faldaprevir
- Fluvoxamine
- Fosaprepitant (IV)
- Ginkgo
- Goldenseal
- GSK1292263
- GSK2248761
- Isoniazid
- Ivacaftor
- Lacidipine
- I Linagliptin
- Lomitapide
- M100240
- Nilotinib
- Oral contraceptives
- Pazopanib
- Peppermint oil
- Propiverine
- Ranitidine
- Ranolazine
- Resveratrol
- Roxithromycin
- Seville orange juice
- Simeprevir
- Sitaxentan
- Suvorexant
- Tabimorelin
- Tacrolimus
- Teriflunomide
- Ticagrelor
- Tipranavir/ritonavir
- Tolvaptan
- Zileuton

For peer review only

CYP3A4 Inducers (Strong and Moderate)

- Avasimibe
- Bosentan
- Carbamazepine
- Efavirenz
- Enzalutamide
- Etravirine
- Genistein
- Lersivirine
- Lopinavir
- Mitotane
- Modafinil
- Nafcillin
- Phenobarbital
- Phenytoin
- Rifabutin
- Rifampin
- Ritonavir
- Semagacestat
- St John's Wort
- Thioridazine
- Tipranavir

CYP3A4 Inducers (Weak)

- Amprenavir
- Aprepitant
- Armodafinil
- AZD 7325
- Bexarotene
- Boceprevir
- Brivaracetam
- Clobazam
- Danshen
- Dexamethasone
- Echinacea
- Eslicarbazepine
- Garlic
- Gingko
- Ginseng
- Glycyrrhizin
- LCL161
- Methylprednisolone

Nevirapine	Raltegravir	Ticagrelor
Oritavancin	Ritonavir	Ticlopidine
Oxcarbazepine	Rufinamide	Topiramate
PA-824	Sorafenib	Troglitazone
Pleconaril	Stribild	Vemurafenib
Prednisone	Telaprevir	Vicriviroc and ritonavir
Quercetin	Terbinafine	Vinblastine

CYP3A and CYP3A4 Sensitive Substrates or Substrates with a Narrow Therapeutic Range

ABT-384	Docetaxol	Nilotinib
Alfentanil	Dofetilide	Nisoldipine
Aprepitant	Doxorubicin	Paclitaxel
Alfuzosin	Ebastine	Pazopanib
Almorexant	Eletriptan	Perospirone
Alpha-Dihydroergocryptine	Elvitegravir	Pimozide
Amiodarone	Eplerenone	Propafenone
Aplaviroc	Ergotamine	Propofol
Aprepitant	Erlotinib	Quetiapine
Astemizole	Etoposide	Quinidine
Atazanavir	Everolimus	Ranolazine
Atorvastatin	Felodipine	Ridaforolimus
Avanafil	Fentanyl	Romidepsin
Bexarotine	Fluticasone	Saquinavir
BIRL 355	Gefitinib	Sildenafil
Bortezomib	Halofantrine	Simeprevir
Bosutinib	Ibrutinib	Simvastatin
Brecanavir	Ifosfamide	Sirolimus
Brotizolam	Imatinib	Tacrolimus
Budesonide	Indinavir	Temsirolimus
Buspirone	Ironotecan	Terfenadine
Capravirine	Ivacaftor	Ticagrelor
Carbamazepine	Ixabepilone	Theophylline
Casopitant	L-771,688	Thioridazine
Cisapride,	Lapatinib	Thiotepa
Conivaptan	Levomethadyl (LAAm)	Tilidine
Cyclophosphamide	Lomitapide	Tipranavir
Cyclosporine	Lopinavir	Tolvaptan
Danoprevir	Lovastatin	Triazolam
Darifenacin	Lurasidone	Tretinoin
Darunavir	Maraviroc,	Ulipristal
Dasatinib	Midazolam	Vardenafil
Dihydroergotamine	Midostaurin	Vicriviroc
Disopyramide	Mosapride	Voclosporin
Dronedarone	Neratinib	

CYP2C19 Sensitive Substrates or Substrates with a Narrow Therapeutic Range

Diazepam	(R)-Lansoprazole	(R)-Mephobarbital
Gliclazide	(S)-Lansoprazole	Omeprazole
Lansoprazole	(S)-Mephenytoin	(R)-Omeprazole

For peer review only

Pantoprazole	Rabeprazole	
(+)-Pantoprazole	Tilidine	

CYP1A2 Sensitive Substrates or Substrates with a Narrow Therapeutic Range

Alosetron	Melatonin	Theophylline
Caffeine	Ramelteon	Tizanidine
Duloxetine	Tacrine	

P-gp Substrates

Colchicine	Indinavir	Vincristine
Digoxin	Paclitaxel	
Fexofenadine	Toptecan	

If a patient requires initiation of digoxin during the study, or is already receiving treatment with digoxin, monitoring of digoxin levels is recommended according to local practice (as the levels of digoxin may increase). Monitoring of digoxin levels is also recommended when the patient has completed dosing with study treatment (as the levels of digoxin may then decrease).

**P-gp Inhibitors (Strong)**	
Cyclosporine	**BCRP Substrates**
Elacridar	Daunorubicin
Erythromycin	Doxorubicin
Itraconazole	Rosuvastatin
Ketocoanzole	Sulfasalazine
LY335979Quinidine	Topotecan
Ritonavir	
Valspodar	
Verapamil	

For peer review only

Appendix 12: Wisteria Radiotherapy Guidelines – Larynx

Volume	Definition and description	
**CTV_6500**	Include the operative bed if a positive margin is present, and the nodal levels with extracapsular spread (ECS), if present. The extent of the operative bed is defined with reference to pre-operative imaging of the primary tumour and to the operative pathology.<br><br><u>Primary tumour</u>: The superior border will lie at the level corresponding to the hyoid on the pre-operative imaging for glottic and subglottic tumours, or 1 cm above the most cranial aspect of the tumour, whichever is most cranial. For supraglottic tumours, the superior border will lie at the level of the tip of the epiglottis on pre-operative imaging, or 1 cm superior to the most cranial aspect of the tumour, whichever is most cranial. The inferior border will lie at the level corresponding with the caudal aspect of the cricoid cartilage on pre-operative imaging, or 1 cm inferior to the most caudal aspect of the tumour, whichever is most caudal. The stoma is included in CTV_6500 if there is subglottic extension of the primary tumour and a positive margin, or ECS in level IV.<br><br><u>Nodes:</u> For areas of nodal ECS, the whole level should be included. If a node with ECS is present at a border between two lymph node levels, the level above/below should also be included as appropriate. For nodal levels included in the CTV_6500, the overlying sternocleidomastoid muscle (SCM) should be included.	
**CTV_6000**	Include:<br>1. The operative bed if there is no positive margin, as defined above.<br>2. Dissected lymph node levels in which there is no ECS if that side of the neck is pN+. The whole level should be included. The SCM should not be included.<br>3. The stoma if there are lymph nodes without ECS in level IV, subglottic extension without a positive margin, or an emergency tracheostomy was performed.	
**CTV_5400**	IA	Not included
	IB	Included if ipsilateral level II is node positive
	II	Always included bilaterally if not already included in CTV_6500 or CTV_6000. If ipsilateral neck is node negative, superior border lies at the inferior border of the transverse process of C1. If ipsilateral neck is node positive, level II is extended superiorly to skull base to include retrostyloid space.
	III	Always included bilaterally
	IVa	Always included bilaterally
	V	Included if ipsilateral neck is node positive
	VI	Included if there is subglottic extension of the primary tumour, soft tissue extension from the primary into the neck, or hypopharyngeal involvement
	RP*	Included if bulky ipsilateral nodes present
	Stoma	If not included in CTV_6500/6000 as defined above

* In the WISTERIA trial, we recommend that the cranial border of the retropharyngeal nodal level is defined as the upper edge of the body of C1 or the upper extent of the hard palate, whichever is more cranial.

For peer review only - http://bmjopen.bmj.com/site/about/guidelines.xhtml

Appendix 13: Wisteria Radiotherapy Guidelines – Hypopharynx

Volume	Definition and description	
**CTV_6500**	Include the operative bed if a positive margin is present, and the nodal levels with ECS, if present. The extent of the operative bed is defined with reference to pre-operative imaging of the primary tumour and to the operative pathology.<br><br><u>Primary tumour</u>: The superior border will lie at corresponding to the level of the hyoid on pre-operative or 1 cm above the most cranial aspect of the tumour on pre-operative imaging, whichever is most cranial. The inferior border will lie at the level corresponding with the caudal aspect of the cricoid cartilage on pre-operative imaging, or 1 cm inferior to the most caudal aspect of the tumour, whichever is most caudal. The stoma (if present) is included in CTV_6500 if there is subglottic extension of the primary tumour and a positive margin, or ECS in level IV.<br><br><u>Nodes:</u> For areas of nodal ECS, the whole level should be included. If a node with ECS is present at a border between two lymph node levels, the level above/below should also be included as appropriate. For nodal levels included in the CTV_6500, the overlying sternocleidomastoid muscle (SCM) should be included.	
**CTV_6000**	Include:<br>1. The operative bed if there is no positive margin, as defined above.<br>2. Dissected lymph node levels in which there is no ECS if that side of the neck is pN+. The whole level should be included. The SCM should not be included.<br>3. The stoma (if present) if there are lymph nodes without ECS in level IV, subglottic extension without a positive margin, or an emergency tracheostomy was performed.	
**CTV_5400**	IA	Not included
	IB	Included if ipsilateral level II is node positive
	II	Always included bilaterally if not already included in CTV_6500 or CTV_6000. If ipsilateral neck is node negative, superior border lies at the inferior border of the transverse process of C1. If ipsilateral neck is node positive, level II is extended superiorly to skull base to include retrostyloid space.
	III	Always included bilaterally
	IV	Always included bilaterally
	V	Included if ipsilateral neck is node positive
	VI	Included if there is subglottic/oesophageal extension of the primary tumour, or soft tissue extension from the primary into the neck<br>Included
	RP*	If not included in CTV_6500/6000 as defined above
	Stoma	Retropharyngeal (RP)

* In the WISTERIA trial, we recommend that the cranial border of the retropharyngeal nodal level is defined as the upper edge of the body of C1 or the upper extent of the hard palate, whichever is more cranial.

For peer review only - http://bmjopen.bmj.com/site/about/guidelines.xhtml

Appendix 14: Wisteria Radiotherapy Guidelines – Lateralised Oral Cavity

Volume	Definition and description	
**CTV_6500**	Include the operative bed if a positive margin is present, and the nodal levels with ECS, if present. The extent of the operative bed is defined with reference to pre-operative imaging of the primary tumour and to the operative pathology.<br><br>Primary tumour: The volume will depend on the primary site. For oral tongue tumours, include the hemi-oral cavity including base of tongue. For buccal mucosal tumours, include the entire mucosa including the retromolar trigone. For retromolar trigone tumours, include the preoperative tumour volume and postoperative tumour bed. Exclude uninvolved bone where possible. Generally, the superior border will be at the level of the hard palate/inferior orbital rim and the inferior border at the level of the hyoid.<br><br>Nodes: For areas of nodal ECS, the whole level should be included. If a node with ECS is present at a border between two lymph node levels, the level above/below should also be included as appropriate. For nodal levels included in the CTV_6500, the overlying sternocleidomastoid muscle (SCM) should be included.	
**CTV_6000**	Include:<br>1. The operative bed if there is no positive margin, as defined above.<br>2. Dissected lymph node levels in which there is no ECS if that side of the neck is pN+. The whole level should be included. The SCM should not be included.	
**CTV_5400**	IA	Included if tumour involves floor of mouth or anterior mandible
	IB	Always included ipsilaterally if not already included in CTV_6500 or CTV_6000
	II	Always included ipsilaterally if not already included in CTV_6500 or CTV_6000. If ipsilateral neck is node negative, superior border lies at the inferior border of the transverse process of C1. If ipsilateral neck is node positive, level II is extended superiorly to skull base. Include contralateral nodes if pN2b/pN3 or primary depth of invasion > 4mm.
		Always included ipsilaterally
	III	Included ipsilaterally if the oropharynx or anterior tongue are involved
	IV	Included if ipsilateral neck is node positive
		Not included
	V	Included if retromolar trigone primary
	VI	Not included
	RP*	Included if retromolar trigone primary

* In the WISTERIA trial, we recommend that the cranial border of the retropharyngeal nodal level is defined as the upper edge of the body of C1 or the upper extent of the hard palate, whichever is more cranial.

Please note: Laterality or otherwise the tumour should be discussed with the surgeon, radiologist and pathologist and confirmed at MDT.

For peer review only

For peer review only - http://bmjopen.bmj.com/site/about/guidelines.xhtml

Appendix 15: Wisteria Radiotherapy Guidelines – Non-Lateralised Oral Cavity

Volume	Definition and description	
**CTV_6500**	Include the operative bed if a positive margin is present, and the nodal levels with ECS, if present. The extent of the operative bed is defined with reference to pre-operative imaging of the primary tumour and to the operative pathology.<br><br><u>Primary tumour</u>: The volume will depend on the primary site. For oral tongue tumours, include the whole oral cavity including base of tongue. Exclude uninvolved bone where possible, but for floor of mouth tumours, consider including the alveolar ridge. Generally, the superior border will be at the level of the hard palate/inferior orbital rim and the inferior border at the level of the hyoid. If a mouth bite is used for floor of mouth tumours, the superior border may be located at the midpoint of the mouthbite.<br><br><u>Nodes:</u> For areas of nodal ECS, the whole level should be included. If a node with ECS is present at a border between two lymph node levels, the level above/below should also be included as appropriate. For nodal levels included in the CTV_6500, the overlying sternocleidomastoid muscle (SCM) should be included.	
**CTV_6000**	Include:<br>1. The operative bed if there is no positive margin, as defined above.<br>2. Dissected lymph node levels in which there is no ECS if that side of the neck is pN+. The whole level should be included. The SCM should not be included.	
**CTV_5400**	IA	Included if tumour involves floor of mouth or anterior mandible
	IB	Always included bilaterally if not already included in CTV_6500 or CTV_6000
	II	Always included bilaterally if not already included in CTV_6500 or CTV_6000. If ipsilateral neck is node negative, superior border lies at the inferior border of the transverse process of C1. If ipsilateral neck is node positive, level II is extended superiorly to skull base.<br>Always included bilaterally.
	III	Included bilaterally if the oropharynx or anterior tongue are involved
	IV	Included if ipsilateral level IV is node positive<br>Not included
	V	Consider including if retromolar trigone primary
	VI	
	RP*	Retropharyngeal (RP)

* In the WISTERIA trial, we recommend that the cranial border of the retropharyngeal nodal level is defined as the upper edge of the body of C1 or the upper extent of the hard palate, whichever is more cranial.

Please note: Laterality or otherwise the tumour should be discussed with the surgeon, radiologist and pathologist and confirmed at MDT.

For peer review only

Appendix 16: Informed Consent Form – Group A

Informed Consent Form

To be used for Group A only

WISTERIA

A Phase I trial of WEE1 inhibition with Chemotherapy and Radiotherapy as adjuvant treatment, and a Window of Opportunity trial with Cisplatin in Patients with Head and Neck Cancer

EudraCT Reference: 2015-003583-37

Site:

Patient Trial Number:

Principal Investigator: ____________________

Screening Number: SCR ______ / ______

(If applicable)

1.1.1

1.1.2

Please initial each box

1. I confirm that I have read and understand the Patient Information Sheet Group A (version .... dated ....) for the above trial. I have had the opportunity to consider the information, ask questions and have had these answered satisfactorily.

2. I understand that my participation is voluntary and that *I am* free to withdraw at any time without giving any reason, without *my* medical care or legal rights being affected. I understand that if I withdraw from treatment my doctor may continue to provide the Trial Office with information that would routinely be collected about me and recorded in my medical notes. I am aware that I can also withdraw consent for this data transfer.

3. I give permission for my *initials, date of birth, and NHS number* to be given to the WISTERIA Trial Office when *I am registered* to the trial *as well as a copy of this consent form*.

4. I understand that relevant sections of my medical notes and data collected during the trial may be looked at by individuals from the WISTERIA Trial Office, regulatory authorities, Sponsor and/or NHS bodies, where it is relevant to my taking part in this research. I understand that this information will be held in a confidential manner. I give permission for these individuals to have access to my records.

For peer review only

5. I understand that anonymised data from the trial may be provided to other third parties (e.g. pharmaceutical companies or other academic institutions) for research, safety monitoring or licensing purposes. I understand that this may involve sending data outside of the United Kingdom to a European country or the United States of America and that my name will not be given to these third parties.

6. I agree to my GP being informed of my participation in this trial.

7. I understand that the WISTERIA Trial Office may access information held by national cancer registries and within national databases to keep in touch with me and to follow up on my health status.

8. I give permission for collection of samples of my blood and tissue to be used in the WISTERIA trial. I understand that samples will be sent to the Institute of Head and Neck Studies and Education (InHANSE), University of Birmingham and other laboratories in the United Kingdom or overseas (including Covance Laboratories Inc., based in the United States of America).

9. I understand that DNA analysis may be performed on the samples taken for the trial.

10. I consent to the storage of samples remaining at the end of the trial and their use in future ethically approved research which may involve genetic analysis, animal or *in vitro* models, commercial or private institutions, and which may take place in the UK or overseas.

11. I understand that information which may identify me will be transferred outside of the hospital and to the <insert name> Clinical Trials Unit at <insert location>. I give permission for this information to be transferred and for the information, including a copy of this consent form to be held by the Clinical Trials Unit so long as strict confidentiality is maintained. *To be deleted as appropriate

12. I agree to take part in the above trial.

**Name of patient**	**Date**	**Signature**
**Name of person taking consent**	**Date**	**Signature**

You must have signed the
Site Signature & Delegation Log

This document was written using CRCTU-ICF-QCD-001, Version 2.0

CANCER RESEARCH UK

For peer review only - http://bmjopen.bmj.com/site/about/guidelines.xhtml

Appendix 17: Informed Consent Form – Group B

Informed Consent Form

To be used for Group B only

WISTERIA

A Phase I trial of WEE1 inhibition with Chemotherapy and Radiotherapy as adjuvant treatment, and a Window of Opportunity trial with Cisplatin in Patients with Head and Neck Cancer

EudraCT Reference: 2015-003583-37

Site: ______________________ **Patient Trial Number:** ☐☐☐

Principal Investigator: ______________________

1.1.3

1.1.4

Please initial each box

13. I confirm that I have read and understand the Patient Information Sheet Group B (version .... dated ....) for the above trial. I have had the opportunity to consider the information, ask questions and have had these answered satisfactorily. ☐

14. I understand that my participation is voluntary and that *I am* free to withdraw at any time without giving any reason, without *my* medical care or legal rights being affected. I understand that if I withdraw from treatment my doctor may continue to provide the Trial Office with information that would routinely be collected about me and recorded in my medical notes. I am aware that I can withdraw consent for this data transfer. ☐

15. I give permission for my *initials, date of birth, and NHS number* to be given to the WISTERIA Trial Office when *I am registered* to the trial *as well as a copy of this consent form*. ☐

16. I understand that relevant sections of my medical notes and data collected during the trial may be looked at by individuals from the WISTERIA Trial Office, regulatory authorities, Sponsor and/or NHS bodies, where it is relevant to my taking part in this research. I understand that this information will be held in a confidential manner. I give permission for these individuals to have access to my records. ☐

17. I understand that anonymised data from the trial may be provided to other third parties (e.g. pharmaceutical companies or other academic institutions) for research, safety monitoring or licensing purposes. I understand that this may involve sending data outside of the United Kingdom to a European country or the United States of America and that my will not be given to these third parties. ☐

For peer review only - http://bmjopen.bmj.com/site/about/guidelines.xhtml

18. I agree to my GP being informed of my participation in this trial.

19. I understand that the WISTERIA Trial Office may access information held by national cancer registries and within national databases to keep in touch with me and to follow up on my health status.

20. I give permission for the collection of samples of my blood and tissue to be used in the WISTERIA trial. I understand that samples will be sent to the Institute of Head and Neck Studies and Education (InHANSE), University of Birmingham and other laboratories in the United Kingdom or overseas (including Covance Laboratories Inc., based in the United States of America).

21. I understand that DNA analysis may be performed on the samples taken for the trial.

22. I consent to the storage of samples remaining at the end of the trial and their use in future ethically approved research which may involve genetic analysis, animal or *in vitro* models, commercial or private institutions, and which may take place in the UK or overseas.

23. I understand that information which may identify me will be transferred outside of the hospital and to the <insert name> Clinical Trials Unit at <insert location>. I give permission for this information to be transferred and for the information, including a copy of this consent form to be held by the Clinical Trials Unit so long as strict confidentiality is maintained. *To be deleted as appropriate

24. I agree to take part in the above trial.

Name of patient	Date	Signature

Name of person taking consent	Date	Signature
You must have signed the Site Signature & Delegation Log		

This document was written using CRCTU-ICF-QCD-001, Version 2.0

CANCER RESEARCH UK

For peer review only

LEGENDS

Figure 1: The differential effect of G_2/M checkpoint inhibitor in sensitizing normal and cancer cells (with TP53 mutations and deficient G_1/S checkpoint) to the effect of DNA damaging agents

Figure 2: Wisteria trial schema. Group A patients were required to have received a minimum treatment of chemotherapy (cicplatin) and AZD1175 in combination to be DLT evaluable. Group B patients were required to have received a minimum of two weeks of treatment (half the total scheduled AZD1775 dose) to be DLT evaluable.

For peer review only

FIGURE 1

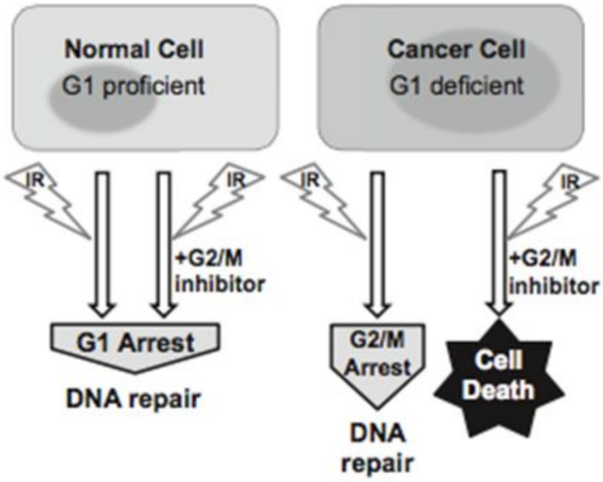

peer review only

For peer review only - http://bmjopen.bmj.com/site/about/guidelines.xhtml

FIGURE 2

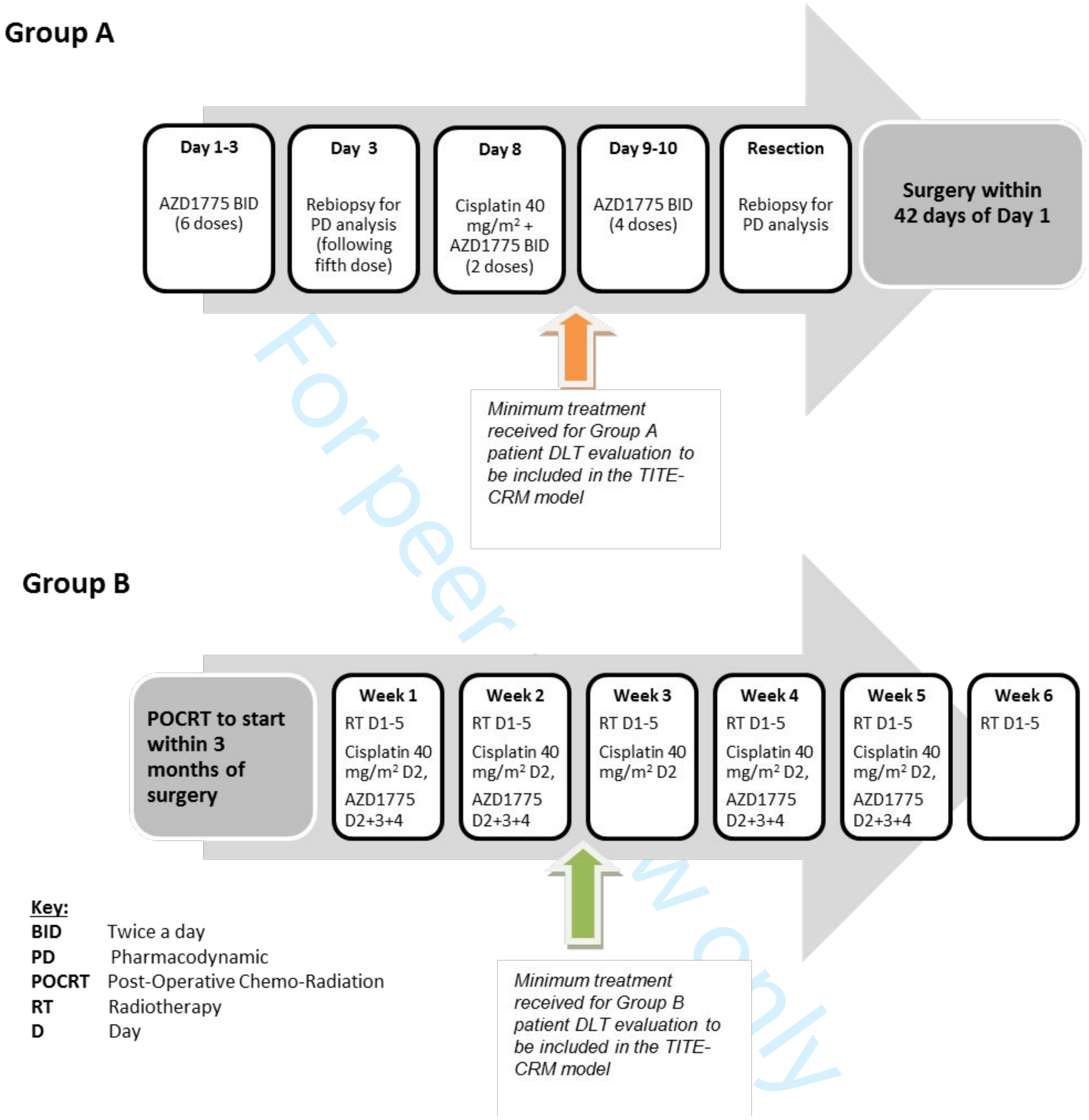

Key:

**BID**	Twice a day
**PD**	Pharmacodynamic
**POCRT**	Post-Operative Chemo-Radiation
**RT**	Radiotherapy
**D**	Day

For peer review only - http://bmjopen.bmj.com/site/about/guidelines.xhtml

Wisteria Trial SPIRIT Checklist

Section/Item	Item Number	Placement of Information
**Administrative information**		
Title	1	Front page
Trial registration	2a	Within Abstract
	2b	Within Abstract and Funding sections
Protocol version	3	Within Ethics and Dissemination section
Funding	4	Funding section
Roles and responsibilities	5a	Front page, Author Affiliations, Authors' Contributions sections
	5b	Front page
	5c	Trial Organisation and Structure section
	5d	Trial Organisation and Structure section
**Introduction**		
Background and rationale	6a	Introduction sections
	6b	N/A
Objectives	7	Methods: Wisteria Trial Objectives section
Trial design	8	Methods: Trial Design section
**Methods**		
Participants, interventions and outcomes:		
Study setting	9	Patient Population, Screening and Consent section
Eligibility criteria	10	Patient Population, Screening and Consent section, boxes 2 and 3
Interventions	11a	Dose Selection section, Table 1, Trial Treatment section
	11b	Dose Limiting Toxicity and Dose Management section, Discontinuation of Investigational Medicinal Product section, Statistical Methods section
	11c	Trial Schema, Trial Treatment section
	11d	Concomitant Medications section, Appendix 11
Outcomes	12	Trial Outcome Measures section, Box 4, Appendices Tables 1 and 2
Participant timelines	13	Trial Schema, Appendices Tables 1 and 2
Sample size	14	Trial Design section, Statistical Methods section
Recruitment	15	Trial Design section
Assignment of interventions (for controlled trials)	16 -17	Not applicable
Data collection, management and analysis:		
Data collection methods	18a	Data Collection and Monitoring section, Appendices 1 and 2
	18b	Analysis of Outcome Measures section
Data management	19	Data Collection and Monitoring section
Statistical methods	20	Statistical Methods section, Analysis of Outcome Measures section
Monitoring:		
Data monitoring	21a	Trial Organisation and Structure section
	21b	Statistical Methods section, Analysis of Outcomes section

For peer review only

Harms	22	Box 5, Safety Monitoring section
Auditing	23	Data Collection and Monitoring section, Trial Organisation and Structure section
**Ethics and dissemination**		
Research ethics approval	24	Ethics and Dissemination abstract and main body section, Ethical Approval section
Protocol amendments	25	Ethics and Dissemination section
Consent or assent	26a	Patient Consent section, Appendices 16 and 17
	26b	Ethics and Dissemination section
Confidentiality	27	Ethics and Dissemination section
Declaration of interests	28	Competing Interests section
Access to data	29	Ethics and Dissemination section, Open Access section
Ancillary and post-trial care	30	Trial Treatment section, Box 4, (re. follow up)
Dissemination policy	31a	Ethics and Dissemination section, Open Access section
**Appendices**		
Informed consent materials	32	Appendices 16 and 17
Biological specimens	33	Tertiary Research Outcome Measures section, Appendices Tables 1 and 2, Analysis of Outcome Measures section

For peer review only

BMJ Open

A Phase I trial of WEE1 inhibition with Chemotherapy and Radiotherapy as adjuvant treatment, and a Window of Opportunity trial with Cisplatin in Patients with Head and Neck Cancer: The WISTERIA Trial Protocol

Journal:	*BMJ Open*
Manuscript ID	bmjopen-2019-033009.R1
Article Type:	Protocol
Date Submitted by the Author:	15-Jan-2020
Complete List of Authors:	Kong, Anthony; University of Birmingham, Institute of Cancer and Genomic Sciences<br>Good, James; University Hospitals Birmingham NHS Foundation Trust<br>Kirkham, Amanda; University of Birmingham, Cancer Research UK Clinical Trials Unit, Institute of Cancer and Genomic Sciences<br>Savage, Joshua; University of Birmingham, Cancer Research UK Clinical Trials Unit, Institute of Cancer and Genomic Sciences<br>Mant, Rhys; University of Birmingham, Cancer Research UK Clinical Trials Unit, Institute of Cancer and Genomic Sciences<br>Llewellyn, Laura; SIMBEC Research Limited<br>Parish, Joanna; University of Birmingham, Institute of Cancer and Genomic Sciences<br>Spruce, Rachel; University of Birmingham, InHANSE, Institute of Cancer and Genomic Sciences<br>Forster, Martin; University College London<br>Schipani, Stefano; University of Glasgow, Beatson West of Scotland Cancer Centre<br>Harrington, Kevin; Institute of Cancer Research<br>Sacco, Joseph; Clatterbridge Cancer Centre NHS Foundation Trust<br>Murray, Patrick; Leeds Teaching Hospitals NHS Trust<br>Middleton, Gary; University of Birmingham, Institute of Immunology and Immunotherapy<br>Yap, Christina; University of Birmingham, Cancer Research UK Clinical Trials Unit, Institute of Cancer and Genomic Sciences<br>Mehanna, H; University of Birmingham, InHANSE, Institute of Cancer and Genomic Sciences
**Primary Subject Heading**:	Oncology
Secondary Subject Heading:	Research methods
Keywords:	head and neck squamous cell carcinoma, TITE-CRM, wee1 inhibitor, chemoradiation, window of opportunity study, wisteria

SCHOLARONE™
Manuscripts

I, the Submitting Author has the right to grant and does grant on behalf of all authors of the Work (as defined in the below author licence), an exclusive licence and/or a non-exclusive licence for contributions from authors who are: i) UK Crown employees; ii) where BMJ has agreed a CC-BY licence shall apply, and/or iii) in accordance with the terms applicable for US Federal Government officers or employees acting as part of their official duties; on a worldwide, perpetual, irrevocable, royalty-free basis to BMJ Publishing Group Ltd ("BMJ") its licensees and where the relevant Journal is co-owned by BMJ to the co-owners of the Journal, to publish the Work in this journal and any other BMJ products and to exploit all rights, as set out in our licence.

The Submitting Author accepts and understands that any supply made under these terms is made by BMJ to the Submitting Author unless you are acting as an employee on behalf of your employer or a postgraduate student of an affiliated institution which is paying any applicable article publishing charge ("APC") for Open Access articles. Where the Submitting Author wishes to make the Work available on an Open Access basis (and intends to pay the relevant APC), the terms of reuse of such Open Access shall be governed by a Creative Commons licence – details of these licences and which Creative Commons licence will apply to this Work are set out in our licence referred to above.

Other than as permitted in any relevant BMJ Author's Self Archiving Policies, I confirm this Work has not been accepted for publication elsewhere, is not being considered for publication elsewhere and does not duplicate material already published. I confirm all authors consent to publication of this Work and authorise the granting of this licence.

For peer review only

A Phase I trial of WEE1 inhibition with Chemotherapy and Radiotherapy as adjuvant treatment, and a Window of Opportunity trial with Cisplatin in Patients with Head and Neck Cancer: The **WISTERIA** Trial Protocol

AUTHORS

Kong A*[1], Good J*[2], Kirkham AJ[3], Savage J[3], Mant R[3], Llewellyn L[8], Parish J[1], Spruce R[1], Forster M[4], Schipani S[5], Harrington K[6], Sacco JJ[7], Murray P[10], Middleton G[9], Yap C‡[3], Mehanna H‡[1]†

*Joint first authors

‡Joint senior authors

†Corresponding author

Corresponding author:

Professor Hisham Mehanna
InHANSE, Institute of Cancer and Genomic Sciences
University of Birmingham
Edgbaston
Birmingham
B15 2TT
H.Mehanna@bham.ac.uk
0121 414 6547

Author Affiliations

[1] Institute of Cancer and Genomic Sciences, University of Birmingham, Edgbaston, Birmingham, UK

[2] University Hospitals Birmingham NHS Foundation Trust, Queen Elizabeth Hospital Birmingham, Mindelsohn Way, Edgbaston, Birmingham, UK

[3] Cancer Research UK Clinical Trials Unit, Institute of Cancer and Genomics Sciences, University of Birmingham, Edgbaston, Birmingham, UK

[4] University College Hospital London, Fitzrovia, London, UK

[5] Beatson West of Scotland Cancer Centre, Institute of Cancer Sciences, University of Glasgow, UK

[6] Royal Marsden/The Institute of Cancer Research, NIHR Biomedical Research Centre, London, UK

[7] The Clatterbridge Cancer Centre, Wirral/University of Liverpool, Liverpool, UK

[8] Simbec Research Ltd, Simbec Research Campus, Pentrebach, Merthyr Tydfil, South Wales, UK

[9] Institute of Immunology and Immunotherapy, University of Birmingham, Edgbaston, Birmingham, UK

[10] St James' University Hospital, Leeds Teaching Hospitals NHS Trust

Keywords (5 max): TITE-CRM, head and neck squamous cell carcinoma, wee1 inhibitor, chemoradiation, window of opportunity study

Word count excluding abstract, figures, tables, boxes and appendices: 6139

For peer review only

ABSTRACT

Introduction

Head and neck squamous cell carcinoma (HNSCC) patients with locally advanced disease often require multimodality treatment with surgery, radiotherapy and/or chemotherapy. Adjuvant radiotherapy with concurrent chemotherapy is offered to patients with high-risk pathological features post-surgery. Whilst cure rates are improved, overall survival remains sub-optimal and treatment has a significant negative impact on quality of life.

Cell cycle checkpoint kinase inhibition is a promising method to selectively potentiate the therapeutic effects of chemo-radiation. Our hypothesis is that combining chemo-radiation with a WEE1 inhibitor will affect the biological response to DNA damage caused by cisplatin and radiation, thereby enhancing clinical outcomes, without increased toxicity. This trial explores the associated effect of WEE1 kinase inhibitor Adavosertib (AZD1775).

Methods and analysis

This Phase I dose-finding, open-label, multicentre trial aims to determine the highest safe dose of AZD1775 in combination with cisplatin chemotherapy pre-operatively (Group A) as a window of opportunity trial, and in combination with post-operative cisplatin-based chemo-radiation (Group B). Modified time-to-event continual reassessment method (TITE-CRM) will determine the recommended dose, recruiting up to 21 patients per group. Primary outcomes are recommended doses with predefined target dose-limiting toxicity probabilities of 25% monitored up to 42 days (Group A), and 30% monitored up to 12 weeks (Group B). Secondary outcomes are disease-free survival times (Groups A and B). Exploratory objectives are evaluation of pharmacodynamic (PD) effects, identification and correlation of potential biomarkers with PD markers of DNA damage, determine rate of resection status and surgical complications for Group A; and quality of life in Group B.

Ethics and dissemination

Research Ethics Committee, Edgbaston, West Midlands (REC reference 16/WM/0501) initial approval received on 18/01/2017. Results will be disseminated via peer-reviewed publication and presentation at international conferences.

Trial Registration

European Medicines Agency (EudraCT: 2015-003583-37); ISRCTN is 76291951 (http://www.isrctn.com/ISRCTN76291951); Clinicaltrials.gov identifier is NCT03028766 (http://clinicaltrials.gov/ct2/show/NCT03028766).

For peer review only

Strengths and Limitations

Strengths of the study:

- The study is one of the first to explore a WEE1 inhibitor in combination with cisplatin and radiotherapy, and the first to do so in head and neck cancer.
- Allows collection of tissues for translational research to examine the pharmacodynamic effects of WEE1 and WEE1 in combination with cisplatin.
- Uses an efficient TITE-CRM design which allows dose assignments to be performed with the flexibility of continual patient accrual or temporary accrual suspension to permit sufficient accumulation of safety information as necessary.

Limitations of the study:

- Additional biopsies in the pre-operative setting may affect the patient enrolment.
- The oral administration of AZD1775 may affect recruitment in post-operative patients if they have difficulty or are unable to swallow.

For peer review only

INTRODUCTION

Head and neck cancers are the sixth most common cancer worldwide[1], with 12,000 new cases per year in the UK (CRUK head and neck cancer statistics). The most common types are squamous cell carcinoma (HNSCC) arising from laryngeal, oral cavity, oropharynx and hypopharyngeal subsites[2].
Primary surgery is the standard of care for cancers of the oral cavity, and for more advanced cancers of the larynx and hypopharynx, whilst (chemo)-radiation is the standard approach to oropharyngeal disease. Post-operative radiotherapy (PORT), or post-operative chemoradiation (POCRT), is often recommended in the setting of high-risk features such as a positive margin, multiple involved nodes, extra-capsular spread, and peri-neural infiltration.
The role of concomitant chemotherapy was established by the Meta-Analysis of Chemotherapy on Head and Neck Cancer (MACH-NC) in 2009[2]. This study pooled individual patient data from 93 trials involving 16,485 patients receiving either definitive local therapy alone (surgery or radiotherapy) or local therapy plus chemotherapy (induction, concomitant or adjuvant). Concomitant therapy was assessed in 50 trials involving 9605 patients; chemotherapy decreased the risk of death with a hazard ratio of 0.81, translating into a 6.5% overall survival advantage at 5 years as a result of improved disease-free survival. No benefit was seen in patients aged over 70 years. This provides robust evidence that the ability of radiotherapy to secure local control can be enhanced by systemic therapy.
Acute toxicity and long-term sequelae of POCRT have a considerable negative health impact, and despite intensive treatment, five year overall survival is sub-optimal at 53%[3]. Loco-regional relapse is particularly difficult to salvage, and local control is therefore closely correlated with overall survival. Morbidity imposed by local recurrence in the absence of distant metastatic disease is considerable and can be extremely challenging to palliate adequately. There is an urgent need to develop novel approaches that achieve improved loco-regional disease control and reduce treatment-related morbidity for this patient group. Achieving this may translate into improved overall survival and an enhancement in patient-related outcome measures.

Chemotherapy, radiotherapy and DNA damage response

Cellular DNA damage response (DDR) is central to the preservation of genomic integrity. Cancer development is a multistep process where deregulation of the DDR contributes to phenotypes such as sustained cell proliferation and resistance to cell death. This increase in endogenous DNA damage necessitates the acquisition of compensatory mechanisms if the cell is to avoid death through uncontrolled genomic instability. Importantly, these compensatory changes may constitute a molecular 'Achilles heel' that is vulnerable to therapeutic exploitation with a new generation of targeted agents. POCRT exploits the differential DDR in malignant and normal tissues to eradicate microscopic residual disease. Ionising radiation (IR) generates a variety of biological changes within irradiated tissue, with double-stranded DNA (dsDNA) breaks being therapeutically significant. IR-induced cell death is also immunogenic, with recruitment of immune effectors to the irradiated tumour micro-environment contributing to a successful outcome[4]. Platinum-based chemotherapy accentuates IR-induced cell death, in part via the suppression of homologous recombination (HR), the primary repair mechanism for dsDNA damage in irradiated cells[4]. Manipulation of the DDR pathway therefore represents a rational means by which the therapeutic index of POCRT might be improved.
Cell cycle checkpoints are integral components of the DDR, allowing the cell to pause progression through the cell cycle to repair DNA damage. Checkpoints are regulated by a network of phosphorylation cascades. *p53*, encoded by *TP53*, is a key regulator of the G_1/S checkpoint. *TP53* mutations, which are seen in 60-70% of HNSCC cases[5], are sufficient to impair the function of this

checkpoint and thereby create a critical reliance on the later G_2/M checkpoint. In addition, p53 function can be inactivated by various mechanisms, including somatic and germline mutations as well as polymorphisms[6,7].
Pharmacological abrogation of the G_2/M checkpoint has been shown to differentially sensitise normal and tumour cells to the effect of DNA damaging agents such as cisplatin and IR[8].

WEE1 kinase and AZD1775

WEE1 kinase is a key regulator of the G_2/M checkpoint and a promising therapeutic target. It is a serine-threonine kinase involved in phosphorylation and inactivation of cyclin-dependent kinase 1 (CDK1), the only one of 14 similar proteins to be indispensable for mitotic entry. Along with a number of other proteins, WEE1 triggers G_2/M arrest in response to DNA damage. However, inhibition of WEE1 leads to high CDK1 activity, allowing cells to progress through the G_2/M checkpoint without the opportunity to repair damaged DNA, leading to potentially catastrophic levels of unrepaired DNA damage induction of cell death[9,10]. WEE1 also has an effect on CDK2 as its inhibition leads to high CDK2 activity and aberrant DNA replication, resulting in stalled replication forks and DNA double-stranded breaks. WEE1 upregulation is seen in a variety of human cancers and is inversely associated with prognosis in some models[11, 12]. Two separate kinomic screens in HNSCC identified WEE1 expression as a particularly strong determinant of cell survival[13, 14], indicating that HNSCC may be a fruitful setting in which to investigate the clinical effects of WEE1 inhibition.
Adavosertib (AZD1775) is a potent, selective small molecule inhibitor of WEE1. It has been shown to potentiate the activity of various chemotherapeutic agents *in vitro* and *in vivo*. Some studies suggest the sensitising effect is only seen in p53-deficient tumours[14-16] although not exclusively[17]. It has also been shown to enhance IR-induced cell death in TP53-mutant cell lines.
Co-exposure of AZD1775 and cisplatin were found to reduce clonogenic survival[18], demonstrating this combination therapy has the ability to overcome cisplatin resistance in HNSCC. Similar effects of this compound on radiation-induced cell death have been seen in models of typically radio-resistant cancer such as pontine glioma[19], glioblastoma[20], and pancreatic adenocarcinoma[21]. Importantly, one study has shown that WEE1 inhibition by AZD1775 sensitises acute myelogenous leukaemia and lung cancer cell lines to cytarabine chemotherapy independently of p53 status[22], suggesting that p53 mutation as a predictive biomarker for response to WEE1 inhibition may be cancer and/or chemotherapy specific. WEE1 has also been implicated in maintaining genomic stability through stabilisation of replication forks - down-regulation reduces replication fork speed during S-phase, generating potentially lethal dsDNA breaks[23]. By impacting both cell cycle progression and DNA damage repair, WEE1 inhibition may potentiate cell death in response to chemotherapy and IR. This suggests that there may be an additive effect on clinical outcome in combination with POCRT, as well as potential synergy.
AZD1775 is being tested in many clinical settings including in combination with docetaxel and cisplatin in head and neck squamous cell carcinoma (NCT02508246) [24], with radiotherapy in childhood pontine glioma (NCT01922076), with temozolomide and radiotherapy in glioblastoma (NCT01849146), and with cisplatin and radiotherapy in cervical cancer (NCT01958658).
In summary, the available mechanistic data lend strong support to combining AZD1775 with cisplatin and with POCRT in the clinic. Given that the predictive effect of TP53 mutation on such combinations has yet to be clinically validated, this study does not prospectively stratify patients based on any such

For peer review only - http://bmjopen.bmj.com/site/about/guidelines.xhtml

clinical or biological characteristic. Biomarker data generated, however, will be important in understanding the mechanism of action and informing future combination studies.

Incorporating AZD1775 into clinical management

A recent randomised phase III study of neoadjuvant chemotherapy in a Chinese population with locally advanced resectable oral cavity cancer showed that docetaxel, cisplatin and 5-fluorouracil (TPF) treatment generated a response rate of 80%, with an exploratory analysis showing that clinical responders had improved overall survival and local control[25]. There was no improvement in overall survival for the study group as a whole compared to the control group without upfront TPF chemotherapy, possibly because patients who would ordinarily have received POCRT received PORT alone in this study. In an earlier study, TP53 mutation conferred increased resistance to pre-operative cisplatin-based chemotherapy in oral cancer[26]. Whilst neoadjuvant chemotherapy has yet to find a definitive role prior to surgery, these findings raise the possibility that chemo-sensitisation may further improve outcomes.

The pre-operative Group A sets out to test this hypothesis by combining one week of AZD1775 alone, with a second week of cisplatin (40 mg/m^2) with AZD1775. This short course of treatment will minimise any delay between diagnosis and definitive surgery and prevent unacceptable delay for the non-responders from getting standard treatment. If this is a promising combination, the long-term aim is to test AZD1775 in the neoadjuvant setting to downstage the disease through treatment with cisplatin and AZD1775 in the pre-operative setting, which may decrease the extent of surgery, and may also reduce the need for PORT/POCRT by reducing the involved margin rate. Many patients with tumours that have high-risk histopathological features develop relapsed disease within the anatomical area treated to a high radiation dose. Chemo- and radio-sensitisation via inhibition of the G_2/M checkpoint by AZD1775 has the potential to improve outcomes with acceptable toxicity. This possibility is the basis of the treatment delivered in the Group B cohort.

METHODS

Wisteria trial objectives

Primary objectives are to determine the recommended dose and safety profile of AZD1775 with cisplatin in the Group A pre-operative (window-of-opportunity) setting, and with cisplatin/radiotherapy in the Group B post-operative setting. Other objectives: obtain information regarding Group A and B disease-free survivals; evaluate AZD1775 pharmacodynamic (PD) effects when administered with cisplatin (Group A); identify and correlate potential predictive biomarkers with PD markers of DNA damage (Group A); determine the rate of R0, R1 or R2 resection status and surgical complication (Group A); and Group B quality of life (QoL).

Trial design

Wisteria is an open-label, dose-finding, multicentre Phase I trial to determine the highest safest dose of AZD1775 in combination with a single dose of cisplatin in the pre-operative setting as a window of opportunity trial (Group A); and to determine the maximum tolerated dose (MTD) of AZD1775 in combination with cisplatin/radiotherapy in the post-operative setting (Group B): both groups recruit and run in parallel (Figure 1). The dose-escalation design is based on the modified time to event

For peer review only - http://bmjopen.bmj.com/site/about/guidelines.xhtml

continual reassessment method (TITE-CRM)[27-30], requiring a maximum of 21 patients per group, and encompassing up to four dose levels of AZD1775.

The MTDs of AZD1775 for both groups are expected to differ given the additional toxicities of radiotherapy in Group B. The DLT monitoring periods are defined in table 1. Conservative target dose limiting toxicity (DLT) rates are selected to minimise the likelihood of compromising individual patient's radical surgery and/or post-operative radiotherapy. To maximise recruitment, reduce trial suspension time between cohorts, whilst balancing safety and optimal patient allocation, screening cohorts of up to five patients will be allowed if the dose has previously been tested. Recruited patients will be allocated to the current recommended dose up to a maximum of five. Patients who become unevaluable may be replaced.

The model will be updated after every two to three evaluable patients, with any subsequent eligible patients (not already receiving treatment) allocated to the latest recommended dose cohort. Subsequent cohorts will be assigned a dose level using all the data observed until either - the MTD is determined, the maximum sample size is reached, or the trial is stopped early due to unacceptable DLT levels at the lowest dose.

Patient population, screening and consent

Group A patients are those with biopsy proven squamous cell carcinoma of the oral cavity, larynx or hypopharynx (having accessible tumours for re-biopsy under local anaesthetic or via ultrasound-guided biopsy), and due to undergo surgery. Patients in Group B consist of those already diagnosed with oral cavity, larynx or hypopharynx squamous cell carcinoma, who have undergone surgery and are considered at risk of relapse after surgery (i.e. with positive margins and/or nodal extracapsular spread). Patients with cancer of the oropharynx will not be included. Since HPV is only routinely tested in oropharyngeal squamous cell carcinoma or unknown primary of squamous cell carcinoma in the NHS, HPV status was not determined or requested for the tumours as part of the study. Patients who meet the criteria will be supplied with the Patient Information Sheet. If informed consent (appendix 1 and 2) is given the patient undergoes a full screening evaluation to ensure they satisfy the eligibility criteria prior to registration (Boxes 1 and 2).

Dose selection

Based on AstraZeneca studies[31, 32], the Wisteria trial uses 3-day dosing, giving AZD1775 weekly to coincide with cisplatin administration thereby potentiating the DNA damage effects. The maximum total dose of AZD1775 1800 mg in combination with 1 dose of 40mg/m^2 cisplatin for Group A is substantially lower than AZD1775 monotherapy dose 2250mg every 21 days (225mg twice per day orally over 2.5 days per week for 2 weeks per 21-day cycle) used in Do *et al* (2015); and although in combination with cisplatin, the highest dose level (Dose Level 2) is likely to be safe.

A similar dose escalation algorithm will be carried out in Group B with a higher acceptable target DLT level of 30% allowing for multiple dosing of cisplatin with radiotherapy in combination with AZD1775[33-35]. Trial doses and schedule are set out in table 1. AZD1775 is administered orally. The feasibility of the oral administration will be evaluated in post-operative patients.

For peer review only

Table 1: Details of the Wisteria TITE-CRM trial design for Groups A and B

	Group A		Group B		
**Maximum Number of Patients**	Up to 21 patients in each Group				
**MTD Definition**	Dose closest to where 25% patients experience a DLT		Dose closest to where 30% patients experience a DLT		
**Minimum Treatment To Be DLT Evaluable**	Patients to have received the minimum of AZD1775 and Cisplatin doses scheduled up to and including day 8		Patients to have received a minimum of the first two weeks of treatment as scheduled		
**DLT Reporting Period**	Minimum of 30 days from start of treatment up to 42 days for delays in surgery due to treatment related toxicity (if this exceeds 42 days then this delay will be classified as a DLT).		Minimum of 56 days (8 weeks) from start of radiotherapy, and up to 84 days (12 weeks), i.e. up to 6 weeks from end of post-operative chemo-radiation therapy.		
**Dose Levels :**	**Cisplatin (iv) over 1 hr** (Day 8)	**AZD1775 PO BID 3 days** (Days 1-3, 8-10)	**Cisplatin (iv) over 1 hr** (Day 2 weeks 1-5)	**Radiotherapy** (5 days per week over 6 weeks)	**AZD1775 PO BID 3 days** (Days 2-4 weeks 1, 2, 4 & 5)
**-1**	40 mg/m^2	75 mg	40 mg/m^2	54-65 Gy in 30#	50 mg
**0 (starting dose level)**		100 mg			75 mg
**1**		125 mg			100 mg
**2**		150 mg			125 mg

(iv) Intravenous; (PO) By Mouth; (BID) Twice A Day; (Gy) Gray - unit of radiation dose; (#) Daily Fractions

For peer review only

Box 1: Inclusion criteria – all patients

- Histologically confirmed diagnosis of oral, laryngeal or hypopharyngeal squamous cell carcinoma
- Multi-Disciplinary Team (MDT) recommendation for surgical resection with curative intent
- Eastern Cooperative Oncology Group (ECOG) performance status 0/131
- Age ≥18 to ≤70 years
- Creatinine clearance, measured by Glomerular Filtration Rate (GFR), ≥ 60 ml/min at baseline calculated using local practice calculation. If this is ≤ 60 ml/min then an isotopic GFR may be carried out and must be > 60 ml/min
- Acceptable cardiac function. If significant cardiac history, then required for patient to have Left Ventricular Ejection Fraction (LVEF) ≥55% by echocardiogram (ECHO) or Multiple Gated Acquisition Scan (MUGA, if ECHO is equivocal)
- Normal liver and bone marrow function:
  - Haemoglobin (Hb) ≥10.0 g/dL or ≥100 g/L
  - Absolute neutrophil count (ANC) ≥1.5 x 109/L
  - Absolute platelet count ≥100 x 109/L
  - Aspartate transaminase (AST) or alanine aminotransferase (ALT) ≤2.5 upper limit of normal (ULN)
  - Total bilirubin ≤1.5 ULN (except for patients with known Gilbert's syndrome)
- Male and female participants must agree to take appropriate measures to prevent pregnancy. Contraceptive measures should be used for 2 weeks prior to trial entry, during the trial and for at least 6 months after last receiving treatment. Acceptable methods of contraception include total abstinence (if this is the patient's usual and preferred lifestyle choice), tubal ligation, combined oral, transdermal or intra-vaginal hormonal contraceptives, medroxyprogesterone injections (e.g. Depo-Provera), copper-banded intra-uterine devices; hormone impregnated intra-uterine systems and vasectomised partners. All methods of contraception (with the exception of total abstinence) should be used in combination with the use of a condom by their male sexual partner for intercourse.

In addition to general criteria

- **Group A** – Accessible tumours for re-biopsy to be taken under local anaesthetic or via ultrasound-guided biopsy
- **Group B** – High-risk histopathological features after surgical resection, i.e. nodal extra-capsular spread and/or tissue resection margin <1 mm as agreed at MDT
- **Group B** – Patients who have previously registered to Group A can be considered for inclusion in Group B

For peer review only

Box 2: Exclusion criteria – all patients

- Any previous treatment for the same cancer, or previous head and neck malignancy, apart from laser excision of carcinoma in situ, with minimal residual functional deficit, or registration and treatment in Group A prior to surgery
- Patients with cancer of the oropharynx or non-primary cancer will not be included
- Any metastatic disease from any primary site
- Use of an Investigational Medicinal Product (IMP) concurrently or within 4 weeks of starting this trial
- Uncontrolled inter-current illness, which will interfere with patient's trial participation, e.g.:
  - myocardial infarction within 6 months
  - congestive cardiac failure
  - unstable angina
  - symptomatic cardiomyopathy
  - chronic infections
  - active peptic ulcer or liver disease
  - serious psychiatric condition limiting ability to comply with trial protocol
- Clinical evidence of current heart failure (≥New York Heart Association (NYHA) Class II32)
- Clinical evidence of atrial fibrillation (heart rate >100 bpm, within 6 months prior to trial entry)
- Unstable ischaemic heart disease (Myocardial Infarction within 6 months prior to trial entry or angina requiring the use of nitrates greater than once weekly)
- Patients who have a history of Torsades de Pointes (unless all risk factors that contributed to Torsades have been corrected)
- Active gastro-intestinal disease that might limit absorption of study drug, e.g. coeliac disease, Crohn's disease, ulcerative colitis, pancreatic insufficiency
- Evidence of any psychological, familial, sociological or geographical condition potentially hampering protocol compliance
- Participation in another interventional clinical trial whilst taking part in this trial
- Patients who are unable to discontinue any prohibited drug, including live vaccines, and unable to tolerate a washout period for at least 14 days prior to trial entry (including: CYP3A4 inhibitors; CYP3A4 inducers; CYP3A, CYP3A4, CYP2C19, CYP1A2 sensitive substrates or substrates with narrow therapeutic range; P-gp substrates, strong P-gp inhibitors; and BCRP substrates33)
- Patients with any contraindications to cisplatin use
- Clinical judgement by the Investigator that the patient should not participate in the study
- Known hypersensitivity to the study drugs or active substances or excipients of the preparations
- Pregnant or breastfeeding patients
- Significant pre-existing neuropathy which currently interferes with the patient's daily life
- Mean resting corrected QTc interval using the Fridericia formula34 >450 msec (male) and >470 msec (female) (as calculated per institutional standards) obtained from 3 electrocardiograms (ECGs) 2-5 minutes apart at study entry, or congenital long QT syndrome
- Inability to swallow oral medications

For peer review only

Trial treatments

Group A (pre-operative): patients will receive specified dose of AZD1775 by mouth (PO) twice per day (BID) at 12 hour intervals (2 hours before or after food) for 3 days, commencing on days 1 and 8, with 40 mg/m^2 IV cisplatin delivered over 1 hour on day 8. A missed dose of more than 6 hours should be skipped. If vomiting occurs, patient should not retake the dose but wait until next scheduled dose. Surgery is not to exceed 42 days from start of neoadjuvant chemotherapy.

Group B (post-operative): patients will receive specified dose of AZD1775 PO BID for 3 days, commencing on days 2, 9, 23 and 30, and 40 mg/m^2 IV cisplatin delivered over 1 hour on days 2, 9, 16, 23 and 30 (days are timed from the start of radiotherapy delivery). Radiotherapy (65Gy and 54Gy in 30 fractions given for 5 days per week over 6 weeks: D1-5, D8-12, D15-19, D22-26, D29-33, D36-40, to high risk and low risk volumes respectively as per Wisteria Radiotherapy Guidelines, see appendices 3-6) will commence within 3 months of surgery and given concurrently with chemotherapy.

For both Groups A and B, cisplatin dose banding is not permitted. BSA to be calculated according to local practice; if weight changes >10% then cisplatin dose should be re-calculated.
AZD1775 (morning dose) should be administered prior to cisplatin, and Group B to receive cisplatin one hour before radiotherapy; standard supportive treatment, including premedication with anti-emetics (excluding Aprepitant and Fosaprepitant), is allowed according to standard practice guidelines.

For peer review only

Trial outcome measures

Primary outcome measures: the recommended doses of AZD1775 (determined by the modified TITE-CRM) for Group A and B.

- Group A: the highest safe dose in combination with cisplatin with a predefined target DLT probability of 25% for up to 42 days from start of treatment
- Group B: the MTD in combination with cisplatin/radiotherapy with a target DLT of 30% for up to 12 weeks from the start of treatment.
- Safety profile of AZD1775 in both Group A and B.

Secondary outcome measure: disease-free survival time for both Groups and recorded from commencement of treatment to the date of disease recurrence, patient death or end of trial follow-up period.

Tertiary research outcome measures: pharmacodynamic (PD) effects of AZD1775 and correlation with TP53 mutation status; pharmacokinetic (PK) effects of AZD1775 will be determined; optimise, validate and test feasibility of assays to investigate serum, ctDNA and RNA biomarkers; investigate the feasibility of immune function testing in a multicentre setting; complete pathological response rate for Group A; positive resection margin status in Group A; surgical complication in Group A; and quality of life (QoL) in Group B using European Organization for Research and Treatment of Cancer (EORTC) C30 (version 3.0) [36], EORTC QLQ-H&N35[37] and M.D. Anderson Dysphagia Inventory (MDADI)[38], as summarised in Box 3.

Assessment data will be collected as listed in appendix tables 7 and 8.

Dose limiting toxicity and dose management

Pre-defined DLTs have been specified by the Clinical Investigators (Box 4), and recorded toxicity profiles for the Wisteria treatments are given in appendix table 9. Toxicity is assessed using the National Cancer Institute Common Terminology Criteria for Adverse Events (CTCAE) v4.0[39] unless otherwise specified. Any patient requiring a toxicity-related dose delay of more than 21 days must be discontinued from the study unless there is approval from the Chief Investigator for the patient to continue. For Group A, if any treatment-related Grade ≥3 toxicity develops in week one, cisplatin and AZD1775 are discontinued in week two. For Group B, if calculated GFR falls below 60 mL/min, immediately before any cycle of concomitant chemotherapy, weekly cisplatin should be discontinued and consideration given to substitute with weekly carboplatin (AUC =1.5), according to routine local practice.

In both groups a full blood count is obtained at the beginning of each week. If haematological toxicity occurs, treatment should be modified as per appendix tables 10 and 11. For non-haematological toxicities, treatment should also be modified as per appendix tables 12 to 16.

For peer review only

Box 3: Trial summaries of Group A and Group B

Summary of Group A

**Setting**	Patients undergoing surgical resection
**Design**	Modified TITE-CRM
**Chemotherapy**	Cisplatin 40 mg/m^2 IV over 1 hour on Day 8
**AZD1775**	AZD1775 PO BID for 3 days on Days 1-3 and 8-10<br>Dose-recommendation according to the modified TITE-CRM model
**Surgery**	Resection within 42 days of start of neoadjuvant treatment
**DLT reporting period**	The minimal reporting period is 30 days from start of treatment, but patients will be monitored up to 42 days for delay in surgery due to treatment-related toxicity
**PK samples**	Pharmacokinetic samples will be collected pre- and post- the fifth dose of AZD1775 on Days 3 and 10 (4 samples per patient)
**PD markers**	Assess CDK1, pCDK1, γH2AX, p53, p16, HH3, pHH3, Ki67, C3, CC3, p21, WEE1, pWEE1, PDL-1 and TILS, and other markers of particular interest
**Follow-up**	Clinically for at least 12 weeks from start of treatment

Summary of Group B

**Setting**	Post-operative patients with high risk disease (involved resection margins +/or extracapsular nodal spread) receiving chemo-radiation
**Design**	Modified TITE-CRM
**Chemotherapy**	Cisplatin 40 mg/m^2 IV over 1 hour on Day 2 of Weeks 1-5 of radiotherapy
**Radiotherapy**	External beam radiation therapy (30 fractions over six weeks) to start within 3 months of surgery. Dose levels are 65 Gy for positive margin, 60 Gy to lymph node levels that have been dissected, and 54 Gy to elective lymph node areas. A dose of 65 Gy may also be applied to areas of gross extra-capsular spread at the discretion of the treating clinician.
**AZD1775**	AZD1775 PO BID for 3 days on Days 2-4 of Weeks 1, 2, 4 and 5 (No treatment with AZD1775 during Weeks 3 and 6).<br>Dose-recommendation will be according to modified TITE-CRM model.
**DLT reporting period**	The minimal reporting period is 56 days (8 weeks) from start of radiotherapy, but patients will be monitored for DLTs up to 84 days (12 weeks), i.e. up to 6 weeks from the end of POCRT
**PK samples**	Pharmacokinetic samples will be collected pre- and post- the fifth dose of AZD1775 on Week 1 – Day 4 (2 samples per patient)
**PD markers**	Assess CDK1, pCDK1, p53, p16, WEE1, pWEE1, PDL-1 and TILS and other markers of particular interest in resection tumour samples
**Follow-up**	Clinically for at least one year from start of treatment

The selection of PD markers to test are different for Group A and Group B as Group A enables examination of samples pre-and post-treatment with AZD1775.

For peer review only

Box 4: Dose-limiting toxicities

Patients experiencing any of the following Adverse Reactions (ARs) during the reporting period will be considered to have experienced a DLT:

- Grade 3 or 4 neutropenia lasting for >7 days despite adequate Granulocyte-Colony Stimulating Factor (G-CSF) support
- Grade 4 febrile neutropenia, which includes grade 3 febrile neutropenia accompanied by systemic inflammatory response syndrome/sepsis or other life-threatening consequences
- A third occurrence of Grade 2 or worse neutropenia despite G-CSF support
- Grade 3 or 4 thrombocytopenia lasting ≥7 days
- Any occurrence of Grade 4 anaemia
- Any occurrence of Grade 4 mucositis
- Any occurrence of Grade 3 nausea despite preventative and supportive care according to local practice. Tube feeding is not considered a DLT
- Any occurrence of Grade 4 vomiting despite preventative and supportive care according to local practice. Tube feeding is not considered a DLT
- Any occurrence of Grade 4 diarrhoea despite preventative and supportive care according to local practice
- Any AR which results in an omission of chemotherapy administration and/or study treatment for > 14 days
- A start-of-treatment-to-surgery time of > 42 days in Group A as a result of a treatment-related toxicity
- Grade 3 mucositis lasting > 42 days after the end of treatment in Group B
- An overall treatment time of > 49 days from the first day of radiotherapy in Group B as a result of a treatment-related toxicity
- Any Grade 3 or 4 non-haematological AR (except for fatigue, nausea, vomiting and diarrhoea) for which medical intervention that lasts >7 days is required and the investigators deem that this AR is more severe or prolonged than what would be expected of standard treatment. Tube feeding is not considered a DLT.
- Any clinically significant occurrence which investigators within the Trial Management Group (TMG) agree would place the patient at undue safety risk

If the inability to swallow the AZD1775 capsule develops during the course of trial treatment, this event should be noted in the relevant section on the Suspected DLT form and reported immediately. However, this is not considered a DLT in itself.

For peer review only

Concomitant medications

All concomitant medications received within 14 days before the first dose of study medication and for 12 weeks after the last dose of study medication should be recorded. Medications may be administered for maintenance of existing conditions prior to study enrolment or for a new condition that develops while on study. The treatments and medications listed in appendix 17 are prohibited or to be used with caution while in this study. No other investigational therapy or anticancer agents, other than the study medications, should be given to patients. If such agents are required for a patient, then the patient must first be withdrawn from the study. Live vaccines are not permitted.

Discontinuation of investigational medicinal product

Patients should discontinue trial treatment in the following circumstances: Intolerable toxicity; confirmed disease recurrence; pregnancy; severe non-compliance to protocol; development of any study-specific criteria for discontinuation; and investigator decision, for example, if the patient requires a prohibited concomitant medication.

Safety monitoring – adverse events and serious adverse events

All medical occurrences which meet the definition of an adverse event (AE) or serious adverse event (SAE) (as defined in Box 5) should be reported. This includes abnormal laboratory findings of grade 3 and above only. Adverse events will be monitored for and reported from date of informed consent until the 12 week follow up visit for Group A and until the 12 month follow up visit for Group B. Investigators should report SAEs within 24 hours of first knowledge of the event, until 12 week follow up visit. After this time, expedited reporting is no longer required and should be reported as an AE. Any post-study serious unexpected serious adverse reactions (SUSARs) should be reported within 24 hours. An independent Safety Committee will review all SAEs. Fatal or life-threatening SUSARs will be reported to the Medicines and Healthcare products Regulatory Agency (MHRA) and Research Ethics Committee (REC) within 7 days. Detailed follow-up information will be provided within an additional 8 days. All other events categorised as SUSARs will be reported within 15 days. The MHRA and REC will be notified immediately if a significant safety issue is identified during the course of the trial. All SAEs relating to AZD1775 will be reported to AstraZeneca within 24 hours of notification.

Data collection and monitoring

Data will be collected via a set of forms capturing details of eligibility, baseline characteristics, treatment and outcome details. This trial will use an electronic remote data capture (eRDC) system. SAE reporting and Notification of Pregnancy will be paper-based. All missing and ambiguous data will be queried. In all cases, it remains the responsibility of the Investigator to ensure that data are accurate. Details regarding data collection for Group A and B are given in appendix tables 7 and 8. The Investigator will permit trial-related monitoring, audits, ethical review, and regulatory inspection(s) at their site, providing direct access to source data/documents.

For peer review only

Box 5: Adverse event and serious adverse event definitions

Adverse Event

Any untoward medical occurrence in a patient or clinical trial subject administered a medicinal product and which does not necessarily have a causal relationship with this treatment.

Serious Adverse Event

Any untoward medical occurrence or effect that at any dose may:

- Result in death
- Is life-threatening
- Requires hospitalisation or prolongation of existing in-patient hospitalisation
- Results in persistent or significant disability or incapacity
- Is a congenital anomaly/birth defect
- Is considered medically significant by the Clinical Investigator

For peer review only

Statistical methodology

The AZD1775 dose is allocated to patients according to a modified Bayesian TITE-CRM, using an empiric dose-toxicity model, F(x,β) given as:

$$F(x,\beta) = x^{\exp(\beta)} \quad for \;\; 0 < x < 1$$

where model parameter β is assumed random and follows a normal distribution and will be estimated by its posterior mean[29]. At the point of model-update, patients who have started treatment but have not experienced DLT (and have completed their full DLT assessment) will be included in the probability calculation with a weight equal to the proportion of the full DLT assessment period they have completed. Patients who experience DLT within the assessment period or complete the full assessment period are assigned full weight. The model also enables inclusion of partial patient information that cannot be formally evaluated for DLTs due to withdrawal, treatment discontinuation or death, which are unrelated to treatment, within the DLT assessment period.

Table 1 displays the four dose levels of AZD1775 for Group A and Group B. For both groups, dosing starts at level 0 and allows for possible escalation to two higher levels, or de-escalation to a lower dose, as recommended by the TITE-CRM, without skipping untried doses in escalation. The TITE-CRM model aims to recommend the next dose with estimated DLT probability closest to the target DLT level, taking into account the practical considerations as detailed below:

Practical Considerations

- Restriction is applied to avoid skipping of untried doses in escalation
- Stop early due to safety concerns if there is sufficient evidence that the posterior probability of DLT at the lowest dose is greater than the target DLT rate, implying that the lowest dose is too toxic
- Stop early if sufficient patients have been allocated to the current MTD (and remains the recommended dose level for the next cohort if the trial continues) before the full recruitment of 21 patients
- The minimum DLT period is set at 30 days for Group A and 8 weeks for Group B. This means that partial information of no DLT in a patient can only be included in the model if they have been followed up for at least 30 days or 8 weeks in Group A and B respectively. (The full DLT assessment periods are 42 days and 12 weeks in Group A and B respectively.) This feature can easily be accommodated using the TITE-CRM model
- A “look ahead” strategy will be implemented if the next recommended dose level by the modified TITE-CRM model will not be influenced by the outcome of the remaining patient(s) of a particular cohort (DLT or no DLT). By implementing this strategy, the next cohort can be recruited immediately without awaiting the final observations from the current cohort, thus reducing waiting time between cohorts

Analysis of outcome measures

The primary outcome measures are the recommended doses of AZD1775 in Group A and Group B, and the safety profile of the combination therapy. To be DLT evaluable, Group A patients must have received AZD1775 and cisplatin doses scheduled up to and including Day 8; Group B patients must have received at least the first two weeks of treatment. The recommended doses of AZD1775 will be those that have an estimated DLT rate closest to the target DLT rate: 25% for Group A; 30% for Group B. DLT rates and corresponding 90% probability intervals will be reported. Safety will be monitored in

For peer review only

patients treated with at least one dose of the trial treatment until end of follow-up. All adverse reactions, adverse events, serious adverse events, suspected unexpected serious adverse reactions, deaths, deviations and withdrawals experienced by trial patients, throughout the trial's duration, will be reported separately for Group A and Group B.

Disease-free survival in Group A and Group B patients will be reported separately. Patients will be followed-up for 12 months after which, time to event outcomes will be assessed using the method of Kaplan and Meier. Median disease-free survival with corresponding 95% confidence intervals will be reported where appropriate. Further analysis will be performed separately on subsets of Group A and B patients who have completed at least 90% of AZD1775 trial scheduled treatments.

Tertiary outcome measures will be explored and reported descriptively using basic descriptive statistics where appropriate:

- **PD effects of AZD1775 and correlation with TP53 mutation status**: Expression levels of parameters (Box 3) changes over time and correlation with TP53 mutation status will be reported.
- **PK effects of AZD1775**: Blood samples for PK analyses are collected (as scheduled in appendix tables 7 and 8). Circulatory levels of AZD1775 and changes over time will be reported.
- **Complete pathological response rate for Group A**: Pathology data will be reported along with the results of the FFPE tissue samples. Response rate will be reported as the number of evaluable Group A patients showing a response (numerator) divided by the total number of Group A evaluable patients (denominator).
- **Positive resection margin status in Group A**: This will be reported as the number and percentage of Group A patients showing positive resection margins.
- **Surgical complications in Group A**: This will be reported by category of surgical complication with severity and given as numbers and percentages.
- **Quality of Life (QoL) in Group B**: QoL data is gathered over time and will be reported as changes over time and plotted. In addition to the descriptive analyses, the repeated measures over time data may be modelled, if appropriate, with a linear mixed effects model (taking subject correlation into account) using linear or more flexible models.

Interim analyses will be performed once each cohort of patients has been recruited and assessed within the defined assessment timeframe of DLTs. In each meeting, the Safety Committee will be presented with recruitment and safety data, together with a statistical report for next dose-recommendation. Additional meetings might be convened if late onset DLTs were observed. The Safety Committee will decide whether to progress to the recommended dose as indicated by the modified TITE-CRM model. The final analyses will be conducted in two parts: (i) analyses of the primary outcome measures of MTDs for Groups A and B, to be carried out approximately three months after the safety review of the last cohort in each group; (ii) longer term outcomes – including secondary and tertiary outcome measures and updated safety data, to be carried out one year after the end of trial.

TRIAL ORGANISATION AND STRUCTURE

The Trial is sponsored by the University of Birmingham, UK and conducted under the auspices of the Cancer Research UK Clinical Trials Unit, University of Birmingham, UK according to their local procedures.

For peer review only

The Trial Management Group (TMG) will include as a minimum: Chief Investigator, Co-Investigators, Lead and Trial Statisticians, Trial Management Team Leader and Trial Co-ordinator (other appropriate key personnel will be invited to attend meetings as required). This group will be responsible for legal obligations, day-to-day running and management of the Trial.

The Safety Committee provides support on decisions surrounding the review of DLT information and dose changing decisions. Membership of this group includes the TMG, independent members and selected Principal Investigators.

ETHICS AND DISSEMINATION

The protocol has been approved by the Research Ethics Committee, Edgbaston, West Midlands (REC, reference 16/WM/0501). The first REC approval date was 18/01/2017, with five subsequent approved amendments. This article refers to protocol version 5.0, dated 27/11/2018.

The trial will be performed in accordance with the 18th World Medical Association General Assembly, Helsinki, Finland, June 1964, amended at the 48th World Medical Association General Assembly, Somerset West, Republic of South Africa, October 1996 (website: http://www.wma.net/en/30publications/10policies/b3/index.html); conducted in accordance with the Research Governance Framework for Health and Social Care, the applicable UK Statutory Instruments, (which include the Medicines for Human Use Clinical Trials 2004 and subsequent amendments, The Human Tissue Act 2008) and Good Clinical Practice (GCP) E6(R2); and carried out under a Clinical Trial Authorisation in accordance with the Medicines for Human Use Clinical Trials regulations. The protocol and subsequent amendments will be submitted to and approved by REC prior to circulation. Personal data recorded on all documents will be regarded as strictly confidential and will be handled and stored in accordance with the General Data Protection Regulation 2016/679 and the Data Protection Act (2018).

Trial findings will be published in a peer-reviewed journal and disseminated at appropriate conferences, departmental and scientific meetings.

Acknowledgements

Wisteria was supported by Experimental Cancer Medicine Centres (ECMC) funding and by the ECMC Network.

Authors' contributions

The study was conceived by Hisham Mehanna and James Good; designed by Hisham Mehanna, James Good, Anthony Kong, and Christina Yap; and the protocol written by Hisham Mehanna, Anthony Kong, James Good, Christina Yap, Laura Llewellyn, Rhys Mant, Amanda Kirkham, Rachel Spruce, Joshua Savage (JSS), Joseph Sacco (JS), Martin Forster, Joanna Parish, Martin Forster, Stefano Schipani, Patrick Murray, Gary Middleton and Kevin Harrington. Amanda Kirkham, Anthony Kong, James Good, Joshua Savage and Christina Yap wrote the manuscript with input from all authors.

Funding

Cancer Research UK (code C19677/A20959) and AstraZeneca through the CRUK's Combinations Alliance and Experimental Cancer Medicine Centre (ECMC). AstraZeneca provides AZD1775 to

For peer review only

participating sites free-of-charge, and was consulted over the trial design but are not involved in the trial management group or safety committee.

The Combinations Alliance, a Cancer Research UK model, aims to drive academic-industrial partnership, generating novel treatment options that would otherwise unlikely be realised. Novel combination ideas are generated and delivered via the UK's Experimental Cancer Medicine Centre (ECMC) network of clinical and scientific experts working together to accelerate innovation in early-phase oncology research for patient benefit.

Competing interests

HM, AK, RM, JS, CF, JG, RS, Report grants from CRUK, and AZ during the conduct of the study.

JSS reports personal fees from Eli Lilly and Company outside the submitted work.

KH reports grants and personal fees from ASTRA ZENECA, during the conduct of the study; personal fees from AMGEN, personal fees from BMS, personal fees from BOEHRINGER INGELHEIM, personal fees from MERCK SERONO, personal fees from MSD, personal fees from PFIZER, outside the submitted work.

HM reports personal fees from BMS, MSD outside the submitted work.

JS reports grants from AstraZeneca, grants from BMS, personal fees from BMS, personal fees from MSD, personal fees from Immunocore, personal fees from Delcath, personal fees from Pierre Fabre, outside the submitted work.

Patient consent

All patients must give written informed consent to the Investigator prior to Trial registration.

Patient and public involvement

No patient or public involvement contributed to the design of this trial.

Provenance and peer review

Not commissioned; externally peer-reviewed.

Ethics approval

Research Ethics Committee (REC reference 16/WM/0501).

Open access

This is an Open Access article distributed in accordance with the terms in the Creative Commons Attribution (CC BY 4.0) license, which permits others to distribute, remix, adapt and build upon this work, for commercial use, provided the original work is properly cited. See http://creativecommons.org/licenses/by/4.0/

For peer review only

REFERENCES

1. Jemal A, Siegel R, Ward E, *et al*. Cancer Statistics, 2009. *CA Cancer J Clin* 2009;59:225-249.
2. Haddad RI, Shin DM. Recent advances in head and neck cancer. *N Engl J Med* 2008;359:1143-1154.
3. Bernier J, Domenge C, Ozsahin M, *et al*. Postoperative irradiation with or without concomitant chemotherapy for locally advanced head and neck cancer. *N Engl J Med* 2004;350:1945-52.
4. Golden EB, Apetoh L. Radiotherapy and immunogenic cell death. *Sem Rad Onc* 2015;25:11-17.
5. Kang H, Kiess A, Chung CH. Emerging biomarkers in head and neck cancer in the era of genomics. *Nat Rev Clin Oncol* 2015;12:11-26.
6. Olivier M, Hollstein M, Hainaut P. TP53 mutations in human cancers: origins, consequences, and clinical use. *Cold Spring Harb Perspect Biol* 2010;2(1):a001008.
7. Petitjean A, Achatz MI, Borresen-Dale AL, *et al*. TP53 mutations in human cancers: functional selection and impact on cancer prognosis and outcomes. *Oncogene* 2007;26(15):2157-65.
8. Dillon MT, Good JS, Harrington KJ. Selective targeting of the G2/M cell cycle checkpoint to improve the therapeutic index of radiotherapy. *Clin Oncol (R Coll Radiol)* 2014;26:257-85.
9. Hirai H, Iwasawa Y, Okada M, *et al*. Small-molecule inhibition of WEE1 kinase by MK-1775 selectively sensitizes p53-deficient tumour cells to DNA-damaging agents. *Mol Can Ther* 2009;8:2992-3000.
10. Van Linden AA, Baturin D, Ford JB, *et al*. Inhibition of WEE1 sensitizes cancer cells to antimetabolite chemotherapeutics in vitro and in vivo, independent of p53 functionality. *Mol Cancer Ther* 2013;12:2675-84.
11. Magnussen GI, Holm R, Emilsen E, *et al*. High Expression of WEE1 Is Associated with Poor Disease-Free Survival in Malignant Melanoma: Potential for Targeted Therapy. *BMJ Cancer* 2013;13:288.
12. Mir SE, de Witt Hamer PC, Krawxzyk PM, *et al*. In Silico Analysis of Kinase Expression Identifies WEE1 as a Gatekeeper against Mitotic Catastrophe in Glioblastoma. *Cancer Cell* 2010;18:244-57.
13. Wu Z, Doondeea JB, Gholami AM, *et al.* Quantitative chemical proteomics reveals new potential drug targets in head and neck cancer. *Mol Cell Proteomics* 2011;10:M111.011635.
14. Hirai H, Iwasawa Y, Okada M, *et al.* Small-molecule inhibition of WEE1 kinase by MK-1775 selectively sensitizes p53-deficient tumor cells to DNA-damaging agents. *Mol Can Ther* 2009; 8:2992-3000.
15. Bridges KA, Hirai H, Buser CA, *et al.* MK-1775, a novel WEE1 kinase inhibitor, radiosensitizes p53-defective human tumor cells. *Clin Cancer Res* 2011;17: 5638-48.
16. Khanh D, Doroshow JH, Kummar S. WEE1 kinase as a target for cancer therapy. *Cell Cycle* 2013;12:3159-64.
17. Van Linden AA, Baturin D, Ford JB, *et al*. Inhibition of WEE1 sensitizes cancer cells to antimetabolite chemotherapeutics in vitro and in vivo, independent of p53 functionality. *Mol Cancer Ther* 2013;12:2675-84.
18. Osman AA, Monroe MM, Ortega Alves MV, *et al*. Wee-1 Kinase Inhibition Overcomes Cisplatin Resistance Associated with High-Risk TP53 Mutations in Head and Neck Cancer through Mitotic Arrest Followed by Senescence. *Mol Cancer Ther* 2015;14:608-19.
19. Caretti V, Hiddingh L, Lagerweij T, *et al*. WEE1 kinase inhibition enhances the radiation response of diffuse intrinsic pontine gliomas. *Mol Cancer Ther* 2013;12:141-50.

For peer review only

20. Sarcar B, Kahali S, Prabhu AH, *et al*. Targeting radiation-induced G(2) checkpoint activation with the Wee-1 inhibitor MK-1775 in glioblastoma cell lines. *Mol Cancer Ther* 2011;12:2405-14.
21. Kamak D, Engelke CG, Parsels LA, *et al*. Combined inhibition of WEE1 and PARP1/2 for radiosensitization in pancreatic cancer. *Clin Cancer Res* 2014;20:5085-96.
22. van Linden AA, Baturin D, Ford JB, *et al*. Inhibition of WEE1 sensitizes cancer cells to antimetabolite chemotherapeutics in vitro and in vivo, independent of p53 functionality. *Mol Cancer Ther* 2013;12:2675-84.
23. Guertin AD, Li J, Liu Y, *et al*. Preclinical evaluation of the WEE1 inhibitor MK-1775 as single-agent anticancer therapy. *Mol Cancer Ther* 2013;12:1442-52.
24. Méndez E, Rodriguez CP, Kao MC, *et al*. A Phase I Clinical Trial of AZD1775 in Combination with Neoadjuvant Weekly Docetaxel and Cisplatin before Definitive Therapy in Head and Neck Squamous Cell Carcinoma. *Clin Cancer Res* 2018;24: 2740-2748
25. Zhong L, Zhang C, Ren G, *et al*. Randomized Phase III Trial of Induction Chemotherapy With Docetaxel, Cisplatin, and Fluorouracil Followed by Surgery Versus Up-Front Surgery in Locally Advanced Resectable Oral Squamous Cell Carcinoma. *J Clin Oncol* 2012;30:1-12.
26. Perrone F, Bossi P, Cortelazzi B, *et al*. TP53 mutations and pathologic complete response to neoadjuvant cisplatin and fluorouracil chemotherapy in resected oral cavity squamous cell carcinoma. *J Clin Oncol* 2010;28:761-6.
27. O'Quigley J, Pepe M, Fisher L. Continual reassessment method. *Biometrics* 1990; 46:33–48.
28. Cheung YK. Dose Finding by the Continual Reassessment Method. *New York: Chapman & Hall/CRC Press* 2011.
29. Cheung YK, Chappell R. Sequential Designs for Phase I Clinical Trials with Late-Onset Toxicities. *Biometrics* 2000;56(4):1177-82.
30. Yap C, Craddock C, Quigley JO, *et al*. Comparing the implementation of a Modified Continual Reassessment Method to a 3+3 Design in a Phase I Acute Myeloid Leukaemia Trial. *Clin Trials* 2013;10(2):75.
31. Do KT, Wilsker D, Balasubramanian P, *et al*. Phase I study of single agent AZD1775 (MK1775), a WEE1 kinase inhibitor, in patients with refractory solid tumors. *J Clin Oncol* 2015;33:3409-3415.
32. Schellens JH, Leijen S, Shapiro G, *et al*. Update on a phase I pharmacologic and pharmacodynamic study of MK1775, a WEE1 tyrosine kinase inhibitor in monotherapy and combination with gemcitabine, cisplatin or carboplatin in patents with advance solid tumour. *ASCO* 2011. Accessed at http://meetinglibrary.asco.org/content/62389
33. Homma A, Inamura N, Oridate N, *et al.* Concomitant Weekly Cisplatin and Radiotherapy for Head and Neck Cancer. *Jpn J Clin Oncol* 2011;41(8)980 – 986.
34. Traynor AM, Richards GM, Hartig GK, *et al*. Comprehensive IMRT Plus Weekly Cisplatin for Advanced Head and Neck Cancer: The University of Wisconsin Experience. *Head Neck* 2010; 32(5):599–606.
35. Jagdis A, Laskin J, Hao D, *et al*. Dose delivery analysis of weekly versus 3-weekly cisplatin concurrent with radiation therapy for locally advanced nasopharyngeal carcinoma (NPC). *Am J Clin Oncol* 2014 Feb;37(1):63-9.
36. Aaronson NK, Ahmedzai S, Bergman B, *et al.* European Organization for Research and Treatment of Cancer QLQ-C30: A Quality-of-Life Instrument for Use in International Clinical Trials in Oncology. *J Natl Cancer Inst* 1993;85:365-376.

37. Bjordal K, de Graeff A, Fayers PM, *et al*. A 12 Country Field Study of the EORTC QLQ-C30 (version 3.0) and the Head and Neck Cancer Specific Module (EORTC QLQ-H&N35) in Head and Neck Patients. *Eur J Cancer* 2000;36:1796-1807.
38. Chen AY, Frankowski R, Bishop-Leone J, *et al*. The development and validation of a dysphagia-specific quality-of-life questionnaire for patients with head and neck cancer: the M. D. Anderson dysphagia inventory. *Arch Otolaryngol Head Neck Surg* 2001;127:870-6.
39. National Cancer Institute Common Terminology Criteria for Adverse Events (version 4.0). http://ctep.cancer.gov/protocolDevelopment/electronic_applications/ctc.htm

FIGURE LEGENDS

Figure 1: Wisteria trial schema. Group A patients were required to have received a minimum treatment of chemotherapy (cisplatin) and AZD1175 in combination to be DLT evaluable. Group B patients were required to have received a minimum of two weeks of treatment (half the total scheduled AZD1775 dose) to be DLT evaluable.

For peer review only

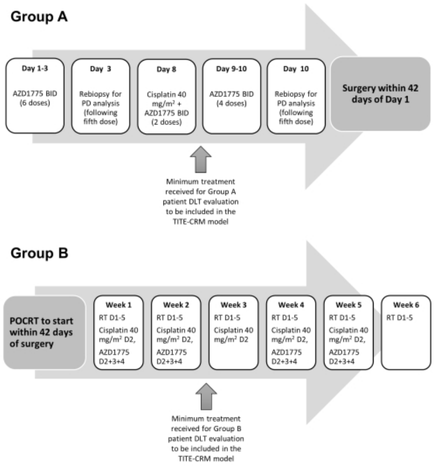

Wisteria trial schema. Group A patients were required to have received a minimum treatment of chemotherapy (cisplatin) and AZD1175 in combination to be DLT evaluable. Group B patients were required to have received a minimum of two weeks of treatment (half the total scheduled AZD1775 dose) to be DLT evaluable.

338x190mm (300 x 300 DPI)

For peer review only - http://bmjopen.bmj.com/site/about/guidelines.xhtml

Appendix 1: Informed Consent Form – Group A

To be printed on hospital headed paper

Informed Consent Form

To be used for Group A only

WISTERIA

A Phase I trial of WEE1 inhibition with Chemotherapy and Radiotherapy as adjuvant treatment, and a Window of Opportunity trial with Cisplatin in Patients with Head and Neck Cancer

EudraCT Reference: 2015-003583-37

Site: **Patient Trial Number:** ☐☐☐

Principal Investigator: ______________________ **Screening Number: SCR** ☐☐ / ☐☐

(If applicable)

Please initial each box

1. I confirm that I have read and understand the Patient Information Sheet Group A (version .... dated ....) for the above trial. I have had the opportunity to consider the information, ask questions and have had these answered satisfactorily. ☐

2. I understand that my participation is voluntary and that I am free to withdraw at any time without giving any reason, without my medical care or legal rights being affected. I understand that if I withdraw from treatment my doctor may continue to provide the Trial Office with information that would routinely be collected about me and recorded in my medical notes. I am aware that I can also withdraw consent for this data transfer. ☐

3. I give permission for my initials, date of birth, and NHS number to be given to the WISTERIA Trial Office when I am registered to the trial as well as a copy of this consent form. ☐

4. I understand that relevant sections of my medical notes and data collected during the trial may be looked at by individuals from the WISTERIA Trial Office, regulatory authorities, Sponsor and/or NHS bodies, where it is relevant to my taking part in this research. I understand that this information will be held in a confidential manner. I give permission for these individuals to have access to my records. ☐

5. I understand that anonymised data from the trial may be provided to other third parties (e.g. pharmaceutical companies or other academic institutions) for research, safety monitoring or licensing purposes. I understand that this may involve sending data outside of the United Kingdom to a European country or the United States of America and that my name will not be given to these third parties.

6. I agree to my GP being informed of my participation in this trial.

7. I understand that the WISTERIA Trial Office may access information held by national cancer registries and within national databases to keep in touch with me and to follow up on my health status.

8. I give permission for collection of samples of my blood and tissue to be used in the WISTERIA trial. I understand that samples will be sent to the Institute of Head and Neck Studies and Education (InHANSE), University of Birmingham and other laboratories in the United Kingdom or overseas (including Covance Laboratories Inc., based in the United States of America).

9. I understand that DNA analysis may be performed on the samples taken for the trial.

10. I consent to the storage of samples remaining at the end of the trial and their use in future ethically approved research which may involve genetic analysis, animal or *in vitro* models, commercial or private institutions, and which may take place in the UK or overseas.

11. I understand that information which may identify me will be transferred outside of the hospital and to the <insert name> Clinical Trials Unit at <insert location>. I give permission for this information to be transferred and for the information, including a copy of this consent form to be held by the Clinical Trials Unit so long as strict confidentiality is maintained.
*To be deleted as appropriate

12. I agree to take part in the above trial.

Name of patient | **Date** | **Signature**

Name of person taking consent | **Date** | **Signature**

You must have signed the
Site Signature & Delegation Log

This document was written using CRCTU-ICF-QCD-001, Version 2.0

CANCER RESEARCH UK

For peer review only

Appendix 2: Informed Consent Form – Group B

To be printed on hospital headed paper

Informed Consent Form

To be used for Group B only

WISTERIA

A Phase I trial of WEE1 inhibition with Chemotherapy and Radiotherapy as adjuvant treatment, and a Window of Opportunity trial with Cisplatin in Patients with Head and Neck Cancer

EudraCT Reference: 2015-003583-37

Site: ____________________________ **Patient Trial Number:** ☐☐☐

Principal Investigator: ____________________________

Please initial each box

13. I confirm that I have read and understand the Patient Information Sheet Group B (version .... dated ....) for the above trial. I have had the opportunity to consider the information, ask questions and have had these answered satisfactorily. ☐

14. I understand that my participation is voluntary and that I am free to withdraw at any time without giving any reason, without my medical care or legal rights being affected. I understand that if I withdraw from treatment my doctor may continue to provide the Trial Office with information that would routinely be collected about me and recorded in my medical notes. I am aware that I can withdraw consent for this data transfer. ☐

15. I give permission for my initials, date of birth, and NHS number to be given to the WISTERIA Trial Office when I am registered to the trial as well as a copy of this consent form. ☐

16. I understand that relevant sections of my medical notes and data collected during the trial may be looked at by individuals from the WISTERIA Trial Office, regulatory authorities, Sponsor and/or NHS bodies, where it is relevant to my taking part in this research. I understand that this information will be held in a confidential manner. I give permission for these individuals to have access to my records. ☐

17. I understand that anonymised data from the trial may be provided to other third parties (e.g. pharmaceutical companies or other academic institutions) for research, safety monitoring or licensing purposes. I understand that this may involve sending data outside of the United Kingdom to a European country or the United States of America and that my will not be given to these third parties. ☐
18. I agree to my GP being informed of my participation in this trial. ☐
19. I understand that the WISTERIA Trial Office may access information held by national cancer registries and within national databases to keep in touch with me and to follow up on my health status. ☐
20. I give permission for the collection of samples of my blood and tissue to be used in the WISTERIA trial. I understand that samples will be sent to the Institute of Head and Neck Studies and Education (InHANSE), University of Birmingham and other laboratories in the United Kingdom or overseas (including Covance Laboratories Inc., based in the United States of America). ☐
21. I understand that DNA analysis may be performed on the samples taken for the trial. ☐
22. I consent to the storage of samples remaining at the end of the trial and their use in future ethically approved research which may involve genetic analysis, animal or *in vitro* models, commercial or private institutions, and which may take place in the UK or overseas. ☐
23. I understand that information which may identify me will be transferred outside of the hospital and to the <insert name> Clinical Trials Unit at <insert location>. I give permission for this information to be transferred and for the information, including a copy of this consent form to be held by the Clinical Trials Unit so long as strict confidentiality is maintained.
*To be deleted as appropriate ☐
24. I agree to take part in the above trial. ☐

____________________	____________________	____________________
**Name of patient**	**Date**	**Signature**

____________________	____________________	____________________
**Name of person taking consent**	**Date**	**Signature**

You must have signed the
Site Signature & Delegation Log

This document was written using CRCTU-ICF-QCD-001, Version 2.0

CANCER RESEARCH UK

For peer review only

Appendix 3: Wisteria Radiotherapy Guidelines – Larynx

<table>
<tr><th>Volume</th><th colspan="2">Definition and description</th></tr>
<tr><td>CTV_6500</td><td colspan="2">Include the operative bed if a positive margin is present, and the nodal levels with extracapsular spread (ECS), if present. The extent of the operative bed is defined with reference to pre-operative imaging of the primary tumour and to the operative pathology.

<u>Primary tumour</u>: The superior border will lie at the level corresponding to the hyoid on the pre-operative imaging for glottic and subglottic tumours, or 1 cm above the most cranial aspect of the tumour, whichever is most cranial. For supraglottic tumours, the superior border will lie at the level of the tip of the epiglottis on pre-operative imaging, or 1 cm superior to the most cranial aspect of the tumour, whichever is most cranial. The inferior border will lie at the level corresponding with the caudal aspect of the cricoid cartilage on pre-operative imaging, or 1 cm inferior to the most caudal aspect of the tumour, whichever is most caudal. The stoma is included in CTV_6500 if there is subglottic extension of the primary tumour and a positive margin, or ECS in level IV.

<u>Nodes:</u> For areas of nodal ECS, the whole level should be included. If a node with ECS is present at a border between two lymph node levels, the level above/below should also be included as appropriate. For nodal levels included in the CTV_6500, the overlying sternocleidomastoid muscle (SCM) should be included.</td></tr>
<tr><td>CTV_6000</td><td colspan="2">Include:
1. The operative bed if there is no positive margin, as defined above.
2. Dissected lymph node levels in which there is no ECS if that side of the neck is pN+. The whole level should be included. The SCM should not be included.
3. The stoma if there are lymph nodes without ECS in level IV, subglottic extension without a positive margin, or an emergency tracheostomy was performed.</td></tr>
<tr><td>CTV_5400</td><td>IA</td><td>Not included</td></tr>
<tr><td></td><td>IB</td><td>Included if ipsilateral level II is node positive</td></tr>
<tr><td></td><td>II</td><td>Always included bilaterally if not already included in CTV_6500 or CTV_6000. If ipsilateral neck is node negative, superior border lies at the inferior border of the transverse process of C1. If ipsilateral neck is node positive, level II is extended superiorly to skull base to include retrostyloid space.</td></tr>
<tr><td></td><td>III</td><td>Always included bilaterally</td></tr>
<tr><td></td><td>IVa</td><td>Always included bilaterally</td></tr>
<tr><td></td><td>V</td><td>Included if ipsilateral neck is node positive</td></tr>
<tr><td></td><td>VI</td><td>Included if there is subglottic extension of the primary tumour, soft tissue extension from the primary into the neck, or hypopharyngeal involvement</td></tr>
<tr><td></td><td>RP*</td><td>Included if bulky ipsilateral nodes present</td></tr>
<tr><td></td><td>Stoma</td><td>If not included in CTV_6500/6000 as defined above</td></tr>
</table>

* In the WISTERIA trial, we recommend that the cranial border of the retropharyngeal nodal level is defined as the upper edge of the body of C1 or the upper extent of the hard palate, whichever is more cranial.

For peer review only

Appendix 4: Wisteria Radiotherapy Guidelines – Hypopharynx

Volume	Definition and description	
**CTV_6500**	Include the operative bed if a positive margin is present, and the nodal levels with ECS, if present. The extent of the operative bed is defined with reference to pre-operative imaging of the primary tumour and to the operative pathology.<br><br>Primary tumour: The superior border will lie at corresponding to the level of the hyoid on pre-operative or 1 cm above the most cranial aspect of the tumour on pre-operative imaging, whichever is most cranial. The inferior border will lie at the level corresponding with the caudal aspect of the cricoid cartilage on pre-operative imaging, or 1 cm inferior to the most caudal aspect of the tumour, whichever is most caudal. The stoma (if present) is included in CTV_6500 if there is subglottic extension of the primary tumour and a positive margin, or ECS in level IV.<br><br>Nodes: For areas of nodal ECS, the whole level should be included. If a node with ECS is present at a border between two lymph node levels, the level above/below should also be included as appropriate. For nodal levels included in the CTV_6500, the overlying sternocleidomastoid muscle (SCM) should be included.	
**CTV_6000**	Include:<br>1. The operative bed if there is no positive margin, as defined above.<br>2. Dissected lymph node levels in which there is no ECS if that side of the neck is pN+. The whole level should be included. The SCM should not be included.<br>3. The stoma (if present) if there are lymph nodes without ECS in level IV, subglottic extension without a positive margin, or an emergency tracheostomy was performed.	
**CTV_5400**	IA	Not included
	IB	Included if ipsilateral level II is node positive
	II	Always included bilaterally if not already included in CTV_6500 or CTV_6000. If ipsilateral neck is node negative, superior border lies at the inferior border of the transverse process of C1. If ipsilateral neck is node positive, level II is extended superiorly to skull base to include retrostyloid space.
	III	Always included bilaterally
	IV	Always included bilaterally
	V	Included if ipsilateral neck is node positive
	VI	Included if there is subglottic/oesophageal extension of the primary tumour, or soft tissue extension from the primary into the neck
		Included
	RP*	If not included in CTV_6500/6000 as defined above
	Stoma	Retropharyngeal (RP)

* In the WISTERIA trial, we recommend that the cranial border of the retropharyngeal nodal level is defined as the upper edge of the body of C1 or the upper extent of the hard palate, whichever is more cranial.

For peer review only

Appendix 5: Wisteria Radiotherapy Guidelines – Lateralised Oral Cavity

Volume	Definition and description	
**CTV_6500**	Include the operative bed if a positive margin is present, and the nodal levels with ECS, if present. The extent of the operative bed is defined with reference to pre-operative imaging of the primary tumour and to the operative pathology.<br><br>Primary tumour: The volume will depend on the primary site. For oral tongue tumours, include the hemi-oral cavity including base of tongue. For buccal mucosal tumours, include the entire mucosa including the retromolar trigone. For retromolar trigone tumours, include the preoperative tumour volume and postoperative tumour bed. Exclude uninvolved bone where possible. Generally, the superior border will be at the level of the hard palate/inferior orbital rim and the inferior border at the level of the hyoid.<br><br>Nodes: For areas of nodal ECS, the whole level should be included. If a node with ECS is present at a border between two lymph node levels, the level above/below should also be included as appropriate. For nodal levels included in the CTV_6500, the overlying sternocleidomastoid muscle (SCM) should be included.	
**CTV_6000**	Include:<br>1. The operative bed if there is no positive margin, as defined above.<br>2. Dissected lymph node levels in which there is no ECS if that side of the neck is pN+. The whole level should be included. The SCM should not be included.	
**CTV_5400**	IA	Included if tumour involves floor of mouth or anterior mandible
	IB	Always included ipsilaterally if not already included in CTV_6500 or CTV_6000
	II	Always included ipsilaterally if not already included in CTV_6500 or CTV_6000. If ipsilateral neck is node negative, superior border lies at the inferior border of the transverse process of C1. If ipsilateral neck is node positive, level II is extended superiorly to skull base. Include contralateral nodes if pN2b/pN3 or primary depth of invasion > 4mm.
		Always included ipsilaterally
	III	Included ipsilaterally if the oropharynx or anterior tongue are involved
	IV	Included if ipsilateral neck is node positive
		Not included
	V	Included if retromolar trigone primary
	VI	Not included
	RP*	Included if retromolar trigone primary

* In the WISTERIA trial, we recommend that the cranial border of the retropharyngeal nodal level is defined as the upper edge of the body of C1 or the upper extent of the hard palate, whichever is more cranial.

Please note: Laterality or otherwise the tumour should be discussed with the surgeon, radiologist and pathologist and confirmed at MDT.

For peer review only

Appendix 6: Wisteria Radiotherapy Guidelines – Non-Lateralised Oral Cavity

Volume	Definition and description	
**CTV_6500**	Include the operative bed if a positive margin is present, and the nodal levels with ECS, if present. The extent of the operative bed is defined with reference to pre-operative imaging of the primary tumour and to the operative pathology.<br><br>Primary tumour: The volume will depend on the primary site. For oral tongue tumours, include the whole oral cavity including base of tongue. Exclude uninvolved bone where possible, but for floor of mouth tumours, consider including the alveolar ridge. Generally, the superior border will be at the level of the hard palate/inferior orbital rim and the inferior border at the level of the hyoid. If a mouth bite is used for floor of mouth tumours, the superior border may be located at the midpoint of the mouthbite.<br><br>Nodes: For areas of nodal ECS, the whole level should be included. If a node with ECS is present at a border between two lymph node levels, the level above/below should also be included as appropriate. For nodal levels included in the CTV_6500, the overlying sternocleidomastoid muscle (SCM) should be included.	
**CTV_6000**	Include:<br>1. The operative bed if there is no positive margin, as defined above.<br>2. Dissected lymph node levels in which there is no ECS if that side of the neck is pN+. The whole level should be included. The SCM should not be included.	
**CTV_5400**	IA	Included if tumour involves floor of mouth or anterior mandible
	IB	Always included bilaterally if not already included in CTV_6500 or CTV_6000
	II	Always included bilaterally if not already included in CTV_6500 or CTV_6000. If ipsilateral neck is node negative, superior border lies at the inferior border of the transverse process of C1. If ipsilateral neck is node positive, level II is extended superiorly to skull base.
		Always included bilaterally.
	III	Included bilaterally if the oropharynx or anterior tongue are involved
	IV	Included if ipsilateral level IV is node positive
		Not included
	V	Consider including if retromolar trigone primary
	VI	
	RP*	Retropharyngeal (RP)

***** In the WISTERIA trial, we recommend that the cranial border of the retropharyngeal nodal level is defined as the upper edge of the body of C1 or the upper extent of the hard palate, whichever is more cranial.

Please note: Laterality or otherwise the tumour should be discussed with the surgeon, radiologist and pathologist and confirmed at MDT.

For peer review only

Appendix 7: Group A data collection schedule

	Pre-diagnosis	Baseline within 42 days of trial entry	Baseline within 21 days of trial entry	Baseline within 14 days of trial entry	Trial Entry	Week 1			Week 2			End of Treatment visit (within 7 days of D10)	4 Week Follow Up visit +/- 3 days	12 Week Follow Up visit (from start of treatment) +/- 3 days
						D1[#*]	D2	D3	D8[#]	D9	D10			
Informed consent – optional tumour sample	x													
Optional tumour sample during diagnostic biopsy	x													
Diagnostic CT scan[a]		x												
Informed consent[b]		x												
ECHO/MUGA[c]			x											
Audiogram[d]			x			As clinically indicated								
Medical history[e]				x										
Physical exam[f]				x		x			x					
Weight (kg) and BSA[g]				x		x			x					
Height (cm)				x										
Vital signs[h]				x		x			x					
ECOG status				x		x			x					
ECG[i]				x		x			x			x		
Pregnancy test[j]				x										
Biochemistry[k]				x		x			x			x	x	x
Haematology[l]				x		x			x			x	x	x
Isotopic GFR[m]				x					x					
Adverse events				x		x			x			x	x	x
Concomitant medications[n]				x		x			x			x	x	x
Patient diary						x			x					
Cisplatin administration									x					
AZD1775 administration[o]						AM & PM doses			AM & PM doses					
Surgery[p]						Within 42 days of first date of treatment								
Blood samples[q]						x						x		x
Pharmacokinetic samples[r]								x			x			
Mandatory tumour biopsy[s]								x				At time of Resection		
Collection of FFPE tumour samples														x
Review of surgical complications														x
Disease free survival[t]						On-going								

Group A data collection schedule notes

- **#** Day 1 and Day 8 should not occur on a Monday.
- ***** Day 1 should be within 6 weeks of informed consent.
- **a** Diagnostic Computerised Tomography (CT) with contrast – neck and chest. To be performed within 42 days of trial entry.
- **b** Informed consent must be obtained before any trial procedures occur.
- **c** If significant cardiac history patient required to have ECHO. MUGA can be performed if ECHO is equivocal. To be performed within 21 days of start of trial entry.
- **d** Air and bone conduction audiogram. To be performed within 21 days of trial entry.
- **e** Medical history – comprehensive medical history, demographics, prior treatment.
- **f** Physical examination includes complete review of systems and physical examination of pertinent organ systems and neurological assessment.
- **g** During trial treatment, weight can be performed within 72 hours pre-dose of AZD1775.
- **h** Blood pressure, pulse measurement, temperature and respiratory rate to be performed with the patient sitting for 5 minutes prior to the evaluation.
- **i** Three ECGs, 2-5 minutes apart, to be performed at screening only. At all other time points a single ECG recording should be performed at least 48h pre-dose of AZD1775.
- **j** Women of childbearing potential will require a negative pregnancy test (serum or urine) prior to trial entry.
- **k** Blood samples for sodium, potassium, magnesium, urea, creatinine, calcium, phosphate, albumin, total protein, bilirubin, alkaline phosphatase, AST and/or ALT.
  Results of blood samples collected at screening visit can be used for Day 1 eligibility if within 72 hours of first dose of AZD1775. During trial treatment, blood samples can be collected within 72 hours **pre-dose** of AZD1775.
- **l** Haematology – Full Blood Count. Results of haematology samples collected at screening visit can be used for Day 1 eligibility if within 72 hours of first dose of AZD1775. During trial treatment, haematology samples can be collected within 72 hours **pre-dose** of AZD1775.
- **m** If creatinine clearance at screening is ≤ 60 ml/min then an isotopic GFR may be carried out and must be > 60 ml/min. If GFR, calculated immediately before any administration of concomitant chemotherapy, is ≤ 60 ml/min then an isotopic GFR may be carried out and must be > 60 ml/min.
- **n** Concomitant medication to be recorded starting on the date of signing of informed consent, throughout the trial.
- **o** Morning dose to be administered prior to cisplatin on Day 8.
- **p** Surgery must be performed within 42 days of first date of trial treatment but at least 6 days after the last cisplatin dose.
- **q** Blood samples will be collected **pre-dose** on Day 1, at the End of Treatment Visit and at the 12 Week Follow Up Visit. Refer to WISTERIA Laboratory Manual.
- **r** Pharmacokinetic samples to be collected before and 2-5 hours after fifth dose of AZD1775 (before the sixth dose) on Day 3 and Day 10. Refer to WISTERIA Laboratory Manual.
- **s** Tumour biopsy to be collected 2-5 hours after fifth dose of AZD1775 (before the sixth dose) on Day 3 and a biopsy to be taken at time of Resection.. To be taken under local anaesthetic (includes biopsy of lymph nodes taken using ultrasound guided biopsy). Time from biopsy taken to fixing in formalin of the biopsy is limited to 30 minutes. Refer to WISTERIA Laboratory Manual.
- **t** Patients to be followed up for 12 months. Notification required if a patient relapses or dies.

For peer review only

Appendix 8: Group B data collection schedule

	Baseline within 21 days of trial entry	Baseline within 14 days of trial entry	Trial Entry	Week 1 – Week 2				Week 3		Week 4 – Week 5				Week 6	Week 7 End of Treatment	Weeks 8-11 Weekly Toxicity Assessments #	Week 12 Follow Up visit	FUP 6 and 12 months from start of treatment
				D1* ~	D2	D3	D4	D1	D2	D1	D2	D3	D4	D1				
Informed consent[a]	x																	
ECHO/MUGA[b]	x																	
Planning CT scan[c]	x																	
Audiogram[d]	x			As clinically indicated														
Medical history[e]		x																
Physical exam[f]		x		x				x		x				x				
Weight (kg) and BSA[g]		x		x				x		x				x				
Height (cm)		x																
Vital signs[h]		x		x				x		x				x				
ECOG status		x		x				x		x				x				
ECG[i]		x		x				x		x				x	x			
Pregnancy test[j]		x																
Biochemistry[k]		x		x				x		x				x	x	x	x	x
Haematology[l]		x		x				x		x				x	x	x	x	x
Isotopic GFR[m]		x		x				x		x								
Adverse events		x		x				x		x				x	x	x	x	x
Concomitant medications[n]		x		x				x		x				x	x	x	x	
Radiotherapy[o]				To start within 3 months of surgery														
AZD1775 administration[p]				N/A	AM & PM Doses			N/A			AM & PM Doses			N/A				
Cisplatin administration[q]					x				x		x			N/A				
Patient diary					x						x							
Quality of Life Questionnaires[r]				x											x		x	x
Swallowing Assessment Questionnaire[r]				x											x		x	x
Blood samples[s]				x											x		x	x
Pharmacokinetic sample[t]							x											
Collection of FFPE tumour samples																	x	
Disease free survival[u]				On-going														

For peer review only

Group B data collection schedule notes:

- **#** Patients will undergo weekly toxicity assessment during treatment and weekly thereafter until all acute toxicities have resolved to Grade 1 or less; typically for 42-56 days (6-8 weeks), and for no less than 42 days after completion of treatment, giving a total Dose Limiting Toxicity reporting period of 84 days (12 weeks) from start of treatment.
- ***** Day 1 of week 1 should be within 4 weeks of consent.
- **~** Week 1 – Day 1 starts on the first day of radiotherapy.
- **a** Informed consent must be obtained before any trial procedures occur.
- **b** If significant cardiac history patient required to have ECHO. MUGA can be performed if ECHO is equivocal. To be performed within 21 days of start of trial entry.
- **c** CT with contrast – neck as part of radiotherapy preparation and planning at Baseline only. To be performed within 21 days of start of trial entry.
- **d** Air and bone conduction audiogram. To be performed within 21 days of trial entry.
- **e** Medical history – comprehensive medical history, demographics, prior treatment.
- **f** Physical examination includes complete review of systems and physical examination of pertinent organ systems and neurological assessment.
- **g** During trial treatment, weight can be performed within 72 hours pre-dose of AZD1775.
- **h** Blood pressure, pulse measurement, temperature and respiratory rate to be performed with the patient sitting for 5 minutes prior to the evaluation.
- **i** Three ECGs, 2-5 minutes apart, to be performed at baseline only. At all other time points a single ECG recording should be performed at least 48 hours **pre-dose** of AZD1775.
- **j** Women of childbearing potential will require a negative pregnancy test (serum or urine) prior to trial entry.
- **k** Blood samples for sodium, potassium, magnesium, urea, creatinine, calcium, phosphate, albumin, total protein, bilirubin, alkaline phosphatase, AST and/or ALT.
  Results of blood samples collected at screening visit can be used for Day 1 eligibility if within 72 hours of first dose of AZD1775. During trial treatment, blood samples can be collected within 72 hours **pre-dose** of AZD1775.
- **l** Haematology – Full Blood Count. Results of haematology samples collected at screening visit can be used for Day 1 eligibility if within 72 hours of first dose of AZD1775. During trial treatment, haematology samples can be collected within 72 hours **pre-dose** of AZD1775.
- **m** If creatinine clearance at screening is ≤ 60 ml/min then an isotopic GFR may be carried out and must be > 60 ml/min. If GFR, calculated immediately before any administration of concomitant chemotherapy, is ≤ 60 ml/min then an isotopic GFR may be carried out and must be > 60 ml/min.
- **n** Concomitant medication to be recorded starting on the date of signing of informed consent, throughout the trial.
- **o** Radiotherapy to start on Week 1 - Day 1. Refer to the WISTERIA Radiotherapy Guidelines.
- **p** AZD1775 morning dose to be administered prior to cisplatin and at least 1 hour prior to radiotherapy.
- **q** Cisplatin to be administered after morning dose of AZD1775 and at least 1 hour prior to radiotherapy.
- **r** Questionnaires to be completed by patient in clinic at defined visits: **pre-dose** on Week 1 - Day 1, End of Treatment Visit, Week 12 Follow Up Visit, 6 Month Follow Up Visit and 12 Month Follow Up Visit.
- **s** Blood samples will be collected **pre-dose** on Week 1 - Day 1, at the End of Treatment Visit, the Week 12 Follow Up Visit, the 6 Month Follow Up Visit and the 12 Month Follow Up Visit. Refer to WISTERIA Laboratory Manual.
- **t** Pharmacokinetic sample to be collected before and 2-5 hours after fifth dose of AZD1775 (before the sixth dose) on Week 1 - Day 4 **only**. Refer to WISTERIA Laboratory Manual.
- **u** Patients to be followed up for 12 months. Notification required if a patient relapses or dies.

Appendix 9: Toxicity Profiles

Treatment	Very common side effects (1 patient in 10)	Common side effects (1 patient in 100)	Other side effects
AZD1775	• Blood & lymphatic disorders including anaemia & thrombocytopenia<br>• Decreased appetite<br>• Gastrointestinal disorders including diarrhoea, nausea, vomiting & serum electrolyte decreases<br>• Dyspepsia	• Neutropenia	• QTc prolongation (rare, affecting 1 patient in 10,000)
AZD1775 in combination with chemotherapy	• Asthenia<br>• Leukopenia<br>• Loss of strength & weakness	• Fatigue<br>• Febrile neutropenia<br>• Mucosal inflammation<br>• Myalgia<br>• Stomatitis	• Constipation<br>• Gastrointestinal haemorrhage<br>• Lymphopenia<br>• Lymphocyte count decrease<br>• Pancytopenia<br>• Sepsis<br>• Tachycardia
Cisplatin	• Blood & lymphatic disorders including bone marrow failure, thrombocytopenia, leukopenia & anaemia<br>• Metabolism & nutrition disorders including dehydration<br>• Renal & urinary disorders including hyperuricaemia	• Sepsis<br>• Ear disorders including ototoxicity<br>• Cardia disorders including arrhythmia, bradycardia & tachycardia<br>• Inflammation at injection site<br>• Respiratory disorders including dyspnoea & pneumonia	
Radiotherapy	• Tiredness<br>• Pain & difficulty swallowing due to mouth ulceration<br>• Thickened saliva & secretions<br>• Dry mouth<br>• Altered sense of taste<br>• Nausea<br>• Skin soreness & ulceration<br>• Hair loss to are near to radiotherapy site		• Due to swallowing difficulties there is an increased risk of chest infection (pneumonia) during treatment

For peer review only

Appendix 10: Haematological Toxicity Dose Management – ANC, Platelets

Symptoms: ANC		Symptoms: Platelets	Action
≥1500/10^9/L	And	≥75000/10^9/L	No cisplatin or AZD1775 dose modification or interruption.
<1500/10^9/L	Or	<75000/10^9/L	Delay cisplatin and AZD1775 by 1 week intervals until recovery. If DLT is reached, discontinue AZD1775 treatment but cisplatin may be resumed if deemed appropriate by the investigators.

Appendix 11: Haematological Toxicity Dose Management – Neutropenia, Infection, Febrile Neutropenia

Symptoms	Action
Grade 3 febrile neutropenia (ANC <1000/10^9/L + Temperature ≥38°C) or neutropenic sepsis<br><br>Grade 4 neutropenia (ANC <500/10^9/L >7 days)<br><br>Grade 4 thrombocytopenia (platelet count <25,000/10^9/L >7 days)	Hold cisplatin and AZD1775 dose until recovery. If DLT is reached, discontinue AZD1775 treatment but cisplatin may be resumed if deemed appropriate by the investigators.
Grade 4 febrile neutropenia or Grade 4 infection with neutropenia (both defined as septic shock)<br><br>Thrombocytopenic haemorrhage (gross occult bleeding) associated with a platelet count <50,000/10^9/L	Discontinue cisplatin and AZD1775 treatment.
Febrile neutropenia (≥ 38° C) with or without significant symptoms	Patient to be managed in a hospital setting according to standard procedures, with the urgent initiation of IV antibiotic therapy according to local guidelines.

For peer review only

Appendix 12: Non-Haematological Toxicity Dose Management – General Disorders

CTCAE v4.03	Cisplatin	AZD1775
**Grade 0-2**	No dose modification	No dose modification
**Grade 3**	Hold until toxicity resolves to ≤ Grade 1, and then resume at the same dose with no modification if no DLT is reached and if deemed appropriate by the investigators.	Hold until toxicity resolves to ≤ Grade 1, and then resume at the same dose with no modification. Discontinue treatment if DLT is reached
**Grade 4 toxicity** (except anorexia)	Discontinue treatment	Discontinue treatment

Appendix 13: Non-Haematological Toxicity Dose Management – Hepatic

CTCAE v4.03	Cisplatin	AZD1775
**Grade 1-2**	No dose modification	No dose modification
**Grade 3** (manifested as elevations in ALT, AST, ALP or bilirubin)	Hold until resolves to Grade ≤1 or baseline, then resume cisplatin with no dose modification if no DLT is reached and if deemed appropriate by the investigators.	Hold until resolves to Grade ≤1 or baseline, then resume study drug with no dose reduction. If not resolved within 28 days discontinue study drug. Discontinue treatment if DLT is reached.
**Grade 4** (Life threatening)	Discontinue treatment	Discontinue treatment

Appendix 14: Non-Haematological Toxicity Dose Management – Diarrhoea or Mucositis

CTCAE v4.03	Cisplatin	AZD1775
**Grade 3** (requiring hospitalisation)	Hold until toxicity resolves to ≤ Grade 1, and then resume at the same dose with no modification if no DLT is reached and if deemed appropriate by the investigators.	Hold until toxicity resolves to ≤ Grade 1, and then resume at the same dose with no modification. Discontinue treatment if DLT reached.
**Grade 4**	Discontinue treatment	Discontinue treatment

For peer review only

Appendix 15: Non-Haematological Toxicity Dose Management – Renal Toxicity

CTCAE v4.03	Cisplatin	AZD1775
**Grade ≥ 2**	Hold until resolves to Grade ≤1 or baseline. If GFR falls below 60 mL/min, cisplatin should be discontinued and consideration given to substitution with carboplatin AUC =1.5, according to routine local practice.	Hold until toxicity resolves to ≤ Grade 1, and then resume at the same dose with no modification. Discontinue treatment if DLT reached

Appendix 16: Non-Haematological Toxicity Dose Management – Neurotoxocity

CTCAE v4.03	Cisplatin	AZD1775
**Grade 1**	No dose modification	No dose modification
**Grade 2**	Hold until toxicity resolves to Grade ≤1. Resume with 1 dose level reduction.	No dose modification
**Grade 3 or 4**	Discontinue treatment	Discontinue treatment

Appendix 17: Disallowed medications and medication to be administered with caution

A list of the main CYP3A4 substrates, inhibitors (strong and moderate) and inducers, CYP2C19 substrates, P-gp substrates and inhibitors and BCRP substrates are shown below:
Please note: Live vaccines are not permitted

This is not an exhaustive list and further details can be found at:
www.fda.gov/drugs/developmentapprovalprocess/developmentresources/druginteractionslabeling/ucm093664.htm

CYP3A4 Inhibitors (Strong)

Boceprevir
Clarithromycin
Cobicistat (GS-9350)
Conivaptan
Danoprevir
Elvitegravir
Fosamprenavir
Grapefruit juice
Idelalisib
Indinavir
Itraconazole
Ketoconazole
LCL161
Lopinavir
Mibefradil
Nefazodone
Nelfinavir
Posaconazole
Ritonavir
Saquinavir
Telaprevir
Telithromycin
Tipranavir
Troleandomycin
Voriconazole

For peer review only

CYP3A4 Inhibitors (Moderate)

- ACT-178882
- Amprenavir
- Aprepitant
- Atazanavir
- Casopitant
- Ciprofloxacin
- Crizotinib
- Darunavir
- Dronedarone
- Diltiazem
- Erythromycin
- FK1706
- Fluconazole
- Fosamprenavir
- Imatinib
- Ledipasvir
- Lomitapide
- Netupitant
- Schisandra sphenanthera
- Tofisopam
- Verapamil

CYP3A4 Inhibitors (Weak)

- Almorexant
- Alprazolam
- AMD070
- Amiodarone
- Amlodipine
- Atorvastatin
- Azithromycin
- Berberine
- Bicalutamide
- Blueberry juice
- Chlorzoxazone
- Cilostazol
- Cimetidine
- Clotrimazole
- Cranberry juice
- Cyclosporine
- Daclatasvir
- Delavirdine
- Everolimus
- Faldaprevir
- Fluvoxamine
- Fosaprepitant (IV)
- Ginkgo
- Goldenseal
- GSK1292263
- GSK2248761
- Isoniazid
- Ivacaftor
- Lacidipine
- I Linagliptin
- Lomitapide
- M100240
- Nilotinib
- Oral contraceptives
- Pazopanib
- Peppermint oil
- Propiverine
- Ranitidine
- Ranolazine
- Resveratrol
- Roxithromycin
- Seville orange juice
- Simeprevir
- Sitaxentan
- Suvorexant
- Tabimorelin
- Tacrolimus
- Teriflunomide
- Ticagrelor
- Tipranavir/ritonavir
- Tolvaptan
- Zileuton

CYP3A4 Inducers (Strong and Moderate)

- Avasimibe
- Bosentan
- Carbamazepine
- Efavirenz
- Enzalutamide
- Etravirine
- Genistein
- Lersivirine
- Lopinavir
- Mitotane
- Modafinil
- Nafcillin
- Phenobarbital
- Phenytoin
- Rifabutin
- Rifampin
- Ritonavir
- Semagacestat
- St John's Wort
- Thioridazine
- Tipranavir

CYP3A4 Inducers (Weak)

- Amprenavir
- Aprepitant
- Armodafinil
- AZD 7325
- Bexarotene
- Boceprevir
- Brivaracetam
- Clobazam
- Danshen
- Dexamethasone
- Echinacea
- Eslicarbazepine
- Garlic
- Gingko
- Ginseng
- Glycyrrhizin
- LCL161
- Methylprednisolone
- Nevirapine
- Oritavancin
- Oxcarbazepine
- PA-824
- Pleconaril
- Prednisone
- Quercetin
- Raltegravir
- Ritonavir
- Rufinamide
- Sorafenib
- Stribild

For peer review only

Telaprevir
Terbinafine
Ticagrelor
Ticlopidine
Topiramate
Troglitazone
Vemurafenib
Vicriviroc and ritonavir
Vinblastine

CYP3A and CYP3A4 Sensitive Substrates or Substrates with a Narrow Therapeutic Range

ABT-384
Alfentanil
Aprepitant
Alfuzosin
Almorexant
Alpha-Dihydroergocryptine
Amiodarone
Aplaviroc
Aprepitant
Astemizole
Atazanavir
Atorvastatin
Avanafil
Bexarotine
BIRL 355
Bortezomib
Bosutinib
Brecanavir
Brotizolam
Budesonide
Buspirone
Capravirine
Carbamazepine
Casopitant
Cisapride,
Conivaptan
Cyclophosphamide
Cyclosporine
Danoprevir
Darifenacin
Darunavir
Dasatinib
Dihydroergotamine
Disopyramide
Dronedarone
Docetaxol
Dofetilide
Doxorubicin
Ebastine
Eletriptan
Elvitegravir
Eplerenone
Ergotamine
Erlotinib
Etoposide
Everolimus
Felodipine
Fentanyl
Fluticasone
Gefitinib
Halofantrine
Ibrutinib
Ifosfamide
Imatinib
Indinavir
Ironotecan
Ivacaftor
Ixabepilone
L-771,688
Lapatinib
Levomethadyl (LAAm)
Lomitapide
Lopinavir
Lovastatin
Lurasidone
Maraviroc,
Midazolam
Midostaurin
Mosapride
Neratinib
Nilotinib
Nisoldipine
Paclitaxel
Pazopanib
Perospirone
Pimozide
Propafenone
Propofol
Quetiapine
Quinidine
Ranolazine
Ridaforolimus
Romidepsin
Saquinavir
Sildenafil
Simeprevir
Simvastatin
Sirolimus
Tacrolimus
Temsirolimus
Terfenadine
Ticagrelor
Theophylline
Thioridazine
Thiotepa
Tilidine
Tipranavir
Tolvaptan
Triazolam
Tretinoin
Ulipristal
Vardenafil
Vicriviroc
Voclosporin

CYP2C19 Sensitive Substrates or Substrates with a Narrow Therapeutic Range

Diazepam
Gliclazide
Lansoprazole
(R)-Lansoprazole
(S)-Lansoprazole
(S)-Mephenytoin
(R)-Mephobarbital
Omeprazole
(R)-Omeprazole
Pantoprazole
(+)-Pantoprazole
Rabeprazole
Tilidine

CYP1A2 Sensitive Substrates or Substrates with a Narrow Therapeutic Range

For peer review only

Alosetron	Melatonin	Theophylline
Caffeine	Ramelteon	Tizanidine
Duloxetine	Tacrine	

P-gp Substrates

Colchicine	Indinavir	Vincristine
Digoxin	Paclitaxel	
Fexofenadine	Toptecan	

If a patient requires initiation of digoxin during the study, or is already receiving treatment with digoxin, monitoring of digoxin levels is recommended according to local practice (as the levels of digoxin may increase). Monitoring of digoxin levels is also recommended when the patient has completed dosing with study treatment (as the levels of digoxin may then decrease).

**P-gp Inhibitors (Strong)**	**BCRP Substrates**
Cyclosporine	Daunorubicin
Elacridar	Doxorubicin
Erythromycin	Rosuvastatin
Itraconazole	Sulfasalazine
Ketocoanzole	Topotecan
LY335979Quinidine	
Ritonavir	
Valspodar	
Verapamil	

For peer review only

Wisteria Trial SPIRIT Checklist

Section/Item	Item Number	Placement of Information
**Administrative information**		
Title	1	Front page
Trial registration	2a	Within Abstract
	2b	Within Abstract and Funding sections
Protocol version	3	Within Ethics and Dissemination section
Funding	4	Funding section
Roles and responsibilities	5a	Front page, Author Affiliations, Authors' Contributions sections
	5b	Front page
	5c	Trial Organisation and Structure section
	5d	Trial Organisation and Structure section
**Introduction**		
Background and rationale	6a	Introduction sections
	6b	N/A
Objectives	7	Methods: Wisteria Trial Objectives section
Trial design	8	Methods: Trial Design section
**Methods**		
Participants, interventions and outcomes:		
Study setting	9	Patient Population, Screening and Consent section
Eligibility criteria	10	Patient Population, Screening and Consent section, boxes 2 and 3
Interventions	11a	Dose Selection section, Table 1, Trial Treatment section
	11b	Dose Limiting Toxicity and Dose Management section, Discontinuation of Investigational Medicinal Product section, Statistical Methods section
	11c	Trial Schema, Trial Treatment section
	11d	Concomitant Medications section, Appendix 11
Outcomes	12	Trial Outcome Measures section, Box 4, Appendices Tables 1 and 2
Participant timelines	13	Trial Schema, Appendices Tables 1 and 2
Sample size	14	Trial Design section, Statistical Methods section
Recruitment	15	Trial Design section
Assignment of interventions (for controlled trials)	16 -17	Not applicable
Data collection, management and analysis:		
Data collection methods	18a	Data Collection and Monitoring section, Appendices 1 and 2
	18b	Analysis of Outcome Measures section
Data management	19	Data Collection and Monitoring section
Statistical methods	20	Statistical Methods section, Analysis of Outcome Measures section
Monitoring:		
Data monitoring	21a	Trial Organisation and Structure section
	21b	Statistical Methods section, Analysis of Outcomes section

For peer review only

Harms	22	Box 5, Safety Monitoring section
Auditing	23	Data Collection and Monitoring section, Trial Organisation and Structure section
**Ethics and dissemination**		
Research ethics approval	24	Ethics and Dissemination abstract and main body section, Ethical Approval section
Protocol amendments	25	Ethics and Dissemination section
Consent or assent	26a	Patient Consent section, Appendices 16 and 17
	26b	Ethics and Dissemination section
Confidentiality	27	Ethics and Dissemination section
Declaration of interests	28	Competing Interests section
Access to data	29	Ethics and Dissemination section, Open Access section
Ancillary and post-trial care	30	Trial Treatment section, Box 4, (re. follow up)
Dissemination policy	31a	Ethics and Dissemination section, Open Access section
**Appendices**		
Informed consent materials	32	Appendices 16 and 17
Biological specimens	33	Tertiary Research Outcome Measures section, Appendices Tables 1 and 2, Analysis of Outcome Measures section

For peer review only
